# The Yeast Metabolic Cycle as a Tractable Cellular Framework for Redox Timing, Redox Buffering, and Transcriptome Fidelity

**DOI:** 10.3390/antiox15080914

**Published:** 2026-07-23

**Authors:** Ondrej Preťo, Bogdan Iaparov, Friedemann Freund, Miloslav Karhanek, Viktor Stolc

**Affiliations:** 1 Institute of Experimental Endocrinology, Biomedical Research Center, Slovak Academy of Sciences, 845 05 Bratislava, Slovakia; 2 Laboratory of Bioinformatics, Biomedical Research Center, Slovak Academy of Sciences, 845 05 Bratislava, Slovakiamiloslav.karhanek@savba.sk (M.K.); 3 SETI Institute, Mountain View, CA 94043, USA

**Keywords:** yeast metabolic cycle, redox timing, redox buffering, transcriptome fidelity, RNA-seq mismatch, RNA–DNA differences, sequence discordance, mitochondrial respiration, GLMM

## Abstract

The yeast metabolic cycle (YMC) in *Saccharomyces cerevisiae* provides a tractable model for examining how mitochondrial respiration, redox timing, and metabolic phase shape transcriptome abundance and fidelity. Ribosomal-RNA-depleted whole-transcriptome RNA sequencing (WRS) and RNA-seq-derived mismatch analyses were performed across low-dissolved-oxygen (Low-DO)/high-respiration and high-dissolved-oxygen (High-DO)/lower-respiration phases. Among 1505 phase-differentially expressed genes, Low DO was enriched for ribosome biogenesis, rRNA processing, translation, sulfur metabolism, and protein synthesis, whereas High DO was enriched for oxidant detoxification, oxidoreductase activity, and redox-buffering-related pathways. Generalized linear mixed models identified a substitution-class-dependent Low-DO-associated RNA-seq mismatch response. The strongest mismatch-level increase occurred in the collapsed C > T/G > A-compatible class, whereas C > A/G > T did not increase. This pattern was not consistent with a simple single-lesion model and instead supported a mixed Low-DO-associated RNA-seq sequence-discordance landscape. Variant-rate modeling additionally detected T > C/A > G and T > A/A > T increases, indicating that multiple biological and technical processes may contribute to the observed spectrum. Recurrence analysis showed that most called variants were sample-specific, supporting a transient RNA-seq mismatch landscape rather than stable DNA mutation. These findings establish the YMC as a reductionist eukaryotic framework for studying how metabolic phase and redox state shape transcriptome fidelity.

## 1. Introduction

The flow of biological information from DNA to RNA to protein is increasingly understood as a spatially and temporally organized process rather than a linear biochemical sequence. In eukaryotic cells, transcription, RNA processing, nuclear export, translation, decay, and quality control occur within a dynamic nuclear and cytoplasmic architecture [1,2]. At the same time, cellular metabolism is not constant. Energy production, redox state, redox-buffering demand, and biosynthetic activity fluctuate across biological cycles, creating recurring windows in which macromolecules may be differentially protected or vulnerable [3,4,5,6,7,8,9].

The yeast metabolic cycle (YMC) provides an experimentally tractable model for studying how biological timing, mitochondrial activity, redox buffering, and transcriptome regulation are coordinated. In continuous glucose-limited chemostat cultures, *Saccharomyces cerevisiae* undergoes synchronized oscillations in dissolved oxygen (DO), respiration, ATP production, redox state, and gene expression [3,4,5,6,7,9]. These oscillations reveal a temporal organization of yeast physiology in which anabolic growth, stress defense, and genome-protective processes are phased across recurring metabolic states [8,9,10].

Genetically modified yeasts can exhibit rewired metabolic pathways and altered regulatory states, and such systems are valuable for studying engineered metabolic flux. The present study, however, used the non-engineered YMC strain DBY12007 in a glucose-limited chemostat to examine endogenous metabolic oscillation rather than synthetic pathway engineering. This distinction is important because the objective was to study naturally synchronized redox-phase biology in a canonical YMC model.

A key feature of the YMC is the temporal separation of biosynthetic and genome-protective functions. During the Low-DO phase, dissolved oxygen in the medium reaches a nadir because cellular oxygen consumption is maximal—the cells are effectively “inhaling” oxygen from the medium at the highest rate. Low DO can therefore be understood as consumption-driven hypoxia, distinct from primary or supply limited hypoxia caused by inadequate oxygen delivery. In clinical terms, it is more analogous to a high-extraction state in which oxygen tension falls because metabolic demand is elevated. This phase corresponds to the canonical Ox/high-oxygen-consumption state and is characterized by increased mitochondrial respiration, ATP production, ribosome biogenesis, and a more oxidized intracellular redox environment [5,6,7,9]. By contrast, the High-DO phase reflects greater residual oxygen in the medium because cellular oxygen consumption has declined and is associated with lower respiratory activity and a more reductive metabolic state [5,6,7,9].

The DO trace should therefore be interpreted as an inverse readout of respiratory demand: it measures the oxygen remaining in the medium, not oxygen flux through mitochondria. Accordingly, the upstream redox driver in this study is inferred to be the Low-DO/high-respiration phase. During this interval, increased mitochondrial electron flux can increase electron leakage from the respiratory chain, generating superoxide and downstream hydrogen peroxide and thereby increasing respiratory ROS pressure relative to High DO [7,9]. The subsequent High-DO phase is enriched for detoxification, oxidoreductase, and redox-buffering programs, plausibly reflecting recovery from the preceding period of intense respiration [3,4,5,6,7]. Although intracellular ROS was not measured directly, this distinction between consumption-driven hypoxia and reduced oxygen delivery provides the mechanistic basis for interpreting the Low-DO-associated RNA-seq mismatch patterns.

DNA replication is restricted to the reductive phase of the YMC, a timing strategy that helps protect genome integrity by avoiding replication during periods of elevated respiratory oxidative stress [10]. Transcription, however, continues through the Low-DO/high-respiration phase to support ribosome biogenesis, metabolic remodeling, stress responses, and biosynthetic demands [8,9]. This creates a potential window of transcriptome vulnerability in which RNA molecules—and particularly those produced or processed near mitochondria-rich regions or the nuclear envelope—may be differentially exposed to reactive oxygen species, owing to local concentration gradients rather than a uniform cellular ROS burden.

This phase-dependent vulnerability is conceptually aligned with the redox-cycle resonance model proposed by Stolc, Shmygelska, and Griko, in which RNA transcription was hypothesized to resonate temporally with the oscillating reduction–oxidation state of the cell and thereby create non-random sequence vulnerability at specific genomic coordinates [11]. In that framework, the redox cycle functions as a temporal template: genes transcribed during more oxidizing phases experience a different chemical environment than genes transcribed during more reductive phases. The model therefore predicts that sequence variation, RNA mismatch formation, or RNA damage-compatible readout should not be distributed uniformly across the genome or transcriptome, nor should they be explained by expression level alone. Instead, vulnerability should depend on the phase relationship among transcriptional timing, redox state, redox-buffering capacity, and local molecular context. The present study revisits this principle using modern ribosomal-RNA-depleted RNA-seq and replicate-aware mismatch-rate and variant-rate modeling across defined Low-DO and High-DO phases of the YMC.

RNA-seq sequence discordance is biologically important because transcriptional errors, chemically modified nucleosides, and damaged RNA templates can affect translation, RNA turnover, protein quality, stress signaling, and cellular adaptation without requiring stable DNA mutation [12,13,14,15]. Related studies have characterized oxidative DNA damage, oxidized or deaminated cytosines, base-excision repair, transcriptional mutagenesis, and metabolism-associated mixed substitution spectra [16,17,18,19,20,21]. However, RNA-seq-derived mismatches are indirect readouts rather than direct chemical measurements. The same substitution class may arise from several biological or technical processes, including base modification, transcriptional misincorporation, reverse-transcription behavior, mapping ambiguity, library chemistry, or residual DNA-level variation [12,15,19,22,23,24]. Substitution spectra must therefore be interpreted as mechanistically compatible patterns rather than as definitive signatures of individual lesions. Notably, classic 8-oxoguanine-mediated lesions are most strongly associated with G:C → T:A transversions (G > T/C > A) rather than C > T transitions [25].

The broader mechanistic framework for interpreting such RNA-seq mismatch patterns is provided by recent work on RNA–DNA differences (RDDs), which emphasizes that RNA sequence discordance can arise through multiple overlapping processes, including canonical RNA editing, transcriptional misincorporation, oxidative nucleotide chemistry, post-transcriptional RNA damage, and technical readout effects [24]. This distinction is important for the present study because the YMC mismatch landscape should not be interpreted as a simple catalog of RNA “mutations”. Rather, phase-dependent mismatch and variant-rate shifts are best understood as RNA sequence-discordance signals that may reflect redox-associated chemistry, oxidized nucleotide pools, RNA modification, altered RNA processing, reverse-transcription behavior, transcript-composition effects, or library-dependent readout [24].

*S. cerevisiae* lacks the canonical RNAi machinery found in many other eukaryotes [26], and no extensive endogenous ADAR-mediated editing system has been established in this species. Canonical A-to-I editing is therefore unlikely to be a major explanation for the observed mismatch landscape, although the RNA-seq data do not distinguish among alternative biological and technical mechanisms.

Dissolved oxygen in chemostat cultures can be measured by electrochemical polarographic or optical sensors and is commonly used as a real-time proxy for the respiratory phase in YMC experiments. In the present study, DO was used as a phase marker for sampling; it was not used as a direct measurement of mitochondrial oxygen flux, intracellular ROS, or oxidative damage.

This study focuses on ribosomal-RNA-depleted whole-transcriptome RNA sequencing (WRS) and RNA-seq-derived mismatch analysis across Low-DO and High-DO phases. We first define the phase-specific transcriptome using DESeq2 and pathway enrichment analysis [27,28]. We then quantify mismatch-level and variant-level substitution-class responses using callable-site analysis, beta-binomial GLMMs for the mismatch rate, negative binomial GLMMs for the variant rate, and recurrence-degree analysis across biological replicates [29,30]. This design tests whether the transition to Low DO/high respiration is associated with a reproducible shift in RNA-seq-derived mismatch patterns and whether the resulting response differs among substitution classes [12,15,31].

Although this study does not directly model human disease, sleep physiology, or psychiatric disorders, the YMC provides a simplified eukaryotic system for testing a general cellular principle: metabolic phase and redox state can create temporally structured windows of transcriptomic vulnerability. This yeast framework may inform future studies in mammalian systems where circadian timing, intermittent hypoxia, mitochondrial demand, inflammation, and redox buffering interact dynamically.

## 2. Materials and Methods

### 2.1. Yeast Strain, Bioreactor Setup, and Metabolic Synchronization

*S. cerevisiae* strain DBY12007, a diploid prototroph (MATa/MATα) in the S288C background with wild-type *HAP1* alleles (*HAP1*+), was cultivated in a BioFlo 310 bioreactor (New Brunswick Scientific, Edison, NJ, USA) at a working volume of 1.0 L. DBY12007 was originally derived by Hickman and Winston and subsequently characterized in YMC studies from the Botstein laboratory, including Silverman et al. and Slavov and Botstein. The strain was obtained from the Gibney Lab, Cornell University, Ithaca, NY, USA, the current custodian of the David Botstein DBY strain collection. A Rushton-type impeller was operated at 500 rpm, and aeration was supplied at 5 L/min through a bottom sparger. Temperature was maintained at 30 °C, pH at 4.0 by automated addition of 2 M NaOH and 2 M phosphoric acid, and dissolved oxygen was continuously monitored with the bioreactor polarographic DO probe but not actively controlled. The DO signal was used as a real-time phase marker for respiratory cycling and sampling; it did not directly measure mitochondrial oxygen flux, intracellular ROS concentration, or oxidative damage. This continuous-culture strategy follows the established use of glucose-limited chemostats to synchronize and analyze the YMC [5,6,7,9].

A defined minimal medium based on established glucose-limited YMC chemostat protocols was used throughout. The base composition was 5 g/L (NH_4_)_2_SO_4_, 2 g/L KH_2_PO_4_, 0.5 g/L MgSO_4_·7H_2_O, 0.1 g/L CaCl_2_·2H_2_O, 0.02 g/L FeSO_4_·7H_2_O, 0.01 g/L ZnSO_4_·7H_2_O, 0.005 g/L CuSO_4_·5H_2_O, 0.001 g/L MnCl_2_·4H_2_O, and 1 g/L yeast extract, acidified with 0.5 mL/L concentrated sulfuric acid (unless otherwise specified, all chemicals and reagents were obtained from Sigma-Aldrich (St. Louis, MO, USA); potassium phosphate monobasic KH_2_PO_4_ and magnesium sulfate heptahydrate MgSO_4_·7H_2_O were obtained from Merck (Darmstadt, Germany); calcium chloride dihydrate CaCl_2_·2H_2_O was obtained from Centralchem (Bratislava, Slovakia). Ammonium sulfate, phosphate, magnesium, calcium, and trace metals were included to support respiratory growth, enzyme function, and stable chemostat physiology under glucose limitation. Concentrated sulfuric acid was added at 0.5 mL/L to acidify the feed medium and maintain trace-metal solubility. At this dilution, sulfuric acid was not used as an oxidative treatment. During chemostat operation, pH was actively maintained at 4.0 by the automated addition of NaOH and phosphoric acid rather than by a buffer system.

Shake-flask precultures and the initial batch phase contained 11 g/L D-glucose monohydrate (Fluka Analytical; Honeywell Research Chemicals, Seelze, Germany; Cat. No. 49161); the continuous-feed medium contained 4.4 g/L D-glucose monohydrate. Antifoam 204 (Sigma-Aldrich, St. Louis, MO, USA; Cat. No. A6426) was added to the bioreactor cultures and feed medium.

A frozen glycerol stock of DBY12007 was used to inoculate 100 mL of preculture medium in a 250 mL shake flask, which was incubated for 24–48 h at 30 °C. Cells were harvested by centrifugation, resuspended in fresh medium, and transferred to the bioreactor. Following inoculation, the culture was grown in batch mode until DO returned to nearly 100%, indicating carbon-source depletion. Cultures were then held under starvation for at least 6 h. Continuous cultivation was initiated at a dilution rate of D = 0.1 h^−1^ using an external peristaltic pump (Watson-Marlow 120U, Watson-Marlow Fluid Technology Solutions, Falmouth, UK), and culture volume was maintained at 1.0 L by withdrawal through an effluent port at fixed depth, controlled by the external peristaltic pump.

Successful metabolic synchronization was defined as the emergence of reproducible DO oscillations, with an amplitude of approximately 20 percentage points and a period of 90–100 min, within 6 h of feed initiation. If synchronization was not achieved, an additional starvation cycle was applied; up to three to four such cycles were attempted before terminating the experiment. Low-DO samples were collected at the dissolved oxygen nadir, corresponding to the high-respiration phase, and High-DO samples were collected at the dissolved oxygen peak, corresponding to the lower-respiration phase [5,6,7,9].

### 2.2. RNA Extraction

For ribosomal-RNA-depleted whole-transcriptome RNA sequencing (WRS), 1 mL of culture was withdrawn at the designated phase time point, pelleted, resuspended in RNAlater™ (Invitrogen, Thermo Fisher Scientific, Waltham, MA, USA), incubated at room temperature, flash-frozen in liquid nitrogen, and stored at −80 °C. Total RNA was extracted using the RiboPure™-Yeast Kit (Thermo Fisher Scientific, Waltham, MA, USA) according to the manufacturer’s protocol, including post-elution DNase I (part of the RiboPure kit) treatment to remove residual genomic DNA. RNA concentration and purity were assessed using NanoDrop One spectrophotometry (Thermo Fisher Scientific, Waltham, MA, USA) when sufficient material was available. For WRS libraries, ribosomal RNA was depleted using a RiboCop rRNA Depletion Kit for Yeast (Lexogen, Vienna, Austria). Libraries were then prepared using the CORALL Total RNA-Seq V2 kit (Lexogen, Vienna, Austria) according to the manufacturer’s protocol. Libraries were sequenced on the Element AVITI24 (Element Biosciences, San Diego, CA, USA) platform in paired-end mode (2 × 150 bp) to a target depth of ≥40 million read pairs per sample. RNA concentration and spectrophotometric purity ratios are summarized in Table 1.

### 2.3. Software Environment

All bioinformatic analyses were performed on a 64 bit Linux workstation. Primary alignment and quantification were carried out using STAR v2.7.11b and Subread featureCounts v2.1.1 [32,33]. Differential expression and enrichment analyses were performed using DESeq2 v1.46 and clusterProfiler v4.18.4 [27,28]. RNA mismatch counting and file processing used SAMtools v1.18 and BCFtools v1.18 [34]. Variant-level analysis used GATK/Mutect2 [35]. GLMM analyses were performed using glmmTMB v1.1.14 [29,30]. Figures were generated using ggplot2 v4.0.3 [36]. Scripts were version-controlled and are available in the project repository.

### 2.4. Reference Genome and Gene Annotation

RNA-seq reads were aligned to the standard S288C sacCer3 reference genome. Gene annotations were derived from the Saccharomyces Genome Database S288C release R64-4-1 annotation, comprising 6607 annotated features across the 16 nuclear chromosomes and mitochondrial genome. Reads were aligned to the canonical S288C reference rather than to a DBY12007-specific genome in order to leverage the mature S288C gene annotation, existing functional resources, and compatibility with future comparative and meta-analyses. This choice inevitably leaves a small number of strain-specific polymorphisms, which are mitigated and are expected to have only a minor, symmetric impact on the rate estimates.

### 2.5. Read Alignment with STAR

Paired-end WRS reads were aligned to the S288C/sacCer3 reference genome using STAR v2.7 [32]. The STAR genome index was built using the R64-4-1 gene annotation, with sjdbOverhang selected according to read length. Alignment was performed with standard paired-end RNA-seq parameters and coordinate-sorted BAM files were generated. Alignment statistics, including uniquely mapped read percentage, multimapping rate, and unmapped fraction, were recorded for each sample and reviewed for quality control.

### 2.6. Gene-Level Quantification with featureCounts

Gene-level read counts were generated using featureCounts from Subread v2.1.1 [33]. Libraries were processed as paired-end data using the -p flag, counting read pairs rather than individual reads. Only primary alignments were counted using –primary, and multi-overlapping reads were excluded. Library strandedness was set to stranded (-s 1), corresponding to the FR_SECONDSTRAND ligation-based protocol used for library preparation, in which read 1 maps to the sense strand of the transcript.

Counts were summarized at the meta-feature/gene level using the R64-4-1 annotation, producing count matrices with genes as rows and samples as columns. The WRS dataset consisted of six samples, comprising three Low-DO and three High-DO biological replicates.

### 2.7. Differential Expression Analysis of WRS RNA-Seq

Differential expression analysis of WRS data between Low-DO and High-DO phases was performed using DESeq2 v1.46 [27]. Raw featureCounts matrices were loaded directly into DESeq2 without prior normalization. Genes with low total counts across all samples were removed before model fitting according to the filtering criteria used in the analysis scripts. Size factors were estimated using the median-of-ratios method, and dispersion estimates were obtained using the DESeq2 empirical Bayes shrinkage procedure.

The design matrix modeled the metabolic phase—Low DO versus High DO—as the explanatory factor. Wald test *p* values were computed for each gene and adjusted for multiple testing using the Benjamini–Hochberg false discovery rate procedure. Genes were considered significantly differentially expressed at adjusted *p* < 0.05. For downstream phase-enrichment analyses, an additional fold-change threshold of |log2FC| > 1 was applied. Variance-stabilized counts were used for principal component analysis and heatmap visualization.

For biological interpretation, differentially expressed genes were filtered using adjusted *p* < 0.05 and |log_2_FC| > 1. The absolute-value threshold was used because both directions of differential expression were biologically informative: positive log_2_FC values identify Low-DO-enriched genes, whereas negative log_2_FC values identify High-DO-enriched genes. The two-fold effect-size cutoff was used to focus the interpretation of individual differentially expressed genes on changes likely to be biologically relevant and to reduce the influence of statistically significant but small-magnitude differences driven by low within-group variance. The DESeq2 results for the top 100 most significantly differentially expressed genes are provided in Supplementary Table S1.

### 2.8. Gene Ontology and Gene Set Enrichment Analyses

Gene Set Enrichment Analysis (GSEA) was performed using clusterProfiler v4.18.4 [28]. Gene identifiers in the DESeq2 results were harmonized to those used in the Saccharomyces cerevisiae Gene Association File (sgd.gaf.gz, see Data Availability Statement) generated by GO Consortium on 27 January 2026. This file contains associations between yeast gene products and Gene Ontology terms and was used to construct the Biological Process and Molecular Function gene sets for GSEA. A pre-ranked gene list was generated based on the direction and statistical strength of differential expression, with positive and negative scores corresponding to Low-DO- and High-DO-associated expression, respectively. GSEA was performed across the full ranked gene list for Gene Ontology Biological Process and Molecular Function categories. The minimum and maximum gene set sizes were set to 5 and 500, respectively. Adjusted *p* values were computed using the Benjamini–Hochberg method, and terms with adjusted *p* < 0.05 were considered significant. Top enriched GO Biological Process and Molecular Function terms were visualized as dot plots, with dot size proportional to gene count and color reflecting statistical significance.

### 2.9. RNA-Seq Read Processing, Mismatch Counting, Candidate Variant Calling, and Statistical Analysis

Raw paired-end WRS RNA-seq reads were processed with fastp v.0.20.1 [37] using automatic paired-end adapter detection (–detect_adapter_for_pe), a minimum base quality threshold of Q20 (–qualified_quality_phred 20), and a minimum post-trimming read length of 50 bp (–length_required 50). Trimmed reads were aligned to the *Saccharomyces cerevisiae* sacCer3 reference genome with STAR v.2.7 [32] using 32 threads. STAR was run in paired-end mode with gzip decompression enabled and coordinate-sorted BAM output. The alignment allowed up to 20 reported loci per multi-mapping read (–outFilterMultimapNmax 20) and used splice-junction parameters –alignSJoverhangMin 8 and –alignSJDBoverhangMin 1. Because of the compact structure of the yeast genome, intron and mate-gap limits were set to 20–2000 bp and 2000 bp, respectively (–alignIntronMin 20, –alignIntronMax 2000, –alignMatesGapMax 2000). Reads with a mismatch fraction greater than 0.04 of the read length were excluded during alignment (–outFilterMismatchNoverReadLmax 0.04). Alignment output was written as coordinate-sorted BAM files for downstream analysis.

STAR-aligned BAM files were preprocessed for RNA-seq variant calling using GATK v4.6.2 [35]. Read groups were added with AddOrReplaceReadGroups, duplicates were marked with MarkDuplicates, and spliced RNA-seq alignments were processed with SplitNCigarReads. BAM files were indexed after each step. GATK Mutect2 was then run independently for each WRS sample using the preprocessed split-N-CIGAR BAM files, with soft-clipped bases excluded from calling (–dont-use-soft-clipped-bases true). Raw calls were filtered with FilterMutectCalls using the corresponding Mutect2 statistics file. Downstream analyses were restricted to biallelic SNPs with PASS status and a tumor log-odds score (TLOD) > 6. The resulting per-sample filtered VCFs were indexed and merged into a non-redundant union set of variant alleles across samples, defined by chromosome, position, reference allele, and alternative allele. For each retained variant, the number of samples passing these filters was recorded, and retained positions were exported in BED format for downstream counting and recurrence analyses.

Callable genomic positions were identified for each preprocessed WRS BAM file using GATK CallableLoci. The callable-locus definition used a minimum depth of 10, minimum base quality of 10, minimum mapping quality of 10, maximum of one low-MAPQ read, maximum fraction of low-MAPQ reads of 0.1, and maximum depth of 1,000,000. Only intervals annotated as CALLABLE were retained. For each sample, read-level base counts were then extracted from these intervals using SAMtools mpileup with matching base-quality and mapping-quality thresholds (-Q 10, -q 10), exclusion of secondary and supplementary alignments (–ff 0x900), maximum depth of 1,000,000, and emission of all positions within callable intervals (-aa). Compressed per-sample mpileup files were used for mismatch counting and callable-site normalization.

Mismatch allele frequency (MAF) was calculated for each callable site after aggregating read counts across samples within each condition. For site i, substitution class m, and condition c, the MAF was defined as follows:

(1) $${MAF}_{i,c,m}=\;\frac{A_{i,c,m}}{R_{i,c,m}+A_{i,c,m}}$$

where A_i,c,m_ and R_i,c,m_ are the summed ALT- and REF-supporting read counts across samples in condition c, respectively. The Low-DO-associated change in MAF was calculated as follows:

(2) $${∆MAF}_{i,m}=\;{MAF}_{i,L,m}\;-{MAF}_{i,H,m}$$

For each substitution class, the mean site-level ΔMAF was tested against zero using a one-sample *t*-test.

The sample-level mismatch rate (MR) was calculated separately for each sample and substitution class as the total number of ALT-supporting observations divided by the total number of REF-plus ALT-supporting observations across all filtered callable sites. Differences in the MR between High and Low DO were modeled using a beta-binomial generalized linear mixed model (GLMM) implemented in glmmTMB v1.1.14 [29,30]. For each sample and substitution class, the response was specified as the number of ALT-supporting observations versus REF-supporting observations, and the formula is defined as follows:

(3) (ALT, REF) ~ cond · mut + (1 | replicate)

where ALT and REF denote the numbers of ALT- and REF-supporting observations, respectively; cond represents the dissolved oxygen condition (H or L); mut represents the substitution class; and replicate is included as a random intercept to account for the paired biological replicate structure. MR fold change was calculated for each substitution class as the ratio of the model-estimated MR in Low DO to the model-estimated MR in High DO, based on estimated marginal means obtained on the response scale using emmeans v2.0.3 [38].

The variant rate (VR) was analyzed using an analogous sample-level modeling strategy. For each sample and substitution class, the VR was defined as the number of Mutect2-called variant sites divided by the number of callable genomic positions for the corresponding substitution class. Differences in the VR between High and Low DO were modeled using a negative binomial GLMM implemented in glmmTMB v1.1.14 [29]. For each sample and substitution class, the response was specified as the number of called variants, with the log-transformed callable denominator included as an offset, and the model formula is defined as follows:

(4) $$n_{mut}\;\sim \;cond\;\cdot \;mut\;+offset(log(n_{callable}))\;+\;(1\;|\;replicate)$$

where n_mut_ denotes the number of called variants; n_callable_ denotes the number of callable genomic positions used as the denominator for that substitution class. VR fold change was calculated for each substitution class as the ratio of the model-estimated VR in Low DO to the model-estimated VR in High DO, based on estimated marginal means obtained on the response scale using emmeans v2.0.3 [38].

Within-condition recurrence of called variants was assessed separately for each substitution class and oxygen condition. For each variant, recurrence was defined as the number of biological replicate samples within a condition in which the variant was detected. Variants were grouped into three recurrence categories, corresponding to detection in one, two, or three samples within the same condition. For each substitution class, counts were summed across recurrence categories separately for High DO and Low DO, and fractions were calculated by dividing the number of variants in each category by the total number of variants of that substitution class detected in the corresponding condition. High-DO and Low-DO recurrence distributions were compared separately for each substitution class using a 2 × 3 contingency table, with conditions as rows and recurrence categories as columns. Pearson’s chi-square test was used for each substitution class, and *p* values were adjusted across the six substitution classes using the Holm method.

## 3. Results

### 3.1. Experimental Design and Data Quality

**Figure 1:**
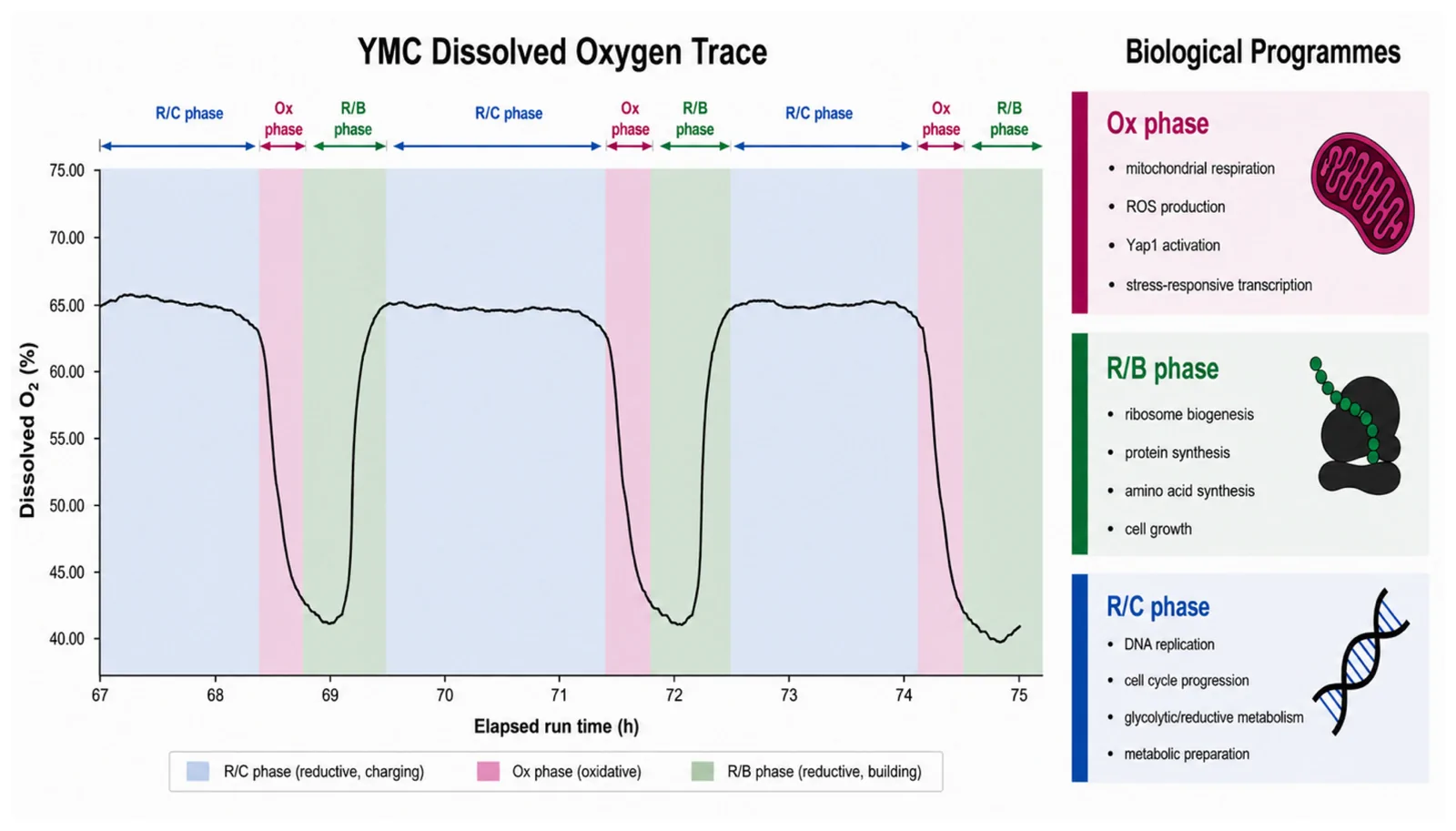
Schematic representation of temporal compartmentalization across the yeast metabolic cycle (YMC). The Ox, R/B, and R/C phase framework is conceptually based on the canonical YMC model described by Tu et al. [9]; all graphical elements were newly drawn by the authors. The dissolved oxygen trace is a representative schematic derived from the phase behavior observed in the present chemostat experiments and is used to illustrate sampling logic rather than to present the complete raw DO dataset. Low-DO WRS samples were collected near the dissolved oxygen nadir or on the descending DO slope, corresponding to the high-respiration phase, whereas High-DO WRS samples were collected near the dissolved oxygen peak, corresponding to the lower-respiration phase. The right panel summarizes canonical phase-associated biological programs and inferred redox burden based on the prior YMC literature. Intracellular ROS and specific nucleic acid lesions were not directly measured in the present study.

Yeast cells of strain DBY12007 were cultured in continuous glucose-limited chemostats and harvested at two defined YMC phases: Low DO, near the dissolved oxygen nadir or on the descending DO slope of the high-respiration phase, and High DO, near the dissolved oxygen peak of the lower-respiration phase, as illustrated schematically in Figure 1 and by raw DO traces in Supplementary Figure S1 [5,6,7,9]. Three biological replicates were collected per phase for ribosomal-RNA-depleted whole-transcriptome RNA sequencing (WRS), designated BRR1–BRR3_WRS1 for Low DO and BRR1–BRR3_WRS2 for High DO.

Periodic oscillations of dissolved oxygen confirmed synchronized respiratory cycling across cultures.

Reads were aligned to the standard S288C sacCer3 reference genome using STAR, with gene annotations derived from the S288C reference [32]. For the WRS dataset, each of the six samples yielded between 70 and 100 million uniquely mapped reads. Gene-level counts generated by featureCounts were used for differential expression analysis across 6607 annotated gene features, with low-count filtering applied as specified in the Materials and Methods [27,33]. Principal component analysis of variance-stabilized WRS counts separated Low-DO from High-DO samples along the first principal component, while biological replicates clustered tightly within each phase. This confirmed high reproducibility, clean phase assignment, and adequate data quality for transcriptomic and mismatch analyses.

### 3.2. Phase-Specific Transcriptome During the Yeast Metabolic Cycle

**Figure 2:**
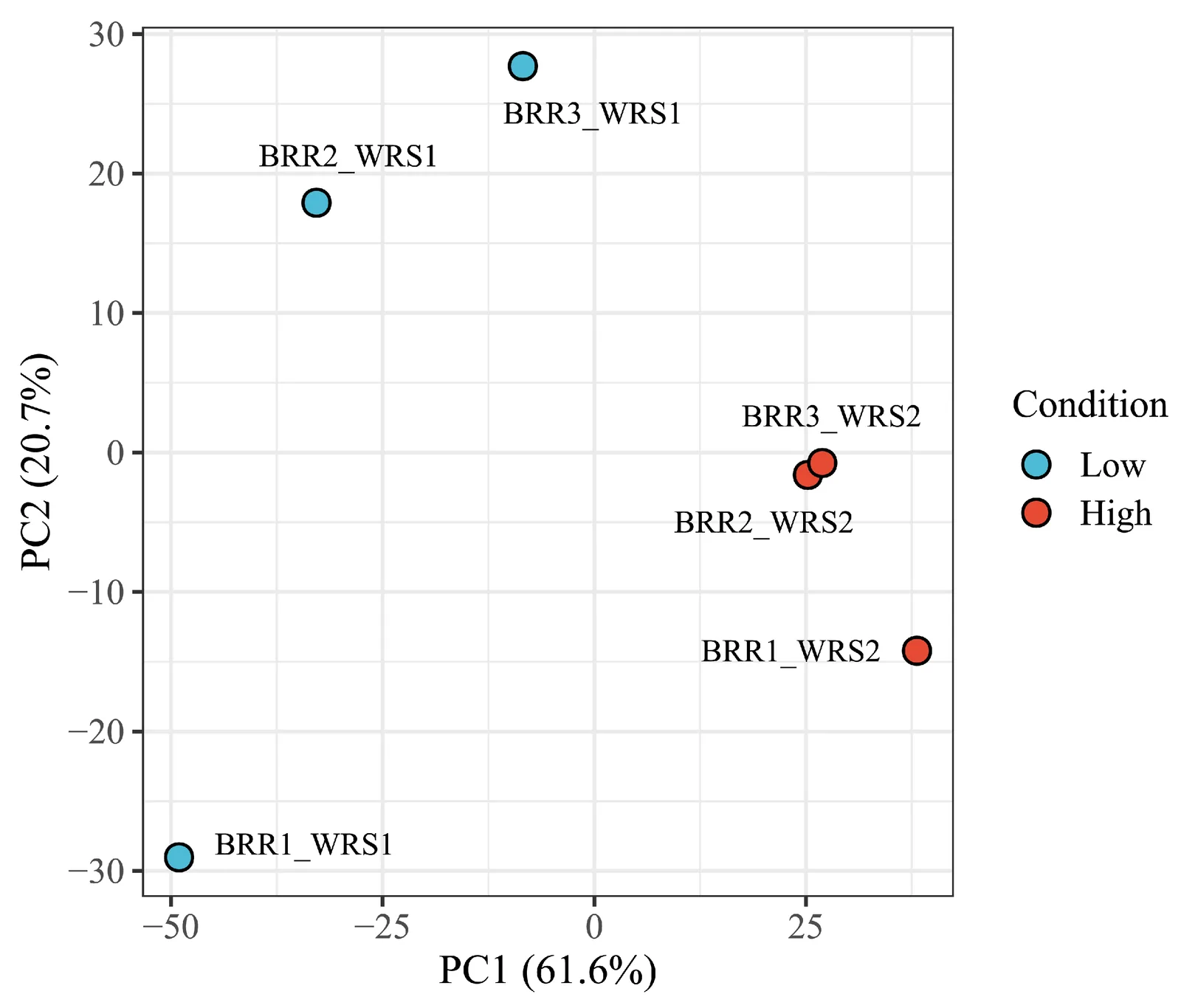
Principal component analysis of variance-stabilized RNA-seq count data. PCA was performed using variance-stabilized gene count data from Low-DO and High-DO samples.

PCA of variance-stabilized count data showed that PC1 accounted for a large proportion of the total variance (61.6%) and clearly separated the samples by experimental condition. Low-DO samples were positioned on the negative side of PC1, while High-DO samples were positioned on the positive side (Figure 2).

**Figure 3:**
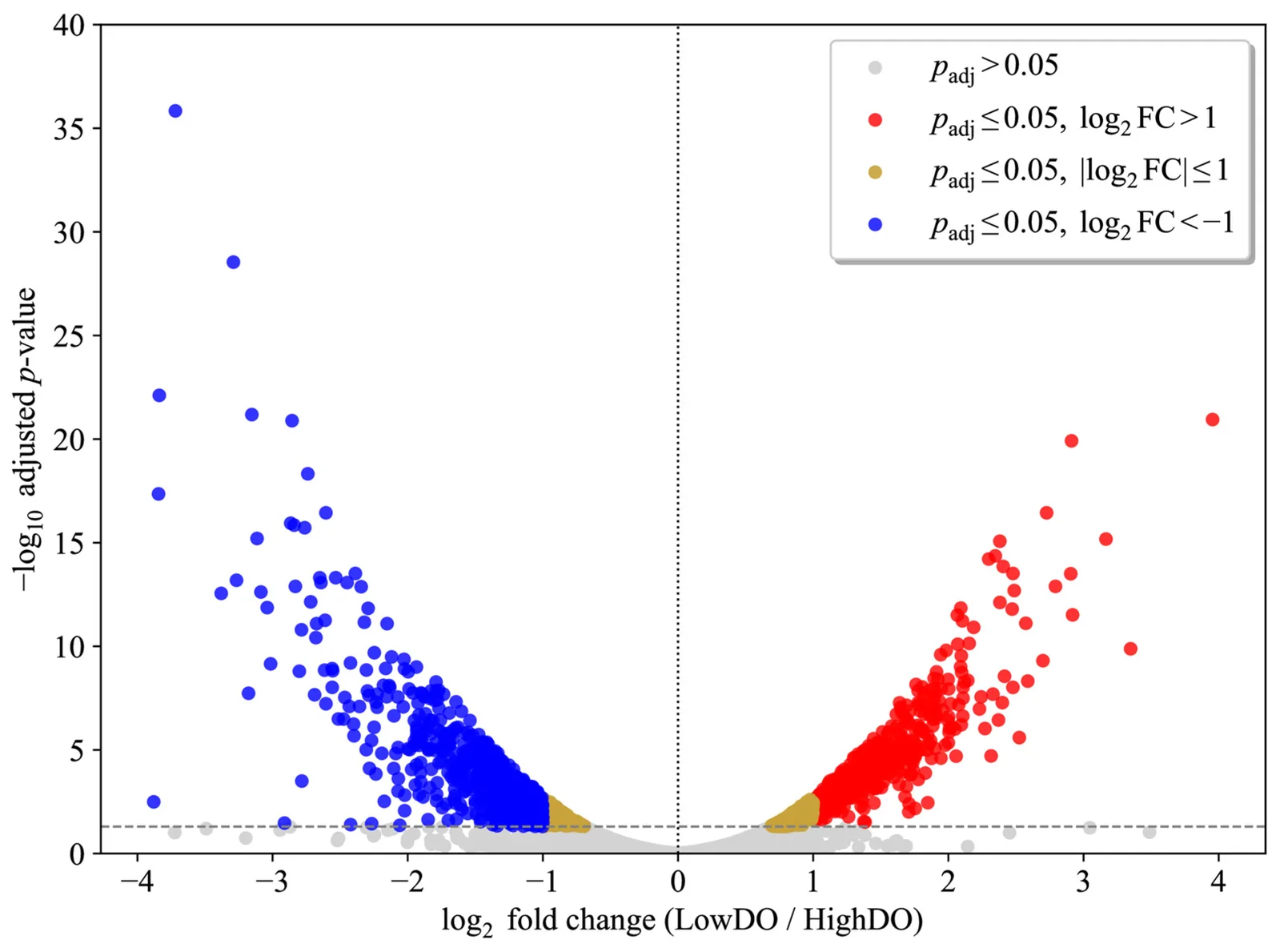
Volcano plot of differential expression between Low-DO and High-DO phases. Each point represents one gene; colored points indicate genes meeting both adjusted *p* < 0.05 and |log_2_FC| > 1. Positive log_2_FC indicates higher expression in Low DO; negative log_2_FC indicates higher expression in High DO. *n* = 6607 genes tested; 487 were Low-DO-enriched and 574 were High-DO-enriched at the stated thresholds.

Differential expression analysis between Low-DO and High-DO samples was performed using DESeq2 [27]. Of 6607 annotated gene features tested (Supplementary Table S1), 1505 showed statistically significant differential expression at adjusted *p* < 0.05. Applying an additional effect-size threshold of |log_2_FC| > 1 identified 487 genes that were more highly expressed in the Low-DO/high-respiration phase and 574 genes that were more highly expressed in the High-DO/lower-respiration phase (Figure 3). This threshold was used to focus downstream interpretation on genes showing at least a two-fold phase difference in either direction. Genes meeting both adjusted *p* < 0.05 and |log_2_FC| > 1 are provided in Supplementary Tables S2 and S3.

**Figure 4:**
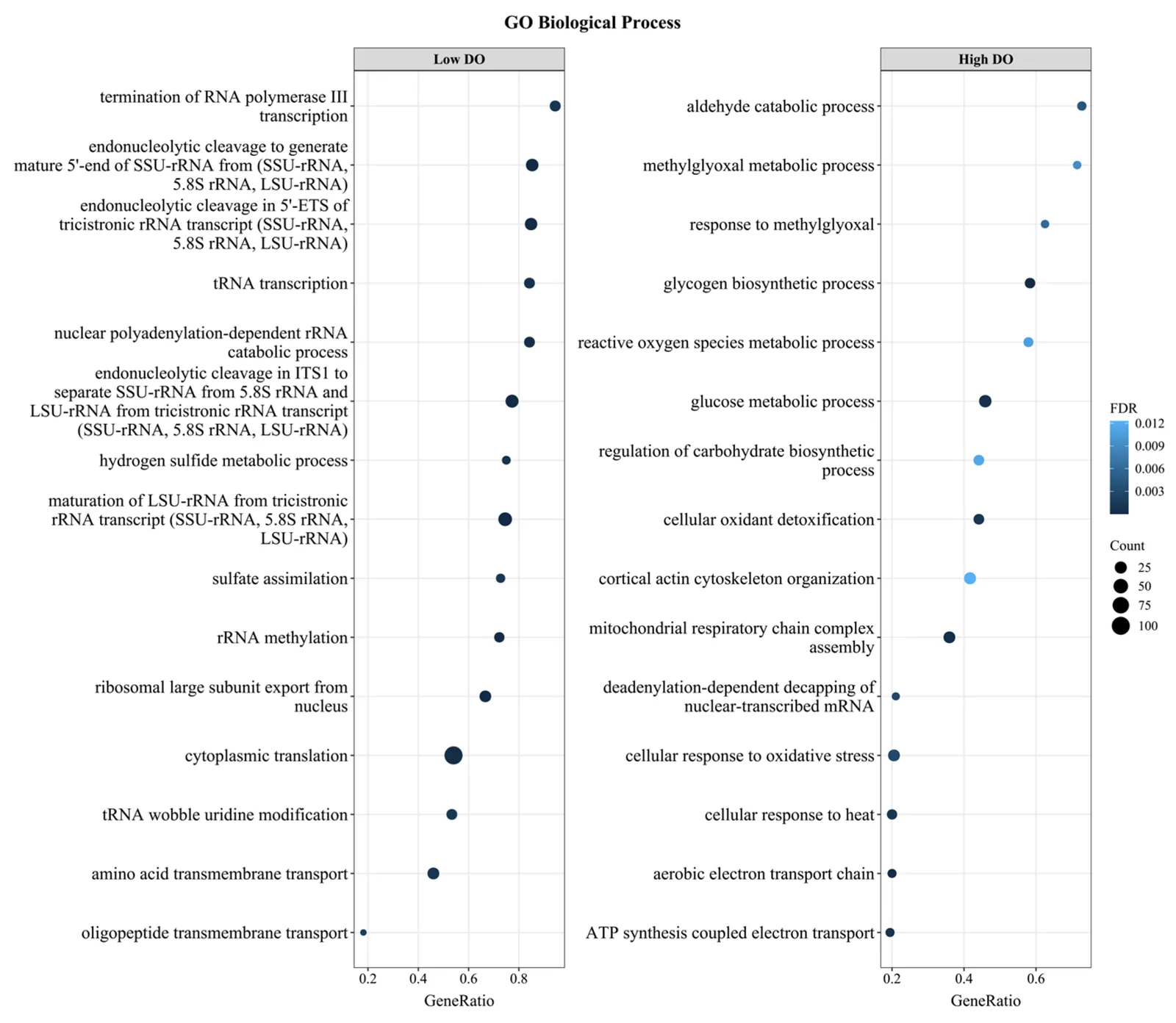
GO Biological Process gene set enrichment analysis (GSEA) dot plot based on genes ranked by differential expression. The top 15 GO Biological Process terms enriched at the high- and low-expression ends of the ranked gene list are shown separately. The *x*-axis represents the gene ratio, defined as the ratio of core enrichment genes to the total number of genes in a specific pathway; dot size indicates the number of genes assigned to each term and color denotes FDR significance.

**Figure 5:**
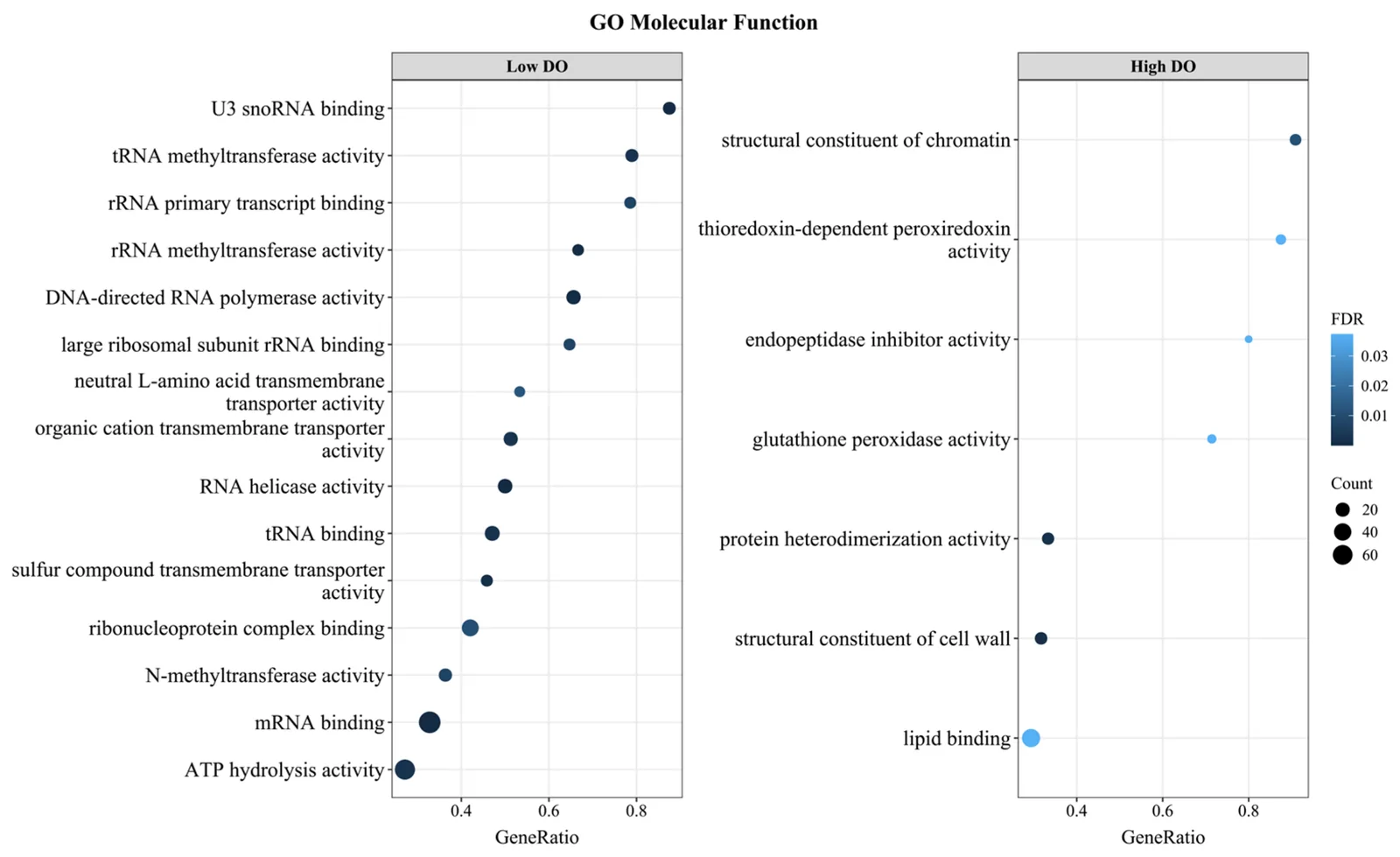
GO Molecular Function gene set enrichment analysis (GSEA) dot plot based on genes ranked by differential expression. The top enriched GO Molecular Function terms enriched at the high- and low-expression ends of the ranked gene list are shown separately, with seven terms shown for High DO and fifteen terms shown for Low DO. The *x*-axis represents the gene ratio, defined as the ratio of core enrichment genes to the total number of genes in a specific pathway; dot size indicates the number of genes assigned to each term, and color denotes FDR significance.

Gene Set Enrichment Analysis (GSEA) of differential expression between High-DO and Low-DO samples was performed using a pre-ranked gene list based on the direction and strength of differential expression. To minimize overlap among closely related GO categories, significant terms were additionally pruned by removing broad parental GO terms when more specific significant child terms were present in the same ontology. Therefore, the reported significant GO terms represent a non-redundant set of enriched categories, biased toward more specific biological or molecular functions rather than broad umbrella annotations. After this pruning step, 90 significantly enriched GO Biological Process terms and 29 GO Molecular Function terms remained; the top 15 Low-DO- and High-DO-enriched terms with the smallest FDR values are shown in Figure 4 and Figure 5 [28].

The pruned GO terms formed two functionally distinct phase-associated programs. The Low-DO gene set was enriched for RNA processing and maturation, including rRNA-related endonucleolytic cleavage, rRNA methylation, tRNA transcription and modification, as well as cytoplasmic translation, ribosomal large subunit export, hydrogen sulfide metabolic process, and amino acid or oligopeptide transport. This is consistent with the canonical Ox-phase logic of the YMC, in which high oxygen consumption supports growth-associated biosynthetic programs [8,9].

In contrast, the High-DO gene set was enriched for aldehyde and methylglyoxal metabolism, reactive oxygen species metabolism, cellular oxidant detoxification, cellular responses to oxidative stress and heat, carbohydrate and glycogen metabolism, mitochondrial respiratory chain complex assembly. This pattern suggests that detoxification, oxidoreductase, and redox-buffering-associated programs are prominent in the High-DO state and may reflect recovery and metabolic reorganization after the preceding high-respiration interval [3,4,5,6,7].

At the molecular function level, High-DO-enriched terms included thioredoxin-dependent peroxiredoxin activity and glutathione peroxidase activity, indicating transcriptomic enrichment of redox-buffering and oxidoreductase-related functions. In contrast, Low-DO-enriched molecular function terms were dominated by RNA-associated activities, including mRNA binding, RNA helicase activity, U3 snoRNA binding, rRNA primary transcript binding, large ribosomal subunit rRNA binding, tRNA binding, rRNA and tRNA methyltransferase activity, N-methyltransferase activity, ribonucleoprotein complex binding, DNA-directed RNA polymerase activity, and ATP hydrolysis activity. Low DO was also enriched for several transporter-related molecular functions, including sulfur compound, amino acid, organic cation, organic acid, organic anion, and oligopeptide transmembrane transporter activities. Together with the Low-DO-enriched biological process terms related to rRNA maturation, tRNA transcription and modification, cytoplasmic translation, and sulfur metabolism, these findings indicate that Low DO is enriched for RNA maturation, RNA remodeling, RNA modification, translational machinery, and associated transport functions [8,9]. In addition to the top NES-ranked terms shown in Figure 4, the fully pruned GO Biological Process results included Low DO enrichment of purine nucleobase metabolic process, indicating increased purine-base turnover, salvage, or interconversion during the Low-DO RNA-biogenesis phase (see Supplementary Table S4A, showing results with setSize < 188 for clarity).

We validated the phase assignment and expression architecture against independent YMC RNA-seq data from Douglas Murray and colleagues, who have extensively characterized the YMC in glucose-limited chemostats [3,4,5,6,9]. Re-analysis of the Murray dataset using the same phase definition (Low DO = trough/high-respiration phase; High DO = peak/lower-respiration phase) and a comparable analysis pipeline yields a high global correlation (approximately 0.78) between expression profiles. GO enrichment patterns were also concordant, with Low DO enriched for ribosome biogenesis and High DO enriched for antioxidant and stress-response functions. This agreement, despite differences in yeast strain and experimental details, supports the robustness and reproducibility of our differential expression analysis.

Mapping our phase-specific gene sets onto Murray/Tu phase categories further confirmed the phase assignments [3,4,9]. Among our 487 Low-DO-enriched genes, 229 mapped to Ox genes, 13 to R/B genes, and 10 to R/C genes. Conversely, among our 574 High-DO-enriched genes, 11 mapped to Ox genes, 63 to R/B genes, and 392 to R/C genes, as expected for genes peaking in reductive phases. Tu and McKnight illustrated phase-specific expression using periodic transcripts including *AAH1* (YNL141W; adenine deaminase), which peaks in the Ox phase [9]. Consistent with this assignment, *AAH1* was significantly enriched in Low DO (log_2_FC = 3.35, FDR = 1.32 × 10^−10^). *POX1* showed a nominal High-DO-associated trend (log_2_FC = −2.25, unadjusted *p* = 0.038), but it did not meet the FDR significance threshold (FDR = 0.125) and is therefore presented as a directional example rather than a statistically significant phase marker.

Representative, statistically significant genes from the ranked differential expression analysis are summarized in Supplementary Table S5. These include canonical YMC phase markers and high-ranking genes representing the principal Low-DO- and High-DO-enriched biological programs. The complete lists of genes contributing to the GO enrichment results are provided in Supplementary Table S4B.

### 3.3. Genome-Wide Base Substitution Analysis

Complementary substitutions were collapsed into the six canonical single-base substitution (SBS-6) classes using the standard pyrimidine-based representation: C > A, C > G, C > T, T > A, T > C, and T > G [31]. In this notation, complementary substitutions such as G > A and C > T are grouped into the same collapsed C > T class. To describe the orientation composition of the collapsed C > T class, the revised analysis also reports reference-oriented G > A and C > T components where available. The results are presented in two parts: mismatch-level analysis and variant-level analysis. Mismatches refer to stochastic read-level differences from the reference genome treated as individual base-level observations rather than stable site-level changes. Variants refer to differences supported strongly enough within a sample to be reported by a variant caller [34,35].

#### 3.3.1. Mismatch-Level Analysis

Mismatch features were calculated from callable genomic positions defined independently for each sample. Site-level changes were quantified using the mismatch allele frequency (MAF), defined for each substitution-specific locus as the fraction of reads supporting the alternative base after aggregating read counts across samples within each condition. Because callable masks were not identical across samples, the ΔMAF analysis was performed on the union of loci with valid allele-fraction estimates in both conditions. Thus, the approximately 4.13 million C-based and 6.67 million T-based loci represent analyzed substitution-specific loci rather than the callable-site count of any individual sample. Given the large number of loci, statistical testing was highly powered, and even small mean shifts could reach statistical significance. Therefore, interpretation focused primarily on the direction and magnitude of mean ΔMAF rather than on statistical significance alone.

**Figure 6:**
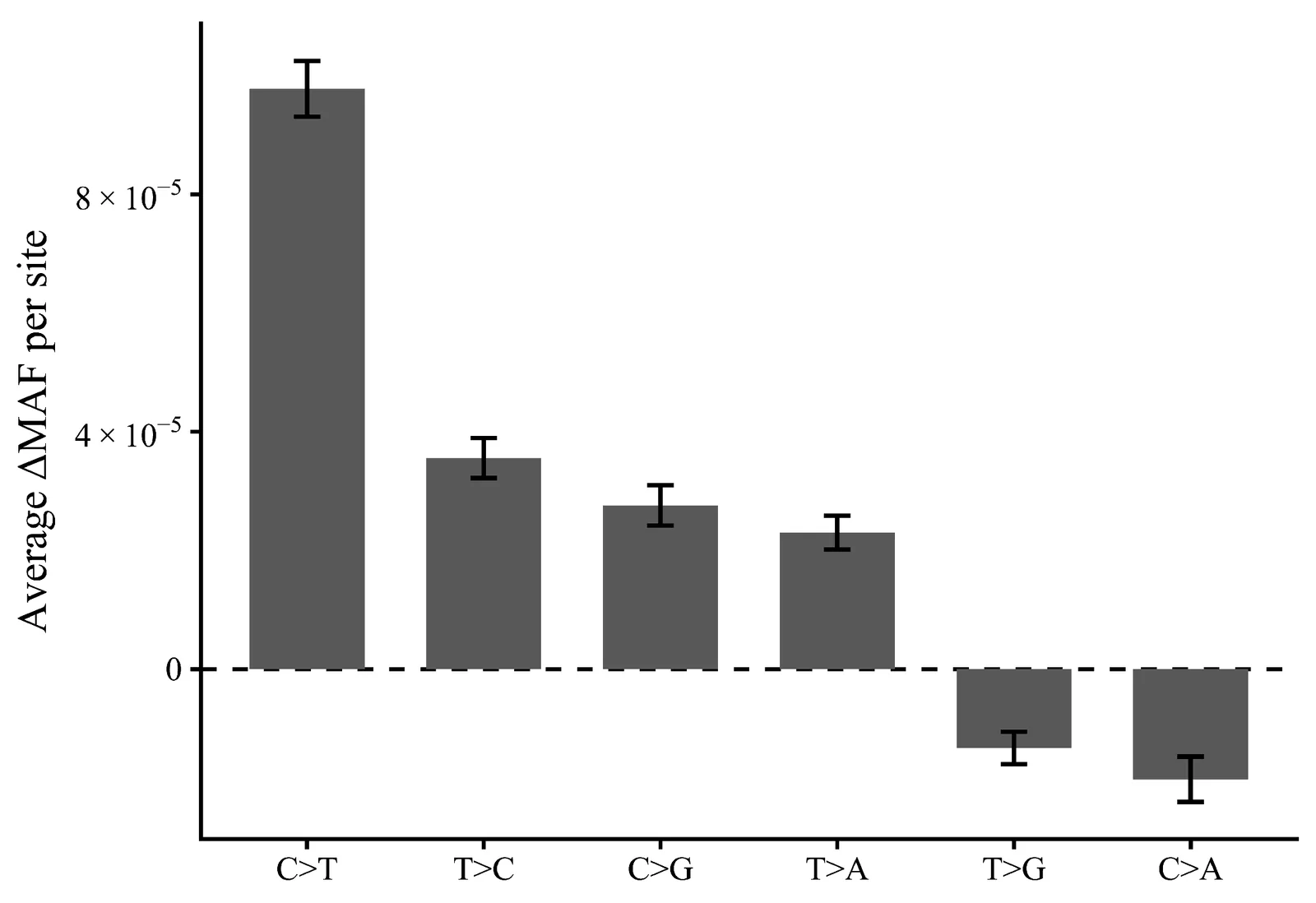
Average per-site change in mismatch allele frequency (MAF) across substitution classes. Positive values indicate higher MAF in Low DO relative to High DO, whereas negative values indicate lower MAF in Low DO. Bars show the mean per-site ΔMAF and error bars represent the 95% confidence interval of the mean estimated across callable sites. (See text for details.)

At the analyzed-locus level, the mean per-site ΔMAF differed from zero for all six substitution classes, but the magnitude and direction of these changes differed substantially by class (Figure 6). The collapsed C > T class showed the most pronounced positive shift, whereas C > A and T > G were the only SBS-6 classes that decreased. This indicates that the Low-DO-associated change was class-dependent rather than uniform across mismatch classes, with the strongest increase observed for C > T and opposite-direction shifts observed for C > A and T > G.

**Figure 7:**
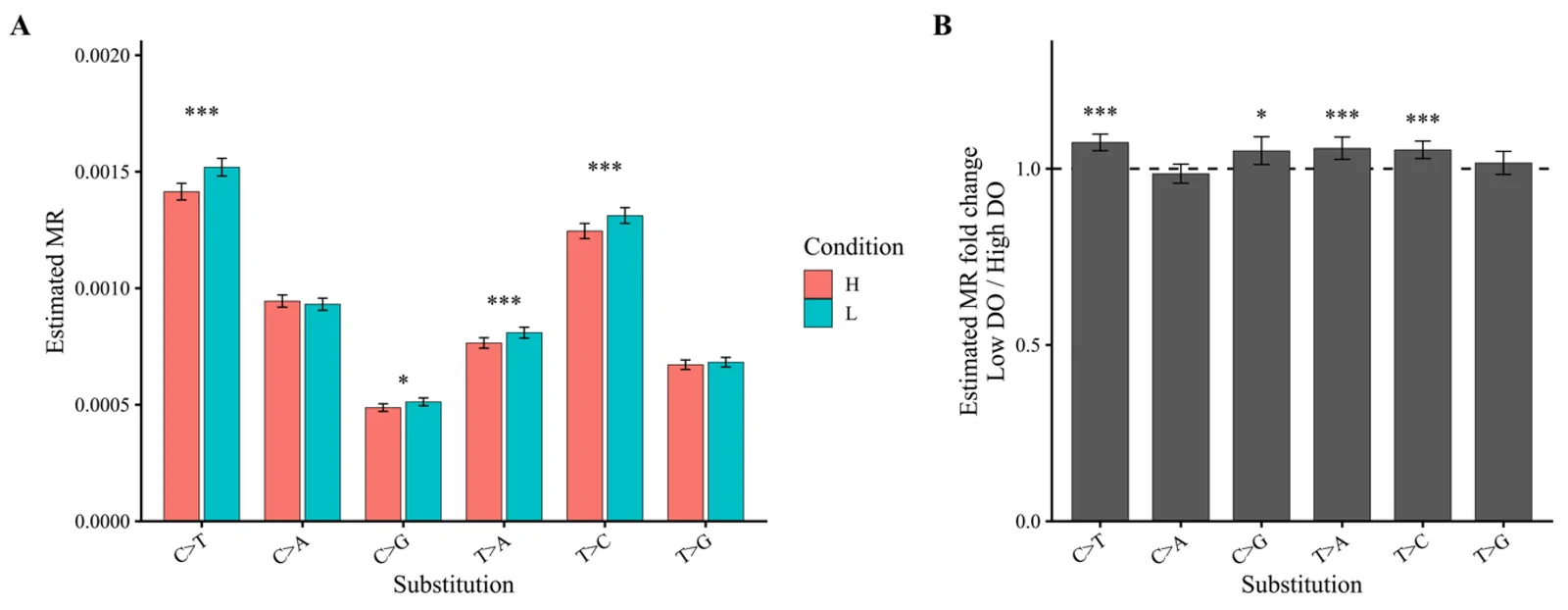
Model-estimated mismatch rate and Low-DO-associated fold changes across substitution classes. (**A**) Estimated mismatch rate (MR) for each substitution class in High DO and Low DO from a beta-binomial GLMM. (**B**) Estimated MR fold change between conditions, shown as Low DO/High DO. The dashed line indicates no change. Error bars indicate 95% confidence intervals estimated from the model. Stars indicate Holm-adjusted Low DO versus High DO post hoc contrasts within each substitution class: *p* < 0.05 (*), *p* < 0.001 (***).

Sample-level mismatch frequency was then quantified using the mismatch rate (MR), defined as the fraction of ALT-supporting mismatch observations among all REF- and ALT-supporting observations across callable positions. A beta-binomial GLMM was used to estimate the read-weighted MR for each substitution class in High DO and Low DO while accounting for biological replicate structure [29,30]. The model revealed a substitution-class-dependent MR response to Low DO (Figure 7). The collapsed C > T class showed the strongest Low-DO-associated increase, with an estimated MR fold change of 1.074 relative to High DO (MR Low DO = 1.519 × 10^−3^, MR High DO = 1.414 × 10^−3^; see Supplementary Table S6). Thus, the collapsed C > T/G > A-compatible class showed the largest Low-DO-associated mismatch-rate increase. Significant positive shifts were also observed for C > G (FC = 1.051), T > A (FC = 1.058), and T > C (FC = 1.053), whereas T > G and C > A showed a slight decrease.

Thus, the Low DO effect was not restricted exclusively to the C > T/G > A-compatible class. Rather, Low DO was associated with a broader mismatch-rate shift in which the collapsed C > T class represented the most pronounced increase. Together with the site-balanced ΔMAF analysis, these results demonstrate a class-dependent mismatch response during the High DO-to-Low DO transition, with the largest increase in the collapsed C > T/G > A-compatible class and additional changes in several other classes [12,15,31].

#### 3.3.2. Variant-Level Analysis

Variant-level changes were quantified using the variant rate (VR), defined as the number of Mutect2-called variant sites normalized by the number of callable genomic positions for the corresponding substitution class [35]. A negative binomial GLMM was used to estimate the VR for each substitution class in High DO and Low DO while accounting for biological replicate structure [29,30]. The model detected significant Low-DO-associated increases in several substitution classes, although the magnitude of these effects differed across classes.

**Figure 8:**
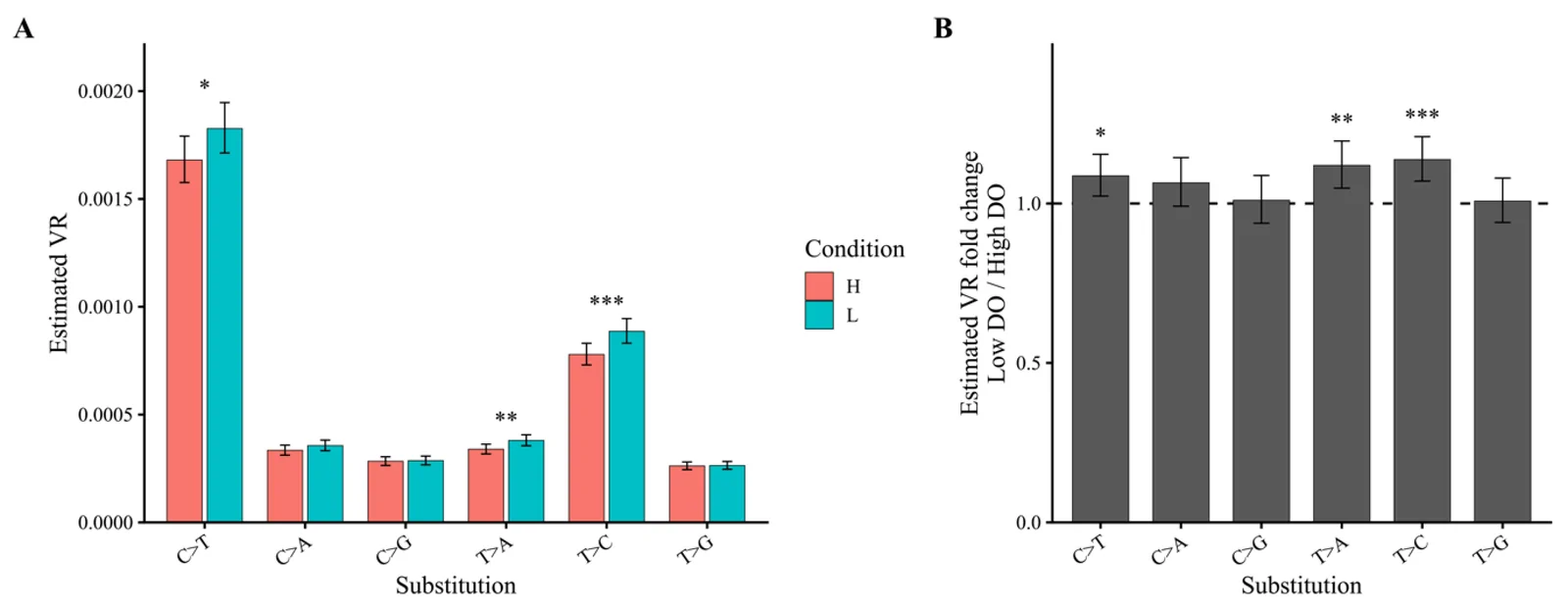
Model-estimated variant rates and Low-DO-associated fold changes across substitution classes. (**A**) Model-estimated variant rate (VR) for each substitution class in High DO and Low DO. (**B**) Estimated VR fold change between conditions, shown as Low DO/High DO. The dashed line indicates no change. In both panels, error bars indicate 95% confidence intervals estimated by the negative binomial GLMM. Stars indicate Holm-adjusted Low DO versus High DO post hoc contrasts within each substitution class: *p* < 0.05 (*), *p* < 0.01 (**), and *p* < 0.001 (***).

The collapsed C > T class showed a significant increase in Low DO, corresponding to an approximately 8.5% higher VR relative to High DO (VR Low DO = 1.826 × 10^−3^, VR High DO = 1.680 × 10^−3^; see Supplementary Table S7), consistent with the mismatch-level C > T/G > A-compatible trend. However, C > T was not the only substitution class affected at the variant level. The largest relative increases were observed for T > C and T > A, which increased by approximately 13.8% and 12.0%, respectively. In contrast, C > G and T > G showed little or no Low-DO-associated change, whereas C > A showed only a modest increase that did not reach statistical significance after post hoc testing (Figure 8).

Together, these results show that Low DO was associated with increases in several Mutect2-derived candidate-site classes rather than with an exclusively C > T/G > A-compatible response. The largest relative increases occurred in T > C/A > G and T > A/A > T, whereas the C > T/G > A-compatible class remained the most abundant. The molecular origins of these class-specific changes cannot be assigned from RNA-seq data alone because mismatch calls may reflect overlapping biological, library, alignment, and variant-calling processes [22,23,24].

We next examined whether called variants differed between High DO and Low DO in their marginal recurrence-degree distributions, defined as the number of samples within a condition carrying the variant. Across all substitution classes, variants detected in a single sample dominated, indicating that most called variants were sample-specific rather than recurrent across all three biological replicates. High-DO and Low-DO distributions were generally similar.

**Figure 9:**
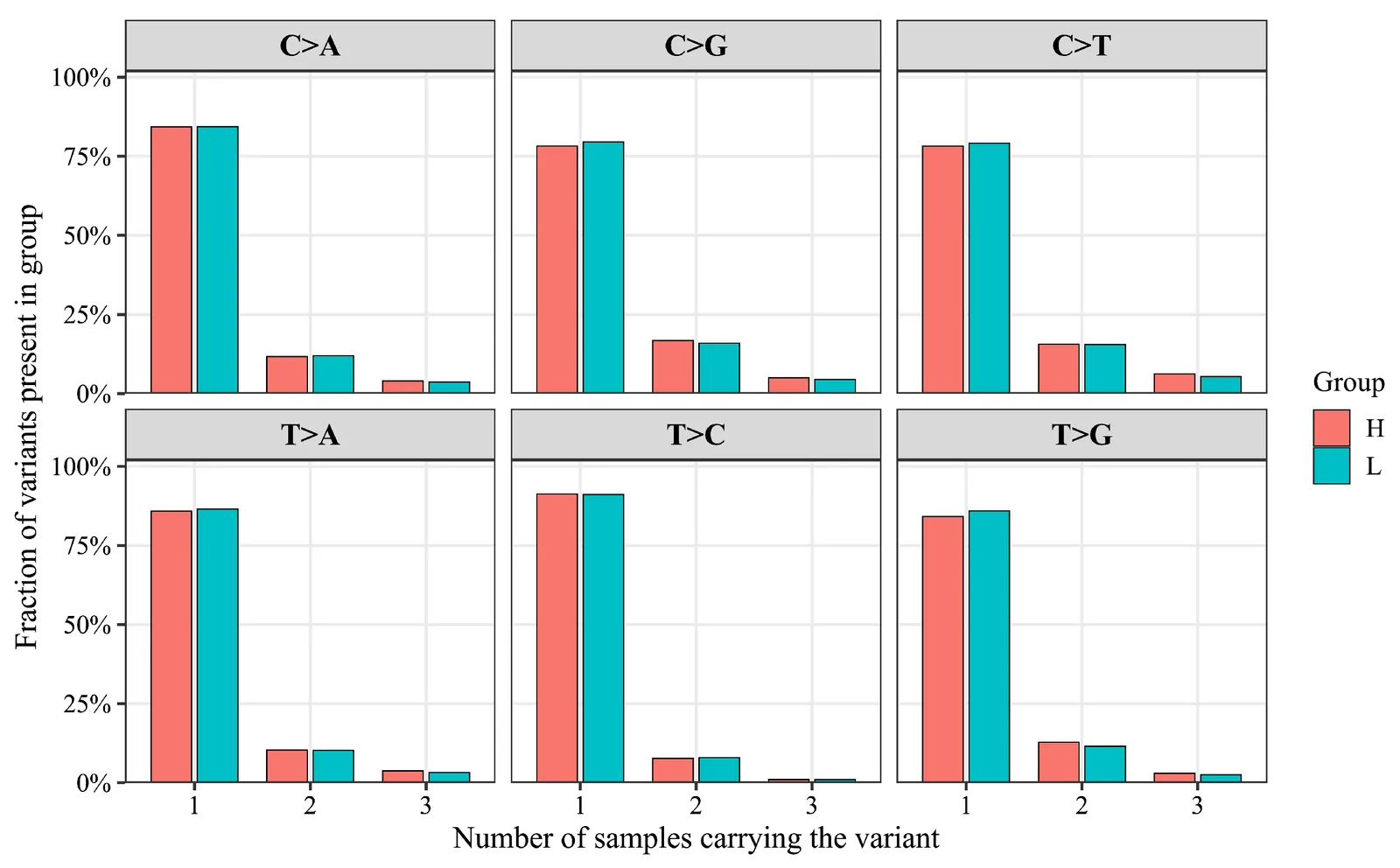
Marginal recurrence distributions of called variants in High- and Low-DO samples. For each substitution class, variants were grouped by the number of samples within a condition carrying the variant. Bars show the fraction of variants present in one, two, or three samples separately for High DO (H) and Low DO (L). Across all substitution classes, variants present in a single sample dominated, indicating that most called variants were sample-specific. High- and Low-DO distributions were broadly similar, with only minor changes in the fraction of single-sample variants between conditions (<2 percentage points).

Among the six substitution classes, C > T was the only class showing a statistically significant High DO–Low DO difference after multiple-testing correction, with a small shift in Low DO toward single-sample variants and away from variants present in all three samples. Specifically, the fraction of single-sample C > T variants increased from 78.2% in High DO to 79.1% in Low DO, whereas three-sample variants decreased from 6.2% to 5.4% (chi-square test, *p* = 0.0043; Holm-adjusted *p* = 0.0259). In comparison, T > G showed a larger absolute shift toward single-sample variants, increasing from 84.2% in High DO to 85.9% in Low DO, but this difference did not remain significant after Holm correction (*p* = 0.065; Holm-adjusted *p* = 0.327), as shown in Figure 9. This difference in statistical support is consistent with the approximately four-fold larger total number of C > T than T > G variant observations contributing to the recurrence test. Overall, condition-associated shifts in within-condition recurrence distributions were small, with only the C > T distribution supported after correction.

#### 3.3.3. Contrastive Pairwise Variant Analysis

A prior approach [39] used a contrastive case–control Mutect2 setup, which suppresses paired background calls but places the biological comparison inside an asymmetric tumor/normal (flight/ground) calling step. In contrast, our current YMC framework is more statistically explicit: Mutect2 was applied independently to each sample, and phase effects were estimated after calling using callable-site-normalized VR models and a biological replicate structure. This design therefore preserves the main advantage of the prior contrastive approach—control of shared background candidate sites—while avoiding its main limitation: dependence on an asymmetric caller configuration.

**Figure 10:**
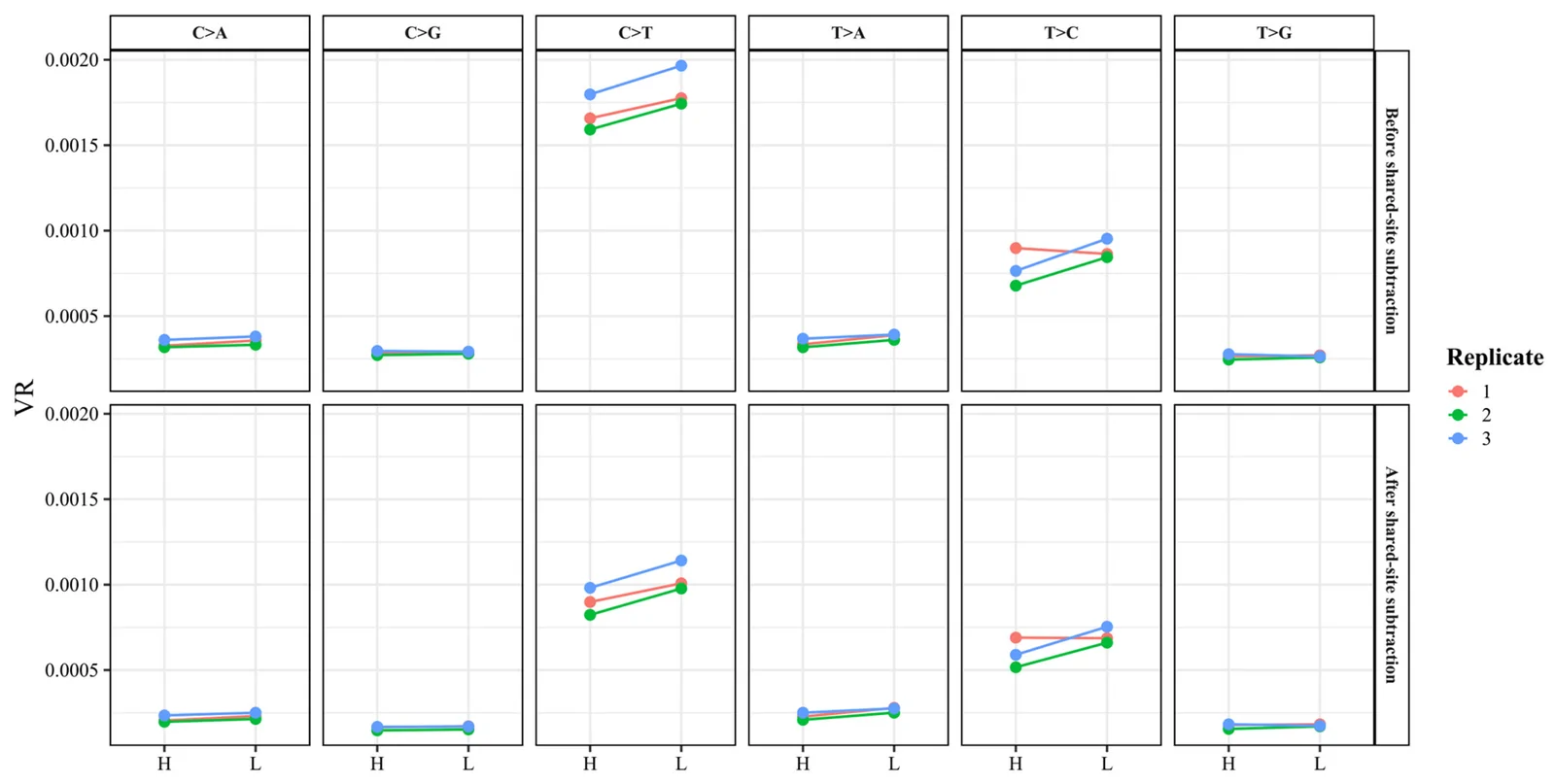
Effect of shared-site subtraction on SBS-6 candidate-site rates. Per-sample Mutect2-derived RNA-seq candidate-site rates are shown before and after removal of phase-shared candidate sites. Lines connect High-DO and Low-DO samples from the same biological replicate. Shared-site subtraction reduced overall candidate-site rates across SBS-6 classes but did not abolish the main Low-DO-associated pattern.

We then removed candidate alleles detected in both High-DO and Low-DO samples and recalculated per-sample SBS-6 candidate-site rates using the same callable denominators. This per-sample analysis showed the same qualitative pattern observed in the primary VR model, rather than only an average model-level effect, as shown in Figure 10 (see Supplementary Table S8). Removal of shared sites lowered the overall rates but did not eliminate the Low-DO-associated pattern across matched biological replicate pairs. The C > T/G > A-compatible class remained the dominant signal, with smaller retained Low-DO-associated increases in T > A and T > C. This supports the primary VR analysis and suggests that the observed pattern is not explained solely by candidate sites detected in both High-DO and Low-DO samples.

To describe the orientation composition of the collapsed C > T class, reference-oriented C > T and G > A events were analyzed separately. Each orientation accounted for approximately half of the class, and their relative proportions changed negligibly between Low-DO and High-DO conditions (see Supplementary Table S9). Although the large number of observations produced a formally significant chi-square test χ^2^ = 1.29 × 10^4^ (d.f. = 1, *p* ≈ 0), the effect size was negligible (Cramer’s V = 0.0009). Thus, the orientation balance of the collapsed class was effectively stable across conditions. This analysis does not identify the underlying lesion or establish a common chemical mechanism; accordingly, the class is referred to throughout as C > T/G > A-compatible.

## 4. Discussion

### 4.1. The YMC Separates Biosynthetic Growth from Redox-Buffering Programs

The WRS analysis confirms that Low DO and High DO represent functionally distinct metabolic states rather than simple differences in oxygen availability. Low DO, defined by the dissolved oxygen nadir, corresponds to maximal respiratory oxygen consumption and is enriched in ribosome biogenesis, rRNA processing, cytoplasmic translation, ribosomal subunit assembly, sulfur metabolism, and protein synthesis. High DO, defined by a dissolved oxygen peak, is enriched for detoxification, oxidoreductase activity, redox-buffering-associated pathways, and energy-generating pathways. This phase organization is consistent with the canonical YMC framework in which respiratory and biosynthetic programs are temporally coordinated with redox buffering and recovery [3,4,7,8,9].

The enrichment of detoxification and redox-buffering pathways in High DO should not be interpreted as evidence that High DO is more oxidatively damaging than Low DO. Rather, it likely reflects the timing of cellular recovery and redox buffering following the preceding high-respiration interval. In this interpretation, Low DO imposes a respiratory and redox burden, whereas High DO is enriched for programs that restore redox balance, detoxify reactive intermediates, and prepare the cell for subsequent metabolic transitions [3,4,5,6,7].

The concordance with Murray’s independent YMC dataset further supports the robustness of the phase assignment and the biological reproducibility of the expression architecture [3,4,5,6,9]. The strong agreement between our Low-DO-enriched genes and canonical Ox-phase genes, including *AAH1*, supports the interpretation that the WRS data capture authentic YMC phase biology rather than batch-specific transcriptional variation [9].

The present data do not test antioxidant interventions directly. Nevertheless, the enrichment of oxidant detoxification, peroxiredoxin, glutathione peroxidase, and oxidoreductase GO categories suggests that future YMC experiments could test whether natural or synthetic antioxidants, glutathione modulation, peroxiredoxin/thioredoxin perturbation, or catalase/SOD pathway manipulation alters the phase-dependent RNA-seq mismatch landscape. Such experiments would be required before concluding that redox buffering causally suppresses mismatch formation.

### 4.2. Low DO Is Associated with a Substitution-Class-Dependent RNA Mismatch Response

A useful interpretation separates the metabolic context from the measured RNA-seq readout. Low DO marks the high-respiration phase of the YMC, during which mitochondrial oxygen consumption is maximal and ROS pressure is inferred—but not directly measured—to be higher than during High DO. The measured downstream response is a mixed, substitution-class-dependent RNA-seq mismatch landscape.

The mismatch and variant-rate analyses indicate that the transition to Low DO is associated with a substitution-class-dependent RNA-seq mismatch response. The collapsed C > T class, which includes complementary G > A events in pyrimidine-based SBS-6 notation, showed the strongest Low-DO-associated increase at the mismatch level and was also significantly increased at the variant level [31]. This class is compatible with several non-exclusive processes, including cytosine deamination and related cytosine alterations [16,17,20], the persistence or repair of damaged bases [18], transcriptional mutagenesis at damaged templates [19], and metabolism-associated mixed substitution processes [21]. RNA-seq data cannot determine whether altered nucleotide pools, RNA modification, or reverse-transcription readout also contributed [22,23,24]. This interpretation should be considered mechanistically compatible rather than diagnostic, because RNA-seq-derived mismatches are not direct chemical measurements of lesion identity.

The accompanying T > C/A > G and T > A/A > T increases show that the Low-DO-associated response extends beyond one transition class. Their mechanisms remain uncertain. Possible contributors include transcript composition, RNA maturation and modification, nucleotide-pool state, reverse-transcription behavior, and library-dependent effects. Low DO enrichment of purine nucleobase metabolism provides a metabolic context for altered nucleotide turnover, but it does not demonstrate adenine deamination, RNA editing, or any particular mismatch-generating reaction. Importantly, in *S. cerevisiae*, this broader landscape should not be attributed primarily to canonical ADAR-type RNA editing. The absence of an extensive endogenous A-to-I editing system constrains a canonical RNA-editing explanation and shifts the mechanistic emphasis toward oxidative base chemistry, modified nucleotide pools, RNA maturation, RNA modification, transcript turnover, and reverse-transcription readout.

The T > A/A > T increase further argues against any purely transition-based or single-lesion explanation. Taken together, the C > T/G > A, T > C/A > G, and T > A/A > T changes define a mixed spectrum whose components may arise from overlapping biological and technical processes. The data therefore support a phase-associated transcriptome-fidelity response but do not permit assignment to 8-oxoG, cytosine deamination, carbonyl damage, or any other individual lesion.

The mixed spectrum is consistent with the redox-cycle resonance and RDD frameworks, both of which predict that the metabolic phase can alter the biochemical and processing environment of transcripts without producing a single lesion-specific signature [11,24]. Redox state may influence base chemistry, nucleotide pools, RNA maturation, modification, turnover, and reverse-transcription readout simultaneously. The central conclusion is therefore that metabolic phase alters the transcriptome-fidelity environment, not that one molecular lesion explains all observed substitutions.

The recurrence analysis further supports a stochastic or transient component of the Low-DO-associated variant landscape. Most called variants were sample-specific, rather than being present across all three biological replicates. Only the collapsed C > T class showed a statistically supported High DO–Low DO recurrence distribution shift after multiple-testing correction, and this shift was small in absolute magnitude. These findings argue against the interpretation that the observed variants represent stable, recurrent DNA-level sequence changes. Instead, they are more consistent with RNA-seq-derived mismatch events, stochastic damage, low-frequency transcriptional or post-transcriptional errors, or library-dependent readout of chemically altered RNA [12,14,24].

### 4.3. Potential Contributors to the Broader Mismatch Spectrum

The T > C/A > G and T > A/A > T increases indicate that the Low-DO-associated RNA-seq response is broader than the C > T/G > A-compatible class. Several observations provide possible context. Low DO was enriched for RNA processing, RNA modification, methyltransferase activity, pseudouridine synthesis, tRNA modification, rRNA maturation, and translation, whereas High DO was enriched for aldehyde and methylglyoxal metabolism, oxidant detoxification, peroxidase activity, and related redox-buffering functions. Methylglyoxal is a reactive dicarbonyl intermediate and potent inducer of advanced glycation end products [40]. In the present study, the enrichment of methylglyoxal-related pathways provides a carbonyl-stress context but does not establish that methylglyoxal-derived adducts caused any of the observed RNA-seq mismatch classes. 

These pathway differences suggest that RNA maturation state, modified nucleosides, nucleotide and carbonyl metabolism, transcript composition, and reverse-transcription behavior may contribute to the observed spectrum. Prior yeast studies also show that metabolism-associated and aldehyde-associated DNA mutagenesis can produce mixed substitution patterns [21,41]. However, those studies concern DNA mutagenesis and do not establish the origin of RNA-seq mismatches in the present experiment.

Accordingly, T > C/A > G and T > A/A > T should be treated as mechanistically unresolved components of a broader, phase-associated mismatch landscape. Direct RNA-modification mapping, oxidized-nucleoside quantification, matched DNA/RNA sequencing, and library-independent validation will be required to determine their origins [22,23,24].

### 4.4. Redox Phase, RNA Stability, and Transcriptome Fidelity

The YMC reveals how metabolic state can organize the timing of cellular risk. DNA replication is restricted to the reductive phase, presumably reducing the probability that replication forks encounter elevated respiratory oxidative stress [10]. Under the chemostat conditions used here, the dilution rate of D = 0.1 h^−1^ corresponds to an approximate population doubling time of ln(2)/D ≈ 6.9 h. Because the YMC period was approximately 90–100 min, roughly four to five metabolic cycles occur per cell division on average. Therefore, most phase-resolved WRS signals are expected to reflect transcriptional, post-transcriptional, RNA-level, or library-readout processes rather than newly fixed DNA replication mutations.

Transcription, however, remains active during the respiratory phase [8,9]. This creates a different form of vulnerability: damage need not be fixed into DNA to influence phenotype. Oxidatively damaged, chemically modified, or mistranscribed RNA can alter translation, protein quality, RNA decay, stress signaling, and downstream regulatory feedback [12,13,14,15,24].

The present data support a model in which the transcriptome carries a temporally structured record of the metabolic state. Low DO/high respiration is associated with a reproducible but mixed mismatch landscape, including a prominent C > T/G > A-compatible component and additional T > C/A > G and T > A/A > T changes [12,14]. At the same time, the prominence of T > A and T > C changes indicates that the mismatch landscape is mechanistically mixed and cannot be reduced to a single lesion type. The dominant substitution class is embedded within a broader RNA-seq mismatch landscape shaped by biological phase, transcript composition, RNA modification, reverse-transcription behavior, library chemistry, alignment, and computational filtering [12,13,14,15,22,23,24].

Although this manuscript does not directly measure RNA half-life or RNA decay, the phase-specific transcriptome suggests that RNA processing and transcript fate are central to the YMC. Low DO is enriched in RNA maturation, translation, and ribosome biogenesis programs, while High DO is enriched in antioxidant and detoxification pathways. This pairing suggests a model in which transcriptional and post-transcriptional demands are highest during the respiratory phase, while redox-buffering and detoxification programs become more prominent during the subsequent phase [3,4,5,6,7]. Future work should directly measure transcript half-lives and determine whether mismatch-bearing transcripts are preferentially degraded by Xrn1-dependent decay, the nuclear exosome, ribosome-associated quality control, or other RNA surveillance pathways [13,14,24].

### 4.5. Relationship to the Redox-Cycle Resonance Model

The present findings provide an RNA-seq-based extension of the redox-cycle resonance model proposed by Stolc, Shmygelska, and Griko [11] and align with the more recent RNA–DNA difference (RDD) framework linking oxidative stress, transcriptional fidelity, RNA editing, RNA damage, and sequence discordance [24]. The resonance model proposed that the timing of transcription relative to the cellular redox cycle creates non-random windows of sequence vulnerability: genes transcribed during more oxidizing phases encounter a different chemical and metabolic environment from genes transcribed during reductive phases. Vulnerability, therefore, depends not only on expression level but also on when transcripts are synthesized and processed relative to the redox cycle.

The present study does not test long-term adaptation, codon-bias evolution, or heritable DNA mutation. Instead, it examines a more immediate consequence of the same principle: whether RNA-seq mismatch and candidate variant-rate patterns differ across redox-defined metabolic phases. Low DO/high respiration was associated with a substitution-class-dependent response, with the largest mismatch-rate increase in the C > T/G > A-compatible class and additional T > A/A > T and T > C/A > G changes indicating a broader RNA-processing, RNA-modification, and sequencing-readout landscape. These findings support the view that the redox phase can structure transcriptome fidelity before stable DNA mutation or phenotypic adaptation is established.

The redox-cycle resonance framework predicts non-random vulnerability rather than deterministic mutation at every locus. The present data are consistent with that narrower prediction because the mismatch landscape is phase-dependent, substitution-class-dependent, and embedded within a Low-DO transcriptome enriched for RNA maturation, ribosome biogenesis, sulfur metabolism, and translation. Thus, the YMC creates a temporally organized window in which transcriptional demand, respiratory redox pressure, redox-buffering capacity, and RNA-processing state converge to shape RNA sequence-discordance burden. Transcriptome fidelity may therefore be an emergent property of timing: transcripts produced during high-respiration phases may experience different risks from those produced during lower-respiration, redox-buffering-enriched phases.

### 4.6. Conserved Metabolic Timing Cues from Yeast to Mammalian Biology

The translational relevance of the YMC is strengthened by conserved metabolic signals that link mitochondrial activity, redox state, and biological timing. Carbon monoxide (CO), produced during heme degradation, has been shown to advance the yeast metabolic cycle into its oxidative respiratory phase [42]. In mammals, heme and CO participate in circadian regulation. CO inhibits DNA binding by heme-loaded NPAS2–BMAL1 complexes [43], while REV-ERBα and REV-ERBβ bind heme and connect the metabolic state with circadian transcription [44].

These observations do not imply that the YMC is equivalent to mammalian circadian or sleep–wake physiology. Rather, they indicate that redox state, mitochondrial respiration, heme/CO signaling, and biological timing can be coupled across yeast and mammalian systems [5,6,7,42,43,44]. The YMC therefore provides a reductionist model for investigating how the metabolic phase may influence transcriptome fidelity in conditions where mitochondrial demand and redox-buffering capacity become mismatched, including circadian disruption, sleep deprivation, intermittent hypoxia, and hypoxia–reoxygenation [11,45].

Future comparative studies should test whether phase-dependent transcriptome-fidelity effects occur in mammalian circadian, sleep–wake, inflammatory, or hypoxia–reoxygenation models. Reports that *Saccharomyces* strains synthesize melatonin during growth and fermentation provide an additional comparative avenue, although melatonin was not examined in the present study [46].

### 4.7. Relevance to Sleep–Wake Redox Biology and Psychiatric Vulnerability

Sleep–wake regulation, circadian timing, and psychiatric vulnerability involve coordinated changes in mitochondrial activity, redox buffering, inflammatory tone, neuromodulatory stability, and biological timing [45]. The YMC provides a simplified eukaryotic system in which the respiratory phase, RNA-processing state, and transcriptome fidelity can be examined with temporal precision [3,4,5,6,7,9]. The present results suggest that transitions between metabolic states create windows in which RNA molecules may become differentially susceptible to sequence discordance, altered processing, and quality-control filtering.

Analogous vulnerability windows may occur in mammalian systems during sleep deprivation, circadian disruption, intermittent hypoxia, chronic stress, inflammation, or sustained neuromodulatory demand, when the mitochondrial load exceeds redox-buffering capacity [11,45]. Clinically, such states are associated with fatigue, cognitive inefficiency, hypersomnolence, insomnia, and mood dysregulation. These symptoms can precede structural neurodegeneration and may reflect a potentially reversible phase of cellular instability [45]. The present study does not establish that RNA mismatch burden causes psychiatric or sleep-related symptoms. Rather, it identifies a cellular principle that may be clinically relevant: metabolic timing can alter transcriptome fidelity before stable genomic damage or overt tissue injury occur [4,9,45].

Mitochondrial redox-buffering systems such as SOD2 represent plausible resilience nodes within this framework, although SOD2 was not measured in the present study. Genetic loss of SOD2 produces severe mitochondrial and organ-level pathology, supporting its role as a major defense against respiratory superoxide [47]. Failure of mitochondrial redox buffering could increase the probability that metabolic transitions produce RNA damage, mistranslation, inflammatory signaling, or maladaptive stress responses [14,45,47]. Future studies should test whether genetic or pharmacological perturbation of *SOD2* and related redox-buffering pathways alters phase-dependent RNA mismatch burden in yeast and whether comparable RNA sequence-discordance patterns occur in mammalian models of sleep loss, circadian disruption, intermittent hypoxia, or psychiatric vulnerability.

The YMC is, therefore, not proposed as a direct model of sleep or psychiatric disease. Its translational value lies in the conserved cellular logic by which biological timing, mitochondrial activity, redox-buffering capacity, RNA damage, and RNA quality control interact [9,13,14,15,24]. This framework provides a tractable basis for testing whether phase-resolved RNA sequence discordance can serve as an early indicator of declining cellular resilience in clinically relevant redox-stress states.

### 4.8. Limitations and Future Directions

Because intracellular ROS, antioxidant enzyme activity, glutathione redox state, and 8-oxoguanine abundance were not directly measured, the proposed Low DO → elevated ROS pressure → mixed transcriptome-fidelity response model should be interpreted as an inferred redox-state framework. Direct ROS measurement, oxidized-ribonucleoside quantification, matched DNA/RNA sequencing, and direct RNA sequencing will be required to assign specific chemical mechanisms.

RNA-seq-derived mismatches are not direct chemical measurements of any specific RNA or DNA lesion. The observed substitution classes may reflect overlapping contributions from transcriptional errors, RNA modification or damage, altered nucleotide pools, RNA-processing state, reverse-transcription behavior, mapping, library chemistry, and residual genomic variation. The C > T/G > A-compatible enrichment should therefore be described as redox-associated sequence discordance rather than as evidence of 8-oxoG, cytosine deamination, or another specific chemical reaction. Assigning molecular origin will require matched DNA/RNA sequencing, direct RNA sequencing, LC–MS/MS of modified or oxidized nucleosides, lesion-specific enrichment or derivatization, and replication using independent library methods.

A related limitation is that this study does not directly test the evolutionary or adaptive claims of the redox-cycle resonance model. The current data address phase-dependent RNA-seq mismatch burden over YMC states, not heritable DNA sequence change, codon-bias evolution, or long-term environmental adaptation. The redox-cycle resonance model is therefore used here as a conceptual framework for predicting phase-dependent, non-random transcriptome vulnerability, whereas the present evidence should be interpreted as RNA-seq-derived mismatch and variant-rate data requiring future chemical assays to identify the underlying lesions and functional assays to determine their biological consequences.

Second, the prominence of T-class substitutions in the variant-rate analysis requires careful control of library chemistry, transcript composition, and reverse-transcription behavior. Although oxidative-compatible C > T/G > A classes remain important, the global mismatch landscape is mechanistically mixed and cannot be reduced to a single lesion type. Future analyses should stratify mismatch spectra by transcript class, expression level, local sequence context, RNA modification status, and library preparation conditions [12,14,22,23,24].

The role of RNA editing should also be constrained by organismal context. In mammalian or immune-stimulated tissues, ADAR/APOBEC-mediated editing may contribute substantially to RNA sequence-discordance signals [24]. In *S. cerevisiae*, however, canonical ADAR-mediated A-to-I editing and RNAi-associated pathways are not expected to be major endogenous sources of the mismatch patterns observed here [26]. This makes canonical ADAR-mediated editing an unlikely major contributor in the present yeast system, but it does not distinguish among redox-associated chemistry, altered nucleotide pools, RNA modification, RNA-processing state, reverse-transcription behavior, and other technical readouts.

Third, the inference that RNA stability and RNA processing contribute to transcriptome-fidelity differences should be strengthened by direct decay modeling. The WRS phase-differential gene set and GO enrichment patterns support a major role for RNA maturation, processing, translation, and antioxidant recovery programs, but these data do not directly measure transcript half-lives. Future work should estimate RNA half-lives across YMC phases, measure decay kinetics for candidate transcripts, and test whether damaged transcripts are preferentially degraded by the nuclear exosome, Xrn1-dependent decay, or other RNA quality-control systems [13,14,24].

Fourth, variant-calling approaches were originally developed for DNA-seq and should be applied cautiously to RNA-seq data [35]. The Mutect2-derived VR analysis is useful for identifying strongly supported sample-level events, but it should not be interpreted as evidence for stable genomic mutation without matched DNA validation. The recurrence results, in which most called variants were sample-specific, are more consistent with an RNA-level, stochastic, or library-dependent interpretation than with stable inherited variation.

Finally, translational implications for sleep–wake regulation and psychiatric vulnerability should be treated as hypotheses generated from conserved cellular principles, not as direct clinical conclusions [45]. The strongest contribution of this manuscript is mechanistic: the metabolic phase is linked to temporally structured RNA mismatch landscapes in yeast. That mechanism provides a disciplined foundation for future translational studies in mammalian circadian, sleep, intermittent hypoxia, neuroinflammatory, and neuropsychiatric models.

## 5. Conclusions

This study demonstrates that the yeast metabolic cycle organizes both transcript abundance and RNA-seq sequence discordance across defined metabolic phases. Low DO/high respiration was characterized by enrichment in ribosome biogenesis, RNA processing and modification, translation, sulfur metabolism, and protein synthesis. In contrast, High DO/lower respiration was enriched for oxidant detoxification, oxidoreductase activity, and redox-buffering-associated pathways, consistent with temporal separation between biosynthetic demand and cellular recovery.

Mismatch-rate and candidate variant-rate analyses identified a substitution-class-dependent response associated with Low DO. The largest mismatch-rate increase occurred in the collapsed C > T/G > A-compatible class, while T > C/A > G and T > A/A > T also increased in the variant-rate analysis. The predominance of sample-specific candidate sites and the small magnitude of recurrence differences support a transient and heterogeneous RNA-seq mismatch landscape rather than widespread stable DNA mutation. Together, these findings indicate that the Low-DO-associated response is mechanistically mixed and cannot be assigned to 8-oxoguanine, cytosine deamination, RNA editing, or any other single lesion on the basis of RNA-seq data alone.

Taken together, these results support the central prediction of the redox-cycle resonance model [11] that sequence variation can arise from the metabolic state in which transcripts are synthesized and processed. The phase-dependent and substitution-class-dependent RNA-seq mismatch landscape indicates that metabolic stress is not merely a background condition but can structure the biochemical environment that gives rise to RNA–DNA difference (RDD)-compatible sequence variation [24]. In this framework, increased respiratory activity, altered redox balance, nucleotide-pool chemistry, RNA modification, and RNA-processing state converge to create non-random windows of transcriptional and post-transcriptional vulnerability. The findings therefore provide empirical support for a metabolic basis of RDD sequence discordance, in which cellular timing and redox state influence where and when RNA sequence variation becomes detectable.

The results therefore support the YMC as a reductionist eukaryotic model for studying how metabolic timing, respiratory state, redox buffering, RNA processing, and transcriptome fidelity interact. They further suggest that transcriptome fidelity depends not only on which genes are expressed, but also on the metabolic and redox environment in which their transcripts are synthesized and processed. Direct ROS measurements, matched DNA/RNA sequencing, chemical quantification of modified or oxidized nucleosides, direct RNA sequencing, and genetic or pharmacological perturbation of redox pathways will be required to determine the molecular origins and functional consequences of the observed mismatch patterns.

## Figures and Tables

**Table 1:** RNA concentrations and spectrophotometric purity ratios for WRS samples assessed by NanoDrop spectrophotometry.

Sample ID	YMC Phase	Concentration (ng/µL)	A260/A280	A260/A230
BRR1 WRS1	Low DO	33.34	2.383	2.173
BRR2 WRS1	Low DO	20.14	2.438	2.696
BRR3 WRS1	Low DO	26.33	2.347	2.015
BRR1 WRS2	High DO	22.43	2.369	2.191
BRR2 WRS2	High DO	21.09	2.365	1.463
BRR3 WRS2	High DO	15.65	2.581	2.77

WRS1 = Low-DO phase; WRS2 = High-DO phase; BRR1–BRR3 = biological replicates. NanoDrop A260/A280 and A260/A230 ratios assess spectrophotometric purity rather than RNA integrity. BRR2 WRS2 had an A260/A230 ratio of 1.463, slightly below the commonly used benchmark of 1.5.

## Data Availability

The original sequencing data presented in this study are openly available in the European Nucleotide Archive under accession PRJEB113244. Analysis scripts, processed results, and Supplementary Tables are openly available at https://github.com/bioinfobmcsas/ymc-redox-transcriptome (accessed on 16 July 2026). These files include raw count matrices, differential expression results, callable-site summaries, mismatch-rate and variant-rate tables, de-collapsed substitution spectra, recurrence analyses, and code required to reproduce the analyses. Key software versions include STAR 2.7.11b, featureCounts 2.1.1, SAMtools 1.18, BCFtools 1.18, GATK/Mutect2 4.6.2.0, R 4.5, DESeq2 1.46, clusterProfiler 4.14, glmmTMB 1.1.14, and ggplot2 4.0.3 [27,28,29,30,32,33,34,35,36,37,38], Saccharomyces Genome Database (SGD), Gene Association File (GAF) accessed 27 January 2026 (available from: https://current.geneontology.org/annotations/sgd.gaf.gz).

## Supplementary Materials

The following supporting information can be downloaded at: https://www.mdpi.com/article/10.3390/antiox15080914/s1: Figure S1. Dissolved oxygen raw oscillation traces and WRS sampling points. WRS1 samples were collected near the dissolved oxygen nadir or on the descending DO slope, corresponding to the high-respiration phase, whereas High DO WRS2 samples were collected near the dissolved oxygen peaks. Table S1: The DESeq2 results for the top 100 most significantly differentially expressed genes; Table S2: DESeq2 results for 487 Low-DO-enriched genes with adjusted *p* < 0.05 and log2FC > 1. Table S3: DESeq2 results for 574 High-DO-enriched genes with adjusted *p* < 0.05 and log2FC < -1. Table S4A: Pruned GO Biological Process results for enriched genes with setSize < 188 for clarity. Table S4B: Complete lists of genes contributing to the GO enrichment results. Table S5: Representative, statistically significant genes from the ranked differential expression analysis. Table S6: Model-estimated mismatch rates and Low-DO-associated fold changes across substitution classes. Table S7: Model-estimated variant rates and Low-DO-associated fold changes across substitution classes. Table S8: Per-sample Mutect2-derived RNA-seq candidate-site rates shown before and after removal of phase-shared candidate sites. Table S9: Composition of the de-collapsed C > T class to C > T and G > A events analyzed separately.
