# Supplementary material for: The Yeast Metabolic Cycle as a Tractable Cellular Framework for Redox Timing, Redox Buffering, and Transcriptome Fidelity
Source: Antioxidants (Basel). 2026 Jul 23;15(8):914. doi: 10.3390/antiox15080914 (PMC13510017; doi:10.3390/antiox15080914)
Supplement: Supplementary file 1 [file antioxidants-15-00914-s001.zip › antioxidants-4360521-supplementary.pdf]

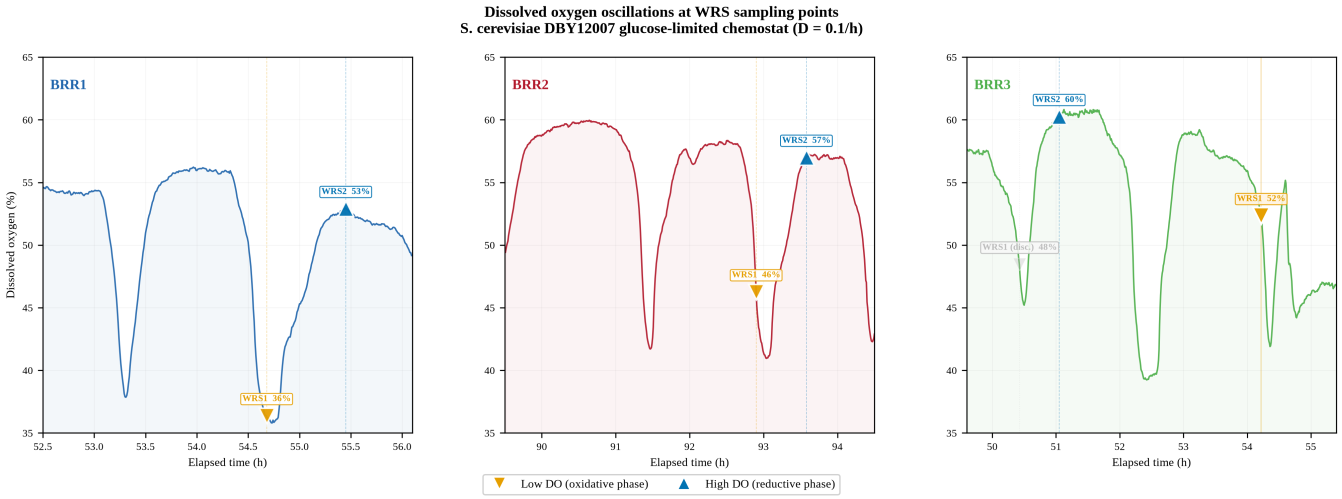

Figure S1. Dissolved oxygen raw oscillation traces and WRS sampling points. WRS1 samples were collected near the dissolved oxygen nadir or on the descending DO slope, corresponding to the high-respiration phase, whereas High DO WRS2 samples were collected near the dissolved oxygen peaks.

Supplementary Table S1.

The DESeq2 results for the top 100 most significantly differentially expressed genes

ORF	baseMean	log2FC	lfcSE	stat	pvalue	padj	gene
YNR034W-A	50158.95	-3.719	0.280	-13.302	2.26E-40	1.45E-36	EGO4
YHR087W	11222.68	-3.290	0.276	-11.923	8.98E-33	2.88E-29	RTC3
YGR248W	7029.34	-3.838	0.363	-10.581	3.63E-26	7.77E-23	SOL4
YDR224C	32020.49	-3.153	0.305	-10.352	4.10E-25	6.58E-22	HTB1
YNL142W	40186.18	3.955	0.385	10.278	8.87E-25	1.14E-21	MEP2
YMR169C	22544.40	-2.856	0.279	-10.248	1.20E-24	1.29E-21	ALD3
YGR055W	15105.73	2.911	0.291	10.014	1.33E-23	1.22E-20	MUP1
YCL048W-A	2872.01	-2.739	0.284	-9.631	5.91E-22	4.74E-19	YCL048W-A
YGR236C	12626.74	-3.844	0.409	-9.386	6.21E-21	4.42E-18	SPG1
YKL001C	26100.64	2.727	0.298	9.141	6.17E-20	3.60E-17	MET14
YKL026C	5996.00	-2.605	0.285	-9.144	6.02E-20	3.60E-17	GPX1
YNL195C	4949.31	-2.866	0.318	-9.005	2.15E-19	1.15E-16	YNL195C
YLR164W	6278.03	-2.840	0.317	-8.973	2.88E-19	1.42E-16	SHH4
YMR105C	30322.73	-2.762	0.309	-8.933	4.13E-19	1.89E-16	PGM2
YDR070C	78652.46	-3.115	0.354	-8.793	1.46E-18	6.24E-16	FMP16
YKL068W-A	10162.47	3.166	0.361	8.778	1.66E-18	6.67E-16	YKL068W-A
YDR253C	5242.47	2.381	0.272	8.744	2.24E-18	8.46E-16	MET32
YKR069W	11222.48	2.347	0.274	8.552	1.21E-17	4.32E-15	MET1
YPR167C	11819.05	2.299	0.270	8.505	1.82E-17	6.15E-15	MET16
YLL061W	19893.68	2.406	0.286	8.402	4.37E-17	1.40E-14	MMP1
YBR294W	76654.88	2.478	0.298	8.305	9.97E-17	3.05E-14	SUL1
YKL086W	1069.32	-2.387	0.288	-8.299	1.05E-16	3.05E-14	SRX1
YLR180W	12466.75	2.905	0.350	8.291	1.13E-16	3.14E-14	SAM1
YGR008C	48012.88	-2.533	0.308	-8.233	1.82E-16	4.87E-14	STF2
YOR173W	16351.52	-2.651	0.322	-8.227	1.92E-16	4.93E-14	DCS2
YFR017C	2737.92	-3.267	0.399	-8.189	2.63E-16	6.49E-14	IGD1
YDR171W	28854.63	-2.642	0.324	-8.149	3.67E-16	8.41E-14	HSP42
YER150W	2633.18	-2.449	0.300	-8.152	3.57E-16	8.41E-14	SPI1
YJL212C	1556.85	2.792	0.345	8.093	5.80E-16	1.28E-13	OPT1
YPR036W-A	51397.00	-2.831	0.350	-8.090	5.99E-16	1.28E-13	SPO24
YDL222C	11251.47	-2.344	0.290	-8.081	6.44E-16	1.33E-13	FMP45
YAL067C	2902.52	2.487	0.310	8.025	1.02E-15	2.04E-13	SEO1
YDL204W	28655.62	-3.086	0.386	-8.001	1.23E-15	2.39E-13	RTN2
YML100W	21830.37	-3.380	0.424	-7.979	1.48E-15	2.79E-13	TSL1
YMR174C	45802.52	-2.718	0.346	-7.858	3.91E-15	7.16E-13	PAI3
YJR010W	14497.93	2.382	0.304	7.846	4.28E-15	7.63E-13	MET3
YKL096W	789.83	-3.040	0.391	-7.770	7.83E-15	1.36E-12	CWP1
YJL109C	14081.72	2.092	0.270	7.760	8.47E-15	1.43E-12	UTP10
YJL161W	2854.54	-2.293	0.296	-7.753	8.95E-15	1.47E-12	FMP33
YGL184C	10105.66	2.472	0.319	7.739	9.98E-15	1.60E-12	STR3
YHR197W	6945.80	2.919	0.381	7.654	1.94E-14	3.04E-12	RIX1
YNL277W	20419.19	2.066	0.270	7.648	2.04E-14	3.11E-12	MET2
YER053C	3529.85	-2.611	0.345	-7.570	3.72E-14	5.55E-12	PIC2
YJR137C	49917.01	2.104	0.278	7.559	4.06E-14	5.92E-12	MET5
YEL039C	2614.88	-2.321	0.308	-7.536	4.84E-14	6.89E-12	CYC7
YDR021W	2082.57	2.573	0.342	7.518	5.56E-14	7.75E-12	FAL1
YIL100C-A	242.05	-2.674	0.356	-7.508	5.99E-14	8.06E-12	YIL100C-A
YJL144W	4542.16	-2.153	0.287	-7.507	6.03E-14	8.06E-12	ROQ1
YNL191W	4956.97	2.187	0.294	7.450	9.31E-14	1.22E-11	DUG3
YFR052C-A	7946.16	-2.786	0.376	-7.412	1.24E-13	1.59E-11	YFR052C-A
YPR160W	32779.26	-2.679	0.367	-7.293	3.03E-13	3.81E-11	GPH1
YFR030W	37005.77	2.154	0.299	7.203	5.88E-13	7.26E-11	MET10
YCL054W	15584.52	2.069	0.288	7.188	6.58E-13	7.96E-11	SPB1

YNL141W	9942.61	3.348	0.471	7.116	1.11E-12	1.32E-10	AAH1
YDL059C	5456.25	1.984	0.280	7.089	1.35E-12	1.58E-10	RAD59
YFL013W-A	4377.77	-2.247	0.319	-7.051	1.78E-12	2.03E-10	YFL013W-A
YIL074C	19029.25	1.944	0.277	7.018	2.26E-12	2.54E-10	SER33
YPL012W	20592.32	2.096	0.299	6.999	2.57E-12	2.84E-10	RRP12
YFL014W	426724.52	-2.119	0.304	-6.978	2.99E-12	3.25E-10	HSP12
YHR104W	10002.01	-2.028	0.292	-6.938	3.99E-12	4.27E-10	GRE3
YLL055W	11274.19	2.700	0.390	6.914	4.70E-12	4.94E-10	YCT1
YFR015C	7702.97	-2.423	0.352	-6.876	6.14E-12	6.35E-10	GSY1
YER054C	14339.58	-3.014	0.439	-6.860	6.91E-12	7.04E-10	GIP2
YER091C	49902.39	2.091	0.307	6.809	9.80E-12	9.83E-10	MET6
YML128C	46776.68	-1.936	0.284	-6.804	1.02E-11	1.00E-09	MSC1
YBR285W	3276.14	-2.163	0.319	-6.780	1.20E-11	1.17E-09	HAB1
YBL065W	162.77	-2.559	0.378	-6.775	1.24E-11	1.19E-09	YBL065W
YGR174W-A	3764.77	-2.024	0.299	-6.770	1.29E-11	1.21E-09	YGR174W-A
YER121W	7203.47	-2.306	0.342	-6.748	1.50E-11	1.39E-09	YER121W
YBR116C	2317.38	-2.615	0.388	-6.743	1.55E-11	1.42E-09	YBR116C
YLR178C	5807.49	-2.555	0.380	-6.733	1.66E-11	1.50E-09	TFS1
YOR185C	5143.65	-2.802	0.417	-6.722	1.79E-11	1.60E-09	GSP2
YKL035W	16222.48	-1.997	0.297	-6.712	1.93E-11	1.69E-09	UGP1
YLR223C	10503.79	1.913	0.285	6.708	1.98E-11	1.71E-09	IFH1
YMR229C	44440.72	2.099	0.313	6.696	2.15E-11	1.84E-09	RRP5
YCR087C-A	6288.92	2.416	0.364	6.632	3.31E-11	2.79E-09	YCR087C-A
YLR092W	16357.59	1.895	0.287	6.595	4.25E-11	3.54E-09	SUL2
YEL040W	2062.31	1.922	0.292	6.578	4.78E-11	3.93E-09	UTR2
YPL217C	23867.68	1.999	0.304	6.574	4.91E-11	3.99E-09	BMS1
YLR364W	2752.09	2.144	0.327	6.555	5.55E-11	4.45E-09	GRX8
YKL078W	3479.60	2.587	0.395	6.543	6.04E-11	4.78E-09	DHR2
YGR145W	11937.54	2.119	0.325	6.524	6.83E-11	5.28E-09	ENP2
YMR090W	8856.71	-1.791	0.274	-6.525	6.80E-11	5.28E-09	YMR090W
YDR060W	17539.30	1.761	0.272	6.481	9.10E-11	6.95E-09	MAK21
YNL194C	6043.70	-2.179	0.337	-6.466	1.01E-10	7.60E-09	YNL194C
YMR250W	15602.06	-2.137	0.331	-6.459	1.05E-10	7.85E-09	GAD1
YOR337W	6253.63	1.807	0.281	6.434	1.24E-10	9.17E-09	TEA1
YGR245C	11498.83	2.108	0.328	6.427	1.30E-10	9.48E-09	SDA1
YBL039C	13919.54	2.478	0.386	6.423	1.34E-10	9.56E-09	URA7
YLR390W	798.77	-2.132	0.332	-6.421	1.36E-10	9.56E-09	ECM19
YMR118C	3831.36	-2.559	0.399	-6.421	1.36E-10	9.56E-09	SHH3
YML093W	18970.96	2.002	0.313	6.393	1.62E-10	1.13E-08	UTP14
YBL004W	33784.67	1.894	0.296	6.389	1.67E-10	1.15E-08	UTP20
YKL014C	16547.21	1.947	0.305	6.386	1.70E-10	1.15E-08	URB1
YMR298W	2164.45	-1.990	0.312	-6.387	1.69E-10	1.15E-08	LIP1
YNL182C	3978.85	1.862	0.293	6.366	1.95E-10	1.30E-08	IPI3
YDR453C	2897.82	-1.770	0.278	-6.361	2.00E-10	1.32E-08	TSA2
YDL110C	30611.66	-1.789	0.281	-6.356	2.07E-10	1.36E-08	TMA17
YDL031W	8598.75	1.776	0.280	6.350	2.16E-10	1.40E-08	DBP10

Supplementary Table S2.
DESeq2 results for 487 Low-DO-enriched genes with adjusted p < 0.05 and log2FC > 1.

ORF	baseMean	log2FC	lfcSE	stat	pvalue	padj	gene
YNL142W	40186.18	3.955	0.385	10.278	8.87E-25	1.14E-21	MEP2
YGR055W	15105.73	2.911	0.291	10.014	1.33E-23	1.22E-20	MUP1
YKL001C	26100.64	2.727	0.298	9.141	6.17E-20	3.60E-17	MET14
YKL068W-A	10162.47	3.166	0.361	8.778	1.66E-18	6.67E-16	YKL068W-A
YDR253C	5242.47	2.381	0.272	8.744	2.24E-18	8.46E-16	MET32
YKR069W	11222.48	2.347	0.274	8.552	1.21E-17	4.32E-15	MET1
YPR167C	11819.05	2.299	0.270	8.505	1.82E-17	6.15E-15	MET16
YLL061W	19893.68	2.406	0.286	8.402	4.37E-17	1.40E-14	MMP1
YBR294W	76654.88	2.478	0.298	8.305	9.97E-17	3.05E-14	SUL1
YLR180W	12466.75	2.905	0.350	8.291	1.13E-16	3.14E-14	SAM1
YJL212C	1556.85	2.792	0.345	8.093	5.80E-16	1.28E-13	OPT1
YAL067C	2902.52	2.487	0.310	8.025	1.02E-15	2.04E-13	SEO1
YJR010W	14497.93	2.382	0.304	7.846	4.28E-15	7.63E-13	MET3
YJL109C	14081.72	2.092	0.270	7.760	8.47E-15	1.43E-12	UTP10
YGL184C	10105.66	2.472	0.319	7.739	9.98E-15	1.60E-12	STR3
YHR197W	6945.80	2.919	0.381	7.654	1.94E-14	3.04E-12	RIX1
YNL277W	20419.19	2.066	0.270	7.648	2.04E-14	3.11E-12	MET2
YJR137C	49917.01	2.104	0.278	7.559	4.06E-14	5.92E-12	MET5
YDR021W	2082.57	2.573	0.342	7.518	5.56E-14	7.75E-12	FAL1
YNL191W	4956.97	2.187	0.294	7.450	9.31E-14	1.22E-11	DUG3
YFR030W	37005.77	2.154	0.299	7.203	5.88E-13	7.26E-11	MET10
YCL054W	15584.52	2.069	0.288	7.188	6.58E-13	7.96E-11	SPB1
YNL141W	9942.61	3.348	0.471	7.116	1.11E-12	1.32E-10	AAH1
YDL059C	5456.25	1.984	0.280	7.089	1.35E-12	1.58E-10	RAD59
YIL074C	19029.25	1.944	0.277	7.018	2.26E-12	2.54E-10	SER33
YPL012W	20592.32	2.096	0.299	6.999	2.57E-12	2.84E-10	RRP12
YLL055W	11274.19	2.700	0.390	6.914	4.70E-12	4.94E-10	YCT1
YER091C	49902.39	2.091	0.307	6.809	9.80E-12	9.83E-10	MET6
YLR223C	10503.79	1.913	0.285	6.708	1.98E-11	1.71E-09	IFH1
YMR229C	44440.72	2.099	0.313	6.696	2.15E-11	1.84E-09	RRP5
YCR087C-A	6288.92	2.416	0.364	6.632	3.31E-11	2.79E-09	YCR087C-A
YLR092W	16357.59	1.895	0.287	6.595	4.25E-11	3.54E-09	SUL2
YEL040W	2062.31	1.922	0.292	6.578	4.78E-11	3.93E-09	UTR2
YPL217C	23867.68	1.999	0.304	6.574	4.91E-11	3.99E-09	BMS1
YLR364W	2752.09	2.144	0.327	6.555	5.55E-11	4.45E-09	GRX8
YKL078W	3479.60	2.587	0.395	6.543	6.04E-11	4.78E-09	DHR2
YGR145W	11937.54	2.119	0.325	6.524	6.83E-11	5.28E-09	ENP2
YDR060W	17539.30	1.761	0.272	6.481	9.10E-11	6.95E-09	MAK21
YOR337W	6253.63	1.807	0.281	6.434	1.24E-10	9.17E-09	TEA1
YGR245C	11498.83	2.108	0.328	6.427	1.30E-10	9.48E-09	SDA1
YBL039C	13919.54	2.478	0.386	6.423	1.34E-10	9.56E-09	URA7
YML093W	18970.96	2.002	0.313	6.393	1.62E-10	1.13E-08	UTP14

YBL004W	33784.67	1.894	0.296	6.389	1.67E-10	1.15E-08	UTP20
YKL014C	16547.21	1.947	0.305	6.386	1.70E-10	1.15E-08	URB1
YNL182C	3978.85	1.862	0.293	6.366	1.95E-10	1.30E-08	IPI3
YDL031W	8598.75	1.776	0.280	6.350	2.16E-10	1.40E-08	DBP10
YOR375C	148093.21	1.909	0.302	6.318	2.65E-10	1.67E-08	GDH1
YEL065W	5884.21	1.820	0.290	6.282	3.34E-10	2.00E-08	SIT1
YKL143W	12222.64	2.330	0.371	6.277	3.46E-10	2.04E-08	LTV1
YOR341W	27018.74	1.840	0.295	6.233	4.58E-10	2.60E-08	RPA190
YBR291C	9052.39	2.243	0.361	6.219	4.99E-10	2.76E-08	CTP1
YDL148C	17595.09	2.108	0.340	6.194	5.88E-10	3.17E-08	NOP14
YJL050W	20735.09	1.895	0.307	6.166	7.00E-10	3.71E-08	MTR4
YDL153C	15876.56	1.934	0.316	6.129	8.85E-10	4.58E-08	SAS10
YGR103W	26783.78	2.399	0.393	6.104	1.03E-09	5.23E-08	NOP7
YDR502C	41437.14	1.639	0.269	6.099	1.07E-09	5.34E-08	SAM2
YBR267W	11189.67	1.775	0.291	6.095	1.09E-09	5.39E-08	REI1
YLR409C	15547.17	1.873	0.308	6.080	1.20E-09	5.88E-08	UTP21
YOR272W	7644.72	2.017	0.332	6.077	1.22E-09	5.94E-08	YTM1
YJL200C	39196.33	1.789	0.295	6.072	1.26E-09	6.03E-08	ACO2
YCL059C	11217.66	2.077	0.343	6.055	1.41E-09	6.65E-08	KRR1
YMR128W	17792.66	1.783	0.294	6.054	1.41E-09	6.65E-08	ECM16
YER073W	16358.08	1.692	0.280	6.044	1.51E-09	7.05E-08	ALD5
YJL033W	13839.37	1.879	0.313	6.012	1.84E-09	8.36E-08	HCA4
YNL221C	11174.36	1.648	0.274	6.003	1.94E-09	8.76E-08	POP1
YAL059W	6256.24	2.230	0.374	5.969	2.39E-09	1.06E-07	ECM1
YIR026C	5977.39	1.957	0.329	5.953	2.64E-09	1.16E-07	YVH1
YDL063C	5238.31	1.853	0.311	5.948	2.71E-09	1.18E-07	SYO1
YOL136C	4794.18	1.903	0.320	5.944	2.77E-09	1.20E-07	PFK27
YPR010C	25821.60	1.792	0.302	5.928	3.06E-09	1.32E-07	RPA135
YOL064C	6305.99	1.717	0.290	5.926	3.10E-09	1.32E-07	MET22
YDL112W	6658.14	1.652	0.279	5.925	3.13E-09	1.33E-07	TRM3
YBR115C	26051.64	1.663	0.283	5.877	4.18E-09	1.74E-07	LYS2
YER006W	19266.28	1.904	0.324	5.871	4.32E-09	1.79E-07	NUG1
YMR301C	2674.87	1.823	0.311	5.857	4.70E-09	1.91E-07	ATM1
YOR108W	2293.58	1.616	0.276	5.858	4.68E-09	1.91E-07	LEU9
YPR145W	49603.85	1.862	0.319	5.843	5.13E-09	2.06E-07	ASN1
YNL095C	659.84	1.887	0.324	5.831	5.50E-09	2.19E-07	YNL095C
YHR081W	7319.13	1.908	0.328	5.825	5.70E-09	2.26E-07	LRP1
YLL008W	20995.15	2.107	0.362	5.817	5.98E-09	2.34E-07	DRS1
YKL216W	7390.83	2.002	0.345	5.803	6.50E-09	2.53E-07	URA1
YNL175C	10406.43	1.912	0.331	5.775	7.71E-09	2.98E-07	NOP13
YLR222C-A	414.93	1.888	0.328	5.753	8.75E-09	3.32E-07	YLR222C-A
YBR213W	1453.39	1.694	0.295	5.749	8.97E-09	3.38E-07	MET8
YDR465C	13895.02	2.371	0.413	5.736	9.71E-09	3.61E-07	RMT2
YDR087C	6951.50	1.998	0.349	5.719	1.07E-08	3.88E-07	RRP1
YBR247C	5375.02	1.683	0.296	5.690	1.27E-08	4.57E-07	ENP1

YPR138C	6640.32	1.711	0.302	5.665	1.47E-08	5.27E-07	MEP3
YBR084W	17555.70	2.096	0.372	5.637	1.73E-08	6.11E-07	MIS1
YIL019W	3812.23	1.771	0.315	5.622	1.89E-08	6.58E-07	FAF1
YBR092C	8014.74	1.884	0.336	5.613	1.99E-08	6.91E-07	PHO3
YHR208W	8865.24	1.581	0.282	5.607	2.06E-08	7.12E-07	BAT1
YDR234W	25319.14	1.619	0.289	5.599	2.16E-08	7.40E-07	LYS4
YPR112C	20240.89	2.024	0.362	5.596	2.20E-08	7.51E-07	MRD1
YLL034C	9451.74	1.789	0.320	5.586	2.33E-08	7.87E-07	RIX7
YCL036W	5757.35	2.008	0.362	5.551	2.84E-08	9.31E-07	GFD2
YGL171W	8173.73	1.654	0.298	5.552	2.83E-08	9.31E-07	ROK1
YML056C	8867.54	2.271	0.409	5.551	2.84E-08	9.31E-07	IMD4
YDL213C	13705.72	1.789	0.323	5.541	3.00E-08	9.77E-07	NOP6
YPL266W	7411.77	2.050	0.370	5.540	3.02E-08	9.78E-07	DIM1
YOR095C	6086.24	1.756	0.318	5.517	3.45E-08	1.11E-06	RKI1
YPL043W	25044.30	1.812	0.329	5.513	3.52E-08	1.12E-06	NOP4
YGR155W	35231.82	1.787	0.325	5.502	3.75E-08	1.18E-06	CYS4
YKR079C	4443.49	1.514	0.276	5.476	4.35E-08	1.36E-06	TRZ1
YNL132W	8483.20	1.559	0.285	5.472	4.45E-08	1.38E-06	KRE33
YDR398W	8821.28	1.812	0.331	5.470	4.50E-08	1.38E-06	UTP5
YHR179W	4868.14	2.005	0.367	5.467	4.58E-08	1.40E-06	OYE2
YEL072W	2330.49	1.551	0.286	5.424	5.84E-08	1.74E-06	RMD6
YAL025C	14148.16	1.639	0.302	5.422	5.90E-08	1.75E-06	MAK16
YLR196W	18096.55	1.847	0.342	5.395	6.84E-08	1.98E-06	PWP1
YGR123C	3678.17	1.642	0.305	5.389	7.08E-08	2.03E-06	PPT1
YBR296C	2103.06	2.525	0.472	5.344	9.09E-08	2.55E-06	PHO89
YIR017C	13795.61	1.447	0.272	5.320	1.04E-07	2.88E-06	MET28
YER001W	5890.79	1.677	0.315	5.317	1.05E-07	2.91E-06	MNN1
YNR050C	75674.70	1.868	0.354	5.278	1.31E-07	3.59E-06	LYS9
YBR142W	9496.16	1.739	0.329	5.277	1.31E-07	3.59E-06	MAK5
YDR324C	13773.29	1.687	0.320	5.265	1.40E-07	3.77E-06	UTP4
YPL093W	26675.45	1.668	0.317	5.260	1.44E-07	3.86E-06	NOG1
YKL172W	21667.98	1.504	0.286	5.256	1.47E-07	3.93E-06	EBP2
YLR197W	27440.47	1.674	0.319	5.255	1.48E-07	3.93E-06	NOP56
YGL078C	15814.25	1.739	0.331	5.252	1.50E-07	3.99E-06	DBP3
YDR496C	10625.92	1.474	0.282	5.227	1.73E-07	4.45E-06	PUF6
YJR097W	2193.66	2.000	0.383	5.223	1.76E-07	4.52E-06	JJJ3
YLR342W-A	1362.67	1.472	0.282	5.213	1.86E-07	4.72E-06	YLR342W-A
YCR057C	14796.27	1.632	0.313	5.210	1.89E-07	4.78E-06	YCR057C
YML018C	5292.97	1.519	0.292	5.205	1.94E-07	4.88E-06	YML018C
YIL094C	47516.55	1.660	0.319	5.203	1.97E-07	4.93E-06	LYS12
YOR004W	6294.34	1.859	0.358	5.196	2.04E-07	5.08E-06	UTP23
YDR101C	8809.05	1.758	0.339	5.193	2.07E-07	5.14E-06	ARX1
YER049W	11404.34	1.573	0.303	5.184	2.17E-07	5.31E-06	TPA1
YNL248C	9322.06	1.683	0.325	5.182	2.20E-07	5.36E-06	RPA49
YDL060W	12091.51	1.556	0.301	5.175	2.27E-07	5.50E-06	TSR1

YPL274W	17753.16	1.733	0.335	5.174	2.29E-07	5.52E-06	SAM3
YIL096C	5331.34	1.977	0.383	5.161	2.46E-07	5.88E-06	BMT5
YBL054W	3829.84	1.412	0.274	5.148	2.63E-07	6.22E-06	TOD6
YLR002C	9201.58	1.627	0.316	5.142	2.72E-07	6.38E-06	NOC3
YNL308C	20062.06	1.508	0.294	5.125	2.97E-07	6.95E-06	KRI1
YMR217W	18199.80	1.795	0.350	5.122	3.02E-07	7.02E-06	GUA1
YLR129W	16165.92	1.593	0.311	5.120	3.06E-07	7.08E-06	DIP2
YOR342C	3028.78	1.468	0.287	5.113	3.17E-07	7.31E-06	YOR342C
YBR208C	16118.63	1.659	0.325	5.105	3.31E-07	7.56E-06	DUR12
YLR401C	5980.93	1.501	0.294	5.105	3.30E-07	7.56E-06	DUS3
YLR384C	24055.73	1.468	0.288	5.098	3.42E-07	7.76E-06	IKI3
YLR226W	1360.95	1.556	0.306	5.085	3.68E-07	8.30E-06	BUR2
YDR183C-A	521.07	1.901	0.374	5.078	3.80E-07	8.51E-06	YDR183C-A
YLR222C	10269.21	1.413	0.278	5.077	3.84E-07	8.55E-06	UTP13
YLR435W	4058.50	1.666	0.328	5.075	3.88E-07	8.58E-06	TSR2
YBR271W	1662.22	1.426	0.282	5.055	4.30E-07	9.35E-06	EFM2
YGR200C	13858.28	1.569	0.311	5.054	4.33E-07	9.39E-06	ELP2
YKR024C	9717.24	1.801	0.357	5.045	4.53E-07	9.70E-06	DBP7
YIR034C	7292.12	1.494	0.296	5.043	4.58E-07	9.77E-06	LYS1
YOL164W	4004.43	1.385	0.275	5.038	4.70E-07	9.97E-06	BDS1
YPL211W	5605.42	1.842	0.366	5.033	4.84E-07	1.02E-05	NIP7
YDR120C	12378.49	1.640	0.326	5.028	4.95E-07	1.03E-05	TRM1
YGL120C	17051.67	1.548	0.308	5.029	4.94E-07	1.03E-05	PRP43
YHL036W	1782.74	1.513	0.301	5.022	5.11E-07	1.05E-05	MUP3
YHR089C	14821.74	1.702	0.339	5.016	5.28E-07	1.09E-05	GAR1
YHR170W	21780.27	1.754	0.350	5.013	5.36E-07	1.10E-05	NMD3
YMR290C	4865.94	1.373	0.274	5.010	5.45E-07	1.11E-05	HAS1
YNR012W	4859.18	1.669	0.334	4.993	5.95E-07	1.19E-05	URK1
YLR175W	29026.74	1.493	0.299	4.985	6.20E-07	1.24E-05	CBF5
YNL075W	8847.71	1.762	0.354	4.976	6.49E-07	1.29E-05	IMP4
YLR276C	14975.01	1.588	0.321	4.947	7.53E-07	1.46E-05	DBP9
YKR081C	8033.22	1.388	0.281	4.945	7.60E-07	1.47E-05	RPF2
YOR287C	5119.54	1.575	0.318	4.945	7.63E-07	1.47E-05	RRP36
YFL023W	6998.70	1.333	0.270	4.928	8.31E-07	1.58E-05	BUD27
YNR054C	1403.08	1.578	0.320	4.925	8.43E-07	1.60E-05	ESF2
YOR243C	9903.52	1.557	0.316	4.924	8.50E-07	1.60E-05	PUS7
YDR361C	9287.88	1.671	0.340	4.916	8.85E-07	1.66E-05	BCP1
YOL144W	4369.17	1.382	0.281	4.911	9.07E-07	1.70E-05	NOP8
YLR106C	48677.97	1.350	0.275	4.908	9.19E-07	1.72E-05	REA1
YCR047C	12156.32	1.483	0.302	4.906	9.27E-07	1.73E-05	BUD23
YOL077C	14244.88	1.757	0.358	4.904	9.39E-07	1.75E-05	BRX1
YHR066W	10548.04	1.571	0.321	4.899	9.61E-07	1.78E-05	SSF1
YMR120C	17090.60	1.556	0.318	4.890	1.01E-06	1.86E-05	ADE17
YHL037C	90.47	2.316	0.475	4.880	1.06E-06	1.95E-05	YHL037C
YOR206W	16788.77	1.496	0.307	4.878	1.07E-06	1.96E-05	NOC2

YLL052C	240.05	2.057	0.422	4.873	1.10E-06	2.01E-05	AQY2
YCL037C	1514.20	1.720	0.353	4.867	1.13E-06	2.06E-05	SRO9
YBR155W	3476.69	1.345	0.277	4.860	1.17E-06	2.12E-05	CNS1
YOL058W	24356.67	1.439	0.296	4.855	1.20E-06	2.17E-05	ARG1
YOR102W	253.80	1.753	0.362	4.849	1.24E-06	2.23E-05	YOR102W
YIL047C-A	376.37	1.587	0.328	4.841	1.29E-06	2.29E-05	YIL047C-A
YGR128C	10961.60	1.652	0.342	4.834	1.34E-06	2.38E-05	UTP8
YKR039W	36925.15	1.300	0.269	4.831	1.36E-06	2.39E-05	GAP1
YOR207C	6772.05	1.308	0.271	4.832	1.35E-06	2.39E-05	RET1
YOL124C	11167.35	1.945	0.403	4.822	1.42E-06	2.49E-05	TRM11
YMR131C	29951.64	1.689	0.351	4.819	1.45E-06	2.52E-05	RRB1
YHR088W	9825.12	1.876	0.389	4.816	1.47E-06	2.55E-05	RPF1
YJL010C	6241.08	1.411	0.293	4.813	1.48E-06	2.57E-05	NOP9
YGL029W	10307.80	1.646	0.343	4.803	1.56E-06	2.68E-05	CGR1
YLR336C	10701.07	1.354	0.282	4.804	1.56E-06	2.68E-05	SGD1
YMR309C	9307.03	1.409	0.293	4.804	1.55E-06	2.68E-05	NIP1
YMR239C	5203.59	1.658	0.346	4.788	1.69E-06	2.88E-05	RNT1
YKR042W	3220.78	1.339	0.281	4.763	1.91E-06	3.20E-05	UTH1
YDL182W	6027.97	1.446	0.304	4.757	1.96E-06	3.28E-05	LYS20
YIR032C	5869.55	1.510	0.318	4.744	2.09E-06	3.49E-05	DAL3
YLR058C	12139.76	1.335	0.282	4.739	2.14E-06	3.55E-05	SHM2
YDR184C	4338.66	1.604	0.338	4.738	2.16E-06	3.57E-05	ATC1
YGR187C	7992.11	1.517	0.320	4.735	2.19E-06	3.61E-05	HGH1
YKL009W	18809.08	1.726	0.365	4.734	2.20E-06	3.61E-05	MRT4
YIL127C	6935.87	1.637	0.346	4.733	2.21E-06	3.62E-05	RRT14
YDR528W	1443.32	1.738	0.369	4.713	2.44E-06	3.96E-05	HLR1
YDL167C	9478.16	1.376	0.292	4.711	2.46E-06	3.99E-05	NRP1
YOL125W	6268.65	1.429	0.305	4.682	2.84E-06	4.53E-05	TRM13
YMR049C	11813.52	1.326	0.284	4.678	2.90E-06	4.61E-05	ERB1
YJR041C	5180.85	1.390	0.298	4.667	3.06E-06	4.85E-05	URB2
YJR002W	17623.27	1.453	0.311	4.666	3.08E-06	4.86E-05	MPP10
YLR397C	6280.14	1.391	0.298	4.662	3.14E-06	4.92E-05	AFG2
YKL191W	4886.91	1.274	0.274	4.652	3.28E-06	5.13E-05	DPH2
YER082C	13957.78	1.425	0.307	4.639	3.50E-06	5.42E-05	UTP7
YLL011W	10099.29	1.535	0.332	4.628	3.70E-06	5.67E-05	SOF1
YJR109C	31606.87	1.331	0.289	4.612	3.99E-06	6.09E-05	CPA2
YHR085W	2989.36	1.544	0.335	4.609	4.04E-06	6.15E-05	IPI1
YHR144C	2417.44	1.398	0.304	4.604	4.14E-06	6.26E-05	DCD1
YKL021C	7924.37	1.488	0.324	4.597	4.29E-06	6.46E-05	MAK11
YPL239W	8246.29	1.413	0.307	4.597	4.28E-06	6.46E-05	YAR1
YDR165W	5839.29	1.619	0.352	4.596	4.30E-06	6.46E-05	TRM82
YLR004C	12843.53	1.587	0.346	4.583	4.58E-06	6.81E-05	THI73
YHR196W	12887.13	1.494	0.327	4.568	4.93E-06	7.31E-05	UTP9
YLR249W	164352.43	1.515	0.332	4.558	5.17E-06	7.64E-05	YEF3
YNL023C	4706.09	1.292	0.284	4.551	5.34E-06	7.85E-05	FAP1

YOR145C	17450.18	1.474	0.324	4.551	5.35E-06	7.85E-05	PNO1
YMR241W	16843.03	1.344	0.296	4.543	5.55E-06	8.11E-05	YHM2
YMR177W	2302.49	1.361	0.300	4.531	5.87E-06	8.54E-05	MMT1
YGL169W	4402.64	1.519	0.336	4.525	6.03E-06	8.70E-05	SUA5
YNL124W	5588.30	1.230	0.272	4.524	6.07E-06	8.73E-05	NAF1
YBR021W	2668.20	1.268	0.280	4.523	6.11E-06	8.74E-05	FUR4
YEL071W	10863.99	1.330	0.294	4.521	6.17E-06	8.77E-05	DLD3
YPR143W	12463.16	1.515	0.335	4.521	6.15E-06	8.77E-05	RRP15
YOL022C	5655.84	1.468	0.325	4.518	6.23E-06	8.84E-05	TSR4
YPR110C	11610.44	1.433	0.318	4.510	6.48E-06	9.15E-05	RPC40
YDR527W	5260.98	1.302	0.289	4.509	6.52E-06	9.20E-05	RBA50
YDL039C	1519.66	1.740	0.387	4.500	6.80E-06	9.55E-05	YDL039C
YIL064W	3910.43	1.374	0.306	4.487	7.22E-06	1.01E-04	EFM4
YLR068W	5390.46	1.533	0.342	4.485	7.28E-06	1.01E-04	FYV7
YOL041C	16880.47	1.511	0.337	4.484	7.34E-06	1.02E-04	NOP12
YIL164C	4155.23	1.535	0.342	4.482	7.38E-06	1.02E-04	NIT1
YJR070C	11756.63	1.504	0.336	4.482	7.38E-06	1.02E-04	LIA1
YGR061C	14846.91	1.338	0.299	4.472	7.74E-06	1.06E-04	ADE6
YHR052W	12963.84	1.635	0.366	4.472	7.75E-06	1.06E-04	CIC1
YGR271C-A	7725.31	1.553	0.349	4.453	8.46E-06	1.15E-04	EFG1
YLR342W	39282.32	1.208	0.271	4.452	8.49E-06	1.15E-04	FKS1
YMR269W	2400.63	1.360	0.306	4.447	8.71E-06	1.18E-04	YMR269W
YLR363W-A	1909.71	1.674	0.377	4.446	8.75E-06	1.18E-04	YLR363W-A
YJL148W	9670.55	1.482	0.335	4.430	9.44E-06	1.26E-04	RPA34
YLR014C	2803.22	1.217	0.275	4.422	9.80E-06	1.30E-04	PPR1
YDR309C	494.81	1.830	0.414	4.419	9.92E-06	1.31E-04	GIC2
YOR252W	5740.17	1.349	0.305	4.419	9.91E-06	1.31E-04	TMA16
YPL183C	9504.88	1.295	0.293	4.413	1.02E-05	1.34E-04	RTT10
YIR012W	14730.19	1.539	0.349	4.408	1.04E-05	1.37E-04	SQT1
YMR108W	54261.56	1.252	0.285	4.395	1.11E-05	1.45E-04	ILV2
YBL055C	2980.54	1.563	0.356	4.390	1.13E-05	1.47E-04	YBL055C
YER126C	17236.41	1.499	0.342	4.384	1.16E-05	1.51E-04	NSA2
YOL080C	3654.28	1.727	0.394	4.381	1.18E-05	1.52E-04	REX4
YMR014W	8303.97	1.210	0.276	4.380	1.19E-05	1.53E-04	BUD22
YMR189W	12139.01	1.231	0.281	4.380	1.19E-05	1.53E-04	GCV2
YBL053W	152.17	1.718	0.393	4.372	1.23E-05	1.58E-04	YBL053W
YNL207W	5790.22	1.446	0.332	4.353	1.34E-05	1.70E-04	RIO2
YLR179C	7229.18	1.362	0.313	4.344	1.40E-05	1.76E-04	YLR179C
YNL062C	4003.16	1.354	0.312	4.336	1.45E-05	1.82E-04	GCD10
YFL021W	3475.09	1.258	0.291	4.323	1.54E-05	1.92E-04	GAT1
YHR128W	3025.65	1.340	0.311	4.314	1.60E-05	1.98E-04	FUR1
YBR061C	922.95	1.296	0.301	4.300	1.70E-05	2.08E-04	TRM7
YGL111W	7444.19	1.255	0.292	4.301	1.70E-05	2.08E-04	NSA1
YGR159C	26893.74	1.596	0.371	4.301	1.70E-05	2.08E-04	NSR1
YHR040W	6000.30	1.364	0.317	4.302	1.69E-05	2.08E-04	BCD1

YPL273W	3318.66	1.330	0.309	4.300	1.71E-05	2.08E-04	SAM4
YPL207W	13005.85	1.292	0.301	4.296	1.74E-05	2.11E-04	TYW1
YLR017W	7584.40	1.376	0.320	4.296	1.74E-05	2.11E-04	MEU1
YPL126W	15368.98	1.462	0.341	4.295	1.75E-05	2.11E-04	NAN1
YML080W	6007.31	1.387	0.323	4.288	1.80E-05	2.16E-04	DUS1
YBR104W	4990.72	1.271	0.297	4.282	1.85E-05	2.22E-04	YMC2
YOR184W	10510.38	1.203	0.281	4.279	1.88E-05	2.24E-04	SER1
YDR083W	8108.43	1.298	0.304	4.276	1.91E-05	2.27E-04	RRP8
YJL098W	16133.71	1.181	0.276	4.275	1.91E-05	2.27E-04	SAP185
YNL002C	22853.62	1.430	0.335	4.271	1.95E-05	2.31E-04	RLP7
YNL313C	9078.19	1.175	0.277	4.248	2.15E-05	2.53E-04	EMW1
YJL107C	4320.90	1.777	0.420	4.236	2.28E-05	2.66E-04	YJL107C
YOR335C	11993.24	1.151	0.272	4.236	2.28E-05	2.66E-04	ALA1
YAL036C	3205.28	1.167	0.276	4.223	2.41E-05	2.79E-04	RBG1
YER042W	7393.57	1.251	0.298	4.204	2.62E-05	3.02E-04	MXR1
YNL256W	6797.47	1.199	0.285	4.200	2.67E-05	3.06E-04	FOL1
YDL208W	16104.80	1.367	0.325	4.200	2.67E-05	3.06E-04	NHP2
YPR163C	34969.90	1.297	0.309	4.197	2.70E-05	3.09E-04	TIF3
YPL160W	36873.99	1.123	0.268	4.190	2.78E-05	3.18E-04	CDC60
YIL103W	3594.13	1.423	0.340	4.188	2.81E-05	3.20E-04	DPH1
YFR001W	9297.49	1.160	0.277	4.181	2.90E-05	3.30E-04	LOC1
YDR110W	8092.11	1.283	0.307	4.176	2.97E-05	3.37E-04	FOB1
YKL099C	6302.97	1.520	0.364	4.171	3.04E-05	3.43E-04	UTP11
YKL166C	5020.00	1.235	0.296	4.166	3.10E-05	3.47E-04	TPK3
YGR121C	20594.89	1.246	0.299	4.164	3.13E-05	3.49E-04	MEP1
YLR221C	6895.89	1.316	0.316	4.164	3.12E-05	3.49E-04	RSA3
YKL082C	21084.76	1.273	0.307	4.146	3.39E-05	3.76E-04	RRP14
YOR294W	7225.55	1.320	0.319	4.137	3.53E-05	3.88E-04	RRS1
YHR065C	10299.71	1.356	0.328	4.135	3.55E-05	3.90E-04	RRP3
YIL020C	2090.28	1.338	0.324	4.134	3.57E-05	3.91E-04	HIS6
YBL076C	18730.77	1.113	0.270	4.121	3.77E-05	4.13E-04	ILS1
YCR016W	5644.72	1.252	0.304	4.120	3.79E-05	4.13E-04	RBP95
YGR283C	4299.79	1.437	0.350	4.109	3.97E-05	4.30E-04	UPA1
YIL047C	14050.20	1.179	0.287	4.108	3.99E-05	4.32E-04	SYG1
YNL163C	17020.83	1.161	0.283	4.106	4.02E-05	4.34E-04	RIA1
YKL029C	6114.30	1.140	0.278	4.095	4.23E-05	4.55E-04	MAE1
YDR091C	9075.35	1.299	0.317	4.093	4.25E-05	4.56E-04	RLI1
YIL091C	9619.38	1.129	0.276	4.094	4.24E-05	4.56E-04	UTP25
YPL187W	1355.08	1.636	0.400	4.091	4.29E-05	4.60E-04	MF(ALPHA)1
YPR060C	3143.77	1.334	0.326	4.090	4.31E-05	4.60E-04	ARO7
YGL148W	17084.92	1.158	0.283	4.088	4.36E-05	4.64E-04	ARO2
YIL046W	15240.93	1.142	0.279	4.087	4.37E-05	4.65E-04	MET30
YHR169W	4836.12	1.197	0.293	4.080	4.50E-05	4.75E-04	DBP8
YBR141C	3688.37	1.300	0.319	4.075	4.60E-05	4.84E-04	BMT2
YPR048W	3940.04	1.282	0.315	4.073	4.64E-05	4.88E-04	TAH18

YER124C	28284.19	1.435	0.353	4.060	4.90E-05	5.12E-04	DSE1
YOL141W	2910.32	1.263	0.311	4.058	4.95E-05	5.15E-04	PPM2
YER055C	12840.69	1.201	0.296	4.057	4.97E-05	5.17E-04	HIS1
YOR056C	13337.11	1.267	0.313	4.055	5.01E-05	5.19E-04	NOB1
YDR321W	5546.48	1.334	0.329	4.054	5.04E-05	5.21E-04	ASP1
YDR299W	9814.79	1.156	0.286	4.048	5.16E-05	5.33E-04	BFR2
YGL009C	49098.12	1.190	0.294	4.042	5.29E-05	5.46E-04	LEU1
YOR078W	16465.41	1.310	0.324	4.038	5.39E-05	5.55E-04	BUD21
YPL263C	8789.95	1.175	0.291	4.035	5.45E-05	5.60E-04	KEL3
YJL069C	6677.21	1.190	0.295	4.032	5.53E-05	5.67E-04	UTP18
YNL299W	4787.90	1.125	0.279	4.029	5.60E-05	5.73E-04	TRF5
YHR020W	18178.48	1.158	0.288	4.028	5.63E-05	5.75E-04	YHR020W
YPR059C	216.81	1.456	0.362	4.028	5.62E-05	5.75E-04	YPR059C
YLR056W	14256.31	1.201	0.299	4.023	5.75E-05	5.84E-04	ERG3
YNL065W	7335.01	1.690	0.420	4.022	5.78E-05	5.87E-04	AQR1
YHL016C	4012.82	1.479	0.368	4.021	5.80E-05	5.88E-04	DUR3
YNL220W	31344.00	1.260	0.314	4.009	6.09E-05	6.08E-04	ADE12
YOL140W	6294.76	1.170	0.293	3.991	6.57E-05	6.53E-04	ARG8
YBL028C	9765.22	1.505	0.377	3.991	6.59E-05	6.54E-04	YBL028C
YDR161W	5557.27	1.379	0.346	3.985	6.76E-05	6.69E-04	ACL4
YPL146C	10472.78	1.198	0.301	3.984	6.76E-05	6.69E-04	NOP53
YDR040C	7594.60	1.299	0.327	3.978	6.96E-05	6.84E-04	ENA1
YHR148W	3150.47	1.470	0.370	3.971	7.15E-05	7.01E-04	IMP3
YER110C	15289.56	1.161	0.293	3.965	7.35E-05	7.17E-04	KAP123
YIL008W	5145.45	1.203	0.304	3.959	7.52E-05	7.34E-04	URM1
YGL168W	295.04	1.454	0.367	3.957	7.59E-05	7.39E-04	HUR1
YGR152C	3992.45	1.141	0.288	3.955	7.66E-05	7.44E-04	RSR1
YOR101W	7020.92	1.380	0.350	3.946	7.95E-05	7.72E-04	RAS1
YJL088W	3375.71	1.074	0.273	3.938	8.23E-05	7.95E-04	ARG3
YLR003C	7627.58	1.313	0.335	3.924	8.72E-05	8.40E-04	CMS1
YJL087C	4165.23	1.118	0.285	3.920	8.86E-05	8.52E-04	TRL1
YNL113W	12942.29	1.262	0.323	3.912	9.16E-05	8.80E-04	RPC19
YKL201C	9836.01	1.421	0.363	3.910	9.21E-05	8.84E-04	YKL201C
YLR303W	53499.23	1.205	0.308	3.909	9.29E-05	8.89E-04	MET17
YKL181W	13811.06	1.127	0.289	3.905	9.40E-05	8.98E-04	PRS1
YOL130W	9052.14	1.085	0.278	3.905	9.42E-05	8.98E-04	ALR1
YDR399W	24420.01	1.210	0.310	3.904	9.46E-05	9.01E-04	HPT1
YIR030C	674.31	1.180	0.302	3.903	9.49E-05	9.02E-04	DCG1
YDL129W	3069.54	1.256	0.322	3.899	9.65E-05	9.15E-04	YDL129W
YOL128C	2446.08	1.335	0.344	3.878	1.05E-04	9.89E-04	YGK3
YOR006C	7238.89	1.171	0.302	3.872	1.08E-04	1.01E-03	TSR3
YDL229W	42179.25	1.207	0.312	3.864	1.11E-04	1.04E-03	SSB1
YDR222W	1830.68	1.224	0.317	3.864	1.12E-04	1.04E-03	YDR222W
YDR211W	13678.17	1.127	0.292	3.859	1.14E-04	1.06E-03	GCD6
YDR449C	5326.20	1.336	0.347	3.855	1.16E-04	1.07E-03	UTP6

YGR124W	20398.57	1.110	0.289	3.842	1.22E-04	1.11E-03	ASN2
YOR310C	5900.23	1.080	0.282	3.829	1.29E-04	1.17E-03	NOP58
YJR055W	1568.20	1.232	0.322	3.826	1.30E-04	1.18E-03	HIT1
YDR365C	29136.89	1.115	0.292	3.823	1.32E-04	1.19E-03	ESF1
YMR318C	9461.35	1.250	0.327	3.823	1.32E-04	1.19E-03	ADH6
YGR125W	14497.67	1.112	0.292	3.815	1.36E-04	1.23E-03	VSB1
YPL086C	6525.08	1.155	0.303	3.812	1.38E-04	1.24E-03	ELP3
YJR072C	10001.40	1.057	0.278	3.806	1.41E-04	1.27E-03	NPA3
YGR264C	13237.43	1.048	0.277	3.788	1.52E-04	1.35E-03	MES1
YKR060W	3936.18	1.287	0.340	3.787	1.52E-04	1.36E-03	UTP30
YDR312W	4774.28	1.074	0.284	3.780	1.57E-04	1.39E-03	SSF2
YNL110C	20589.14	1.236	0.327	3.778	1.58E-04	1.40E-03	NOP15
YHR007C	29595.28	1.121	0.297	3.776	1.59E-04	1.41E-03	ERG11
YKR092C	7674.23	1.118	0.296	3.774	1.61E-04	1.41E-03	SRP40
YOL010W	7281.08	1.224	0.325	3.769	1.64E-04	1.44E-03	RCL1
YER127W	7719.26	1.178	0.313	3.768	1.64E-04	1.44E-03	LCP5
YLL062C	2583.20	1.201	0.321	3.746	1.80E-04	1.56E-03	MHT1
YNL022C	4848.52	1.040	0.278	3.745	1.80E-04	1.56E-03	RCM1
YPL212C	14625.61	1.209	0.323	3.743	1.82E-04	1.58E-03	PUS1
YHL011C	11882.42	1.268	0.340	3.729	1.92E-04	1.65E-03	PRS3
YHR149C	4438.45	1.206	0.324	3.724	1.96E-04	1.68E-03	SKG6
YIL104C	4086.71	1.153	0.310	3.720	2.00E-04	1.71E-03	SHQ1
YBL061C	3305.16	1.115	0.300	3.718	2.01E-04	1.72E-03	SKT5
YDR098C	10448.87	1.059	0.285	3.715	2.03E-04	1.73E-03	GRX3
YHR062C	1402.12	1.044	0.281	3.711	2.07E-04	1.76E-03	RPP1
YNL112W	45929.85	1.680	0.454	3.699	2.16E-04	1.83E-03	DBP2
YDL051W	18959.22	1.259	0.342	3.686	2.28E-04	1.92E-03	LHP1
YML014W	2958.03	1.039	0.282	3.681	2.32E-04	1.95E-03	TRM9
YLR449W	6172.86	1.026	0.279	3.680	2.33E-04	1.96E-03	FPR4
YDL150W	7312.69	1.078	0.294	3.671	2.42E-04	2.02E-03	RPC53
YJL108C	790.37	1.233	0.336	3.671	2.42E-04	2.02E-03	PRM10
YGL183C	359.97	1.286	0.351	3.665	2.48E-04	2.05E-03	MND1
YBL024W	13159.07	1.034	0.282	3.662	2.50E-04	2.07E-03	NCL1
YGL147C	43210.68	1.271	0.348	3.657	2.55E-04	2.10E-03	RPL9A
YJL125C	4963.06	1.109	0.304	3.655	2.57E-04	2.12E-03	GCD14
YGR003W	3207.90	1.094	0.300	3.652	2.60E-04	2.14E-03	CUL3
YIL165C	1318.82	1.178	0.323	3.649	2.63E-04	2.16E-03	YIL165C
YKL128C	2477.83	1.114	0.305	3.648	2.65E-04	2.17E-03	PMU1
YLR343W	796.73	1.354	0.372	3.634	2.79E-04	2.27E-03	GAS2
YJL011C	2473.64	1.203	0.331	3.631	2.82E-04	2.29E-03	RPC17
YOR306C	3660.17	1.225	0.337	3.630	2.83E-04	2.30E-03	MCH5
YNL292W	1763.52	1.060	0.292	3.629	2.85E-04	2.31E-03	PUS4
YIL110W	2431.71	1.210	0.334	3.628	2.86E-04	2.32E-03	HPM1
YJR132W	7759.75	1.013	0.279	3.626	2.87E-04	2.32E-03	NMD5
YDL121C	5379.80	1.103	0.305	3.619	2.95E-04	2.38E-03	EXP1

YOR339C	317.40	1.199	0.331	3.618	2.96E-04	2.39E-03	UBC11
YNL117W	4964.31	1.048	0.290	3.613	3.03E-04	2.43E-03	MLS1
YNR053C	33059.66	1.168	0.323	3.612	3.04E-04	2.43E-03	NOG2
YDR020C	2362.73	1.199	0.332	3.611	3.06E-04	2.44E-03	DAS2
YML060W	2367.17	1.113	0.308	3.610	3.07E-04	2.44E-03	OGG1
YKL144C	3651.26	1.212	0.337	3.602	3.16E-04	2.50E-03	RPC25
YKL072W	1260.20	1.044	0.290	3.600	3.19E-04	2.52E-03	STB6
YCR072C	5077.88	1.110	0.309	3.589	3.32E-04	2.62E-03	RSA4
YDR281C	754.55	1.364	0.380	3.587	3.34E-04	2.64E-03	PHM6
YLR359W	25572.96	1.183	0.330	3.586	3.36E-04	2.64E-03	ADE13
YHL039W	4862.62	1.034	0.288	3.585	3.37E-04	2.65E-03	EFM1
YMR319C	316.48	1.219	0.340	3.582	3.41E-04	2.67E-03	FET4
YEL055C	10965.99	1.006	0.281	3.579	3.45E-04	2.70E-03	POL5
YNL061W	13241.41	1.092	0.305	3.575	3.50E-04	2.74E-03	NOP2
YJR063W	2264.31	1.086	0.304	3.572	3.54E-04	2.75E-03	RPA12
YJR154W	12911.77	1.082	0.305	3.550	3.86E-04	2.97E-03	YJR154W
YJR155W	11776.26	1.015	0.286	3.550	3.86E-04	2.97E-03	AAD10
YOR001W	2696.70	1.149	0.325	3.537	4.05E-04	3.08E-03	RRP6
YDL014W	21306.18	1.204	0.340	3.536	4.06E-04	3.09E-03	NOP1
YOR222W	10105.42	1.101	0.312	3.528	4.18E-04	3.16E-03	ODC2
YER137C	2307.97	1.080	0.306	3.525	4.24E-04	3.19E-03	YER137C
YGL256W	481.08	1.204	0.343	3.514	4.42E-04	3.31E-03	ADH4
YDR300C	5741.32	1.103	0.315	3.507	4.53E-04	3.38E-03	PRO1
YGR173W	5245.27	1.024	0.292	3.506	4.55E-04	3.39E-03	RBG2
YMR093W	6480.25	1.158	0.331	3.502	4.61E-04	3.43E-03	UTP15
YFR055W	1062.99	1.168	0.334	3.499	4.68E-04	3.47E-03	IRC7
YAL066W	54.07	1.848	0.529	3.494	4.76E-04	3.52E-03	YAL066W
YNL151C	7312.34	1.066	0.305	3.490	4.83E-04	3.56E-03	RPC31
YLR186W	8129.78	1.098	0.315	3.488	4.87E-04	3.58E-03	EMG1
YNL119W	2874.64	1.007	0.289	3.486	4.90E-04	3.60E-03	NCS2
YNL162W-A	1081.49	1.450	0.416	3.482	4.98E-04	3.65E-03	YNL162W-A
YGL253W	26596.07	1.009	0.291	3.466	5.28E-04	3.84E-03	HXK2
YLR215C	3732.77	1.038	0.300	3.462	5.37E-04	3.89E-03	CDC123
YER036C	45274.95	1.045	0.302	3.454	5.53E-04	3.99E-03	ARB1
YOR338W	9629.34	1.709	0.495	3.451	5.59E-04	4.03E-03	YOR338W
YBR182C	763.92	1.121	0.326	3.444	5.74E-04	4.11E-03	SMP1
YML108W	2765.47	1.194	0.347	3.442	5.78E-04	4.13E-03	YML108W
YHR122W	3274.56	1.099	0.320	3.436	5.91E-04	4.20E-03	CIA2
YNR039C	1344.24	1.028	0.302	3.407	6.57E-04	4.57E-03	ZRG17
YML113W	6107.43	1.005	0.295	3.407	6.57E-04	4.57E-03	DAT1
YER156C	8666.83	1.026	0.302	3.402	6.69E-04	4.64E-03	MYG1
YFL055W	2160.48	1.017	0.299	3.402	6.69E-04	4.64E-03	AGP3
YHR048W	252.43	1.355	0.402	3.369	7.54E-04	5.15E-03	YHK8
YNL145W	305.77	1.151	0.342	3.364	7.68E-04	5.23E-03	MFA2
YNL108C	578.48	1.094	0.325	3.362	7.73E-04	5.27E-03	YNL108C

YJR124C	1797.99	1.069	0.319	3.352	8.01E-04	5.44E-03	YJR124C
YOR203W	183.60	1.228	0.367	3.345	8.24E-04	5.56E-03	YOR203W
YNR027W	3232.23	1.016	0.305	3.330	8.68E-04	5.83E-03	BUD17
YOR283W	8433.51	1.053	0.318	3.315	9.17E-04	6.12E-03	YOR283W
YHR049W	3175.44	1.066	0.322	3.313	9.23E-04	6.14E-03	FSH1
YIR027C	404.48	1.164	0.352	3.306	9.48E-04	6.29E-03	DAL1
YLR121C	1163.82	1.123	0.341	3.294	9.88E-04	6.49E-03	YPS3
YGL008C	74279.89	1.081	0.329	3.288	1.01E-03	6.61E-03	PMA1
YDL152W	48.83	1.754	0.534	3.285	1.02E-03	6.67E-03	YDL152W
YPR187W	18935.51	1.031	0.314	3.285	1.02E-03	6.68E-03	RPO26
YMR141C	160.28	1.374	0.420	3.274	1.06E-03	6.90E-03	YMR141C
YMR300C	11475.93	1.026	0.313	3.272	1.07E-03	6.93E-03	ADE4
YIR031C	6361.04	1.012	0.311	3.256	1.13E-03	7.25E-03	DAL7
YDR339C	8642.75	1.071	0.330	3.248	1.16E-03	7.41E-03	FCF1
YER056C	27190.26	1.012	0.312	3.247	1.17E-03	7.43E-03	FCY2
YLR051C	7954.54	1.019	0.315	3.240	1.19E-03	7.57E-03	FCF2
YCL058W-A	4591.40	1.129	0.349	3.236	1.21E-03	7.68E-03	ADF1
YHR064C	23572.13	1.015	0.315	3.228	1.25E-03	7.89E-03	SSZ1
YKR080W	10514.70	1.167	0.363	3.218	1.29E-03	8.11E-03	MTD1
YLR074C	15963.36	1.084	0.340	3.192	1.41E-03	8.81E-03	BUD20
YHR069C	5565.70	1.104	0.347	3.185	1.45E-03	8.99E-03	RRP4
YHR203C	11885.10	1.064	0.334	3.184	1.45E-03	8.99E-03	RPS4B
YMR304C-A	1129.39	1.163	0.367	3.169	1.53E-03	9.40E-03	YMR304C-A
YML006C	1820.83	1.032	0.326	3.163	1.56E-03	9.56E-03	GIS4
YPL111W	5183.20	1.211	0.384	3.155	1.61E-03	9.79E-03	CAR1
YJR140W-A	42.77	1.707	0.542	3.151	1.63E-03	9.87E-03	YJR140W-A
YDL131W	7367.49	1.056	0.335	3.150	1.63E-03	9.91E-03	LYS21
YGL246C	5523.38	1.034	0.331	3.126	1.77E-03	1.06E-02	RAI1
YKL056C	31950.22	1.096	0.351	3.120	1.81E-03	1.08E-02	TMA19
YBR069C	7476.73	1.052	0.339	3.108	1.88E-03	1.12E-02	TAT1
YMR316W	1958.35	1.030	0.333	3.090	2.00E-03	1.17E-02	DIA1
YLR237W	32870.15	1.036	0.348	2.976	2.92E-03	1.59E-02	THI7
YGR151C	92.14	1.262	0.427	2.958	3.10E-03	1.67E-02	YGR151C
YGL014W	2367.37	1.011	0.346	2.922	3.47E-03	1.84E-02	PUF4
YPL130W	231.12	1.094	0.375	2.920	3.50E-03	1.85E-02	SPO19
YNL097C-B	362.41	1.017	0.351	2.900	3.73E-03	1.95E-02	YNL097C-B
YDR249C	1593.30	1.064	0.375	2.835	4.58E-03	2.32E-02	YDR249C
YIL046W-A	1188.73	1.023	0.362	2.825	4.73E-03	2.38E-02	YIL046W-A
YMR057C	53.67	1.375	0.499	2.755	5.87E-03	2.85E-02	YMR057C
YOR170W	59.27	1.385	0.507	2.729	6.34E-03	3.03E-02	YOR170W

Supplementary Table S3.

DESeq2 results for 574 High-DO-enriched genes with adjusted p < 0.05 and log2FC < -1.

ORF	baseMean	log2FC	lfcSE	stat	pvalue	padj	gene
YNR034W-A	50158.95	-3.719	0.280	-13.302	2.26E-40	1.45E-36	EGO4
YHR087W	11222.68	-3.290	0.276	-11.923	8.98E-33	2.88E-29	RTC3
YGR248W	7029.34	-3.838	0.363	-10.581	3.63E-26	7.77E-23	SOL4
YDR224C	32020.49	-3.153	0.305	-10.352	4.10E-25	6.58E-22	HTB1
YMR169C	22544.40	-2.856	0.279	-10.248	1.20E-24	1.29E-21	ALD3
YCL048W-A	2872.01	-2.739	0.284	-9.631	5.91E-22	4.74E-19	YCL048W-A
YGR236C	12626.74	-3.844	0.409	-9.386	6.21E-21	4.42E-18	SPG1
YKL026C	5996.00	-2.605	0.285	-9.144	6.02E-20	3.60E-17	GPX1
YNL195C	4949.31	-2.866	0.318	-9.005	2.15E-19	1.15E-16	YNL195C
YLR164W	6278.03	-2.840	0.317	-8.973	2.88E-19	1.42E-16	SHH4
YMR105C	30322.73	-2.762	0.309	-8.933	4.13E-19	1.89E-16	PGM2
YDR070C	78652.46	-3.115	0.354	-8.793	1.46E-18	6.24E-16	FMP16
YKL086W	1069.32	-2.387	0.288	-8.299	1.05E-16	3.05E-14	SRX1
YGR008C	48012.88	-2.533	0.308	-8.233	1.82E-16	4.87E-14	STF2
YOR173W	16351.52	-2.651	0.322	-8.227	1.92E-16	4.93E-14	DCS2
YFR017C	2737.92	-3.267	0.399	-8.189	2.63E-16	6.49E-14	IGD1
YDR171W	28854.63	-2.642	0.324	-8.149	3.67E-16	8.41E-14	HSP42
YER150W	2633.18	-2.449	0.300	-8.152	3.57E-16	8.41E-14	SPI1
YPR036W-A	51397.00	-2.831	0.350	-8.090	5.99E-16	1.28E-13	SPO24
YDL222C	11251.47	-2.344	0.290	-8.081	6.44E-16	1.33E-13	FMP45
YDL204W	28655.62	-3.086	0.386	-8.001	1.23E-15	2.39E-13	RTN2
YML100W	21830.37	-3.380	0.424	-7.979	1.48E-15	2.79E-13	TSL1
YMR174C	45802.52	-2.718	0.346	-7.858	3.91E-15	7.16E-13	PAI3
YKL096W	789.83	-3.040	0.391	-7.770	7.83E-15	1.36E-12	CWP1
YJL161W	2854.54	-2.293	0.296	-7.753	8.95E-15	1.47E-12	FMP33
YER053C	3529.85	-2.611	0.345	-7.570	3.72E-14	5.55E-12	PIC2
YEL039C	2614.88	-2.321	0.308	-7.536	4.84E-14	6.89E-12	CYC7
YIL100C-A	242.05	-2.674	0.356	-7.508	5.99E-14	8.06E-12	YIL100C-A
YJL144W	4542.16	-2.153	0.287	-7.507	6.03E-14	8.06E-12	ROQ1
YFR052C-A	7946.16	-2.786	0.376	-7.412	1.24E-13	1.59E-11	YFR052C-A
YPR160W	32779.26	-2.679	0.367	-7.293	3.03E-13	3.81E-11	GPH1
YFL013W-A	4377.77	-2.247	0.319	-7.051	1.78E-12	2.03E-10	YFL013W-A
YFL014W	426724.52	-2.119	0.304	-6.978	2.99E-12	3.25E-10	HSP12
YHR104W	10002.01	-2.028	0.292	-6.938	3.99E-12	4.27E-10	GRE3
YFR015C	7702.97	-2.423	0.352	-6.876	6.14E-12	6.35E-10	GSY1
YER054C	14339.58	-3.014	0.439	-6.860	6.91E-12	7.04E-10	GIP2
YML128C	46776.68	-1.936	0.284	-6.804	1.02E-11	1.00E-09	MSC1
YBR285W	3276.14	-2.163	0.319	-6.780	1.20E-11	1.17E-09	HAB1
YBL065W	162.77	-2.559	0.378	-6.775	1.24E-11	1.19E-09	YBL065W
YGR174W-A	3764.77	-2.024	0.299	-6.770	1.29E-11	1.21E-09	YGR174W-A
YER121W	7203.47	-2.306	0.342	-6.748	1.50E-11	1.39E-09	YER121W
YBR116C	2317.38	-2.615	0.388	-6.743	1.55E-11	1.42E-09	YBR116C

YLR178C	5807.49	-2.555	0.380	-6.733	1.66E-11	1.50E-09	TFS1
YOR185C	5143.65	-2.802	0.417	-6.722	1.79E-11	1.60E-09	GSP2
YKL035W	16222.48	-1.997	0.297	-6.712	1.93E-11	1.69E-09	UGP1
YMR090W	8856.71	-1.791	0.274	-6.525	6.80E-11	5.28E-09	YMR090W
YNL194C	6043.70	-2.179	0.337	-6.466	1.01E-10	7.60E-09	YNL194C
YMR250W	15602.06	-2.137	0.331	-6.459	1.05E-10	7.85E-09	GAD1
YLR390W	798.77	-2.132	0.332	-6.421	1.36E-10	9.56E-09	ECM19
YMR118C	3831.36	-2.559	0.399	-6.421	1.36E-10	9.56E-09	SHH3
YMR298W	2164.45	-1.990	0.312	-6.387	1.69E-10	1.15E-08	LIP1
YDR453C	2897.82	-1.770	0.278	-6.361	2.00E-10	1.32E-08	TSA2
YDL110C	30611.66	-1.789	0.281	-6.356	2.07E-10	1.36E-08	TMA17
YJR096W	9919.36	-1.829	0.288	-6.344	2.23E-10	1.43E-08	YJR096W
YHL021C	9006.76	-2.298	0.362	-6.343	2.26E-10	1.44E-08	AIM17
YMR175W-A	7990.35	-1.962	0.311	-6.307	2.84E-10	1.77E-08	YMR175W-A
YOL071W	6814.91	-1.902	0.302	-6.305	2.89E-10	1.78E-08	SDH5
YMR325W	112.38	-3.178	0.505	-6.298	3.02E-10	1.85E-08	PAU19
YBR126C	39085.16	-1.892	0.301	-6.287	3.24E-10	1.96E-08	TPS1
YKL107W	1718.07	-2.226	0.355	-6.277	3.46E-10	2.04E-08	YKL107W
YDR379C-A	5810.23	-1.735	0.277	-6.273	3.54E-10	2.06E-08	SDH6
YFR053C	66215.36	-2.689	0.429	-6.263	3.77E-10	2.18E-08	HXK1
YKR091W	1129.08	-2.288	0.366	-6.252	4.05E-10	2.32E-08	SRL3
YOR391C	1311.83	-1.877	0.302	-6.225	4.82E-10	2.71E-08	HSP33
YIL136W	88938.90	-2.156	0.346	-6.223	4.86E-10	2.71E-08	OM45
YOR028C	1531.48	-2.073	0.334	-6.215	5.12E-10	2.81E-08	CIN5
YNL160W	13177.87	-2.466	0.397	-6.207	5.41E-10	2.94E-08	YGP1
YNL200C	7483.67	-1.837	0.297	-6.189	6.06E-10	3.24E-08	NNR1
YCL040W	52141.95	-1.779	0.289	-6.163	7.13E-10	3.75E-08	GLK1
YDR034W-B	3585.60	-1.801	0.293	-6.147	7.90E-10	4.12E-08	CPP3
YML004C	36017.66	-1.642	0.268	-6.121	9.33E-10	4.79E-08	GLO1
YCR021C	3102.45	-2.234	0.365	-6.114	9.73E-10	4.96E-08	HSP30
YPL229W	2265.99	-1.924	0.316	-6.096	1.09E-09	5.39E-08	YPL229W
YGR088W	9899.63	-2.605	0.429	-6.076	1.23E-09	5.94E-08	CTT1
YGR256W	23312.65	-2.432	0.404	-6.023	1.71E-09	7.97E-08	GND2
YDL223C	35712.67	-2.357	0.391	-6.021	1.73E-09	7.98E-08	HBT1
YPL203W	4013.79	-2.033	0.338	-6.012	1.84E-09	8.36E-08	TPK2
YLR070C	854.45	-2.226	0.371	-6.001	1.96E-09	8.77E-08	XYL2
YPR149W	27929.57	-1.769	0.296	-5.982	2.21E-09	9.83E-08	NCE102
YKL142W	8883.34	-1.603	0.271	-5.919	3.24E-09	1.37E-07	MRP8
YPL247C	3485.35	-1.692	0.288	-5.885	3.98E-09	1.67E-07	YPL247C
YKL065W-A	6173.89	-1.868	0.318	-5.868	4.41E-09	1.81E-07	DPC7
YMR107W	125163.28	-1.917	0.328	-5.843	5.12E-09	2.06E-07	SPG4
YPR184W	20945.70	-2.102	0.361	-5.823	5.78E-09	2.27E-07	GDB1
YDL218W	1226.63	-2.513	0.436	-5.761	8.36E-09	3.21E-07	YDL218W
YBR117C	13697.00	-2.475	0.430	-5.758	8.52E-09	3.25E-07	TKL2
YJL163C	4035.56	-1.835	0.320	-5.738	9.58E-09	3.59E-07	YJL163C

YAL061W	10414.67	-1.758	0.307	-5.735	9.75E-09	3.61E-07	BDH2
YBR126W-B	829.45	-1.951	0.341	-5.724	1.04E-08	3.81E-07	YBR126W-B
YNL031C	46452.23	-1.540	0.269	-5.725	1.03E-08	3.81E-07	HHT2
YPL280W	1049.57	-1.814	0.317	-5.723	1.05E-08	3.81E-07	HSP32
YDR451C	3247.21	-1.879	0.333	-5.650	1.61E-08	5.70E-07	YHP1
YOL052C-A	105349.77	-2.400	0.425	-5.650	1.61E-08	5.70E-07	DDR2
YMR323W	1280.87	-1.848	0.329	-5.625	1.85E-08	6.49E-07	ERR3
YJL164C	11002.20	-1.934	0.346	-5.594	2.22E-08	7.54E-07	TPK1
YOR120W	15671.54	-1.629	0.292	-5.581	2.39E-08	8.03E-07	GCY1
YCR061W	1774.41	-2.249	0.403	-5.579	2.42E-08	8.09E-07	TVS1
YPL127C	12571.95	-1.646	0.296	-5.563	2.66E-08	8.83E-07	HHO1
YDL124W	40002.85	-1.779	0.322	-5.523	3.33E-08	1.07E-06	YDL124W
YAL034C	1945.12	-1.934	0.351	-5.510	3.60E-08	1.14E-06	FUN19
YNL274C	17880.20	-1.666	0.303	-5.504	3.70E-08	1.17E-06	GOR1
YER053C-A	15258.46	-1.865	0.340	-5.491	4.01E-08	1.25E-06	YER053C-A
YJR021C	2989.61	-1.753	0.321	-5.471	4.48E-08	1.38E-06	REC107
YDR513W	22660.77	-1.552	0.284	-5.463	4.69E-08	1.42E-06	GRX2
YNL030W	43452.96	-1.580	0.290	-5.455	4.91E-08	1.49E-06	HHF2
YOL084W	2770.92	-1.710	0.314	-5.439	5.35E-08	1.61E-06	PHM7
YJL142C	762.04	-1.679	0.309	-5.432	5.57E-08	1.67E-06	IRC9
YGL226C-A	2405.27	-1.833	0.338	-5.421	5.93E-08	1.75E-06	OST5
YDR074W	12858.64	-1.902	0.351	-5.412	6.25E-08	1.83E-06	TPS2
YIL087C	5354.67	-1.475	0.272	-5.412	6.23E-08	1.83E-06	AIM19
YPL240C	40554.74	-1.733	0.321	-5.399	6.71E-08	1.96E-06	HSP82
YGR067C	8863.77	-1.521	0.282	-5.398	6.75E-08	1.96E-06	YGR067C
YGR043C	25415.81	-1.688	0.313	-5.391	7.01E-08	2.02E-06	NQM1
YLL026W	48204.01	-1.934	0.359	-5.388	7.11E-08	2.03E-06	HSP104
YDL214C	9160.33	-2.397	0.445	-5.383	7.33E-08	2.08E-06	PRR2
YPL123C	4130.05	-1.546	0.288	-5.368	7.95E-08	2.25E-06	RNY1
YLL023C	2115.65	-1.914	0.357	-5.358	8.41E-08	2.37E-06	POM33
YLR149C	10726.25	-1.667	0.313	-5.325	1.01E-07	2.81E-06	GID11
YER091C-A	101.21	-2.268	0.429	-5.282	1.28E-07	3.53E-06	YER091C-A
YBR169C	20296.03	-1.950	0.370	-5.270	1.36E-07	3.71E-06	SSE2
YJL141C	7110.68	-1.708	0.324	-5.268	1.38E-07	3.73E-06	YAK1
YAL060W	13888.06	-1.927	0.367	-5.247	1.54E-07	4.06E-06	BDH1
YPL183W-A	3955.71	-1.445	0.275	-5.247	1.55E-07	4.06E-06	RTC6
YCR091W	2547.03	-1.905	0.363	-5.244	1.57E-07	4.12E-06	KIN82
YHR096C	18813.77	-1.415	0.270	-5.236	1.64E-07	4.28E-06	HXT5
YKL192C	6565.59	-1.895	0.362	-5.235	1.65E-07	4.28E-06	ACP1
YDL022W	8290.46	-1.727	0.330	-5.234	1.65E-07	4.28E-06	GPD1
YKL091C	7384.12	-1.789	0.343	-5.221	1.77E-07	4.54E-06	YKL091C
YBL064C	21610.93	-1.425	0.273	-5.219	1.80E-07	4.58E-06	PRX1
YGL222C	2707.42	-1.689	0.325	-5.195	2.04E-07	5.08E-06	EDC1
YBR054W	5789.67	-1.405	0.271	-5.187	2.14E-07	5.27E-06	YRO2
YPL186C	24401.09	-1.584	0.305	-5.186	2.14E-07	5.27E-06	UIP4

YNL036W	50275.98	-1.450	0.280	-5.178	2.24E-07	5.46E-06	NCE103
YDR533C	5907.65	-1.482	0.287	-5.168	2.37E-07	5.70E-06	HSP31
YER067W	24229.65	-1.951	0.378	-5.158	2.50E-07	5.97E-06	RGI1
YBR072W	110992.79	-1.917	0.372	-5.155	2.53E-07	6.02E-06	HSP26
YGL037C	37025.36	-1.692	0.329	-5.148	2.64E-07	6.22E-06	PNC1
YBR009C	45054.72	-1.507	0.294	-5.124	3.00E-07	7.00E-06	HHF1
YBL002W	30759.88	-1.419	0.278	-5.108	3.25E-07	7.47E-06	HTB2
YIL057C	69150.42	-2.071	0.406	-5.101	3.37E-07	7.67E-06	RGI2
YPR151C	14922.44	-1.515	0.297	-5.097	3.46E-07	7.81E-06	SUE1
YMR206W	4317.02	-1.833	0.361	-5.080	3.77E-07	8.45E-06	YMR206W
YJR045C	30323.92	-1.760	0.347	-5.075	3.86E-07	8.58E-06	SSC1
YEL011W	14615.22	-1.999	0.395	-5.067	4.04E-07	8.87E-06	GLC3
YIL034C	7203.66	-1.849	0.365	-5.068	4.03E-07	8.87E-06	CAP2
YIL124W	11660.28	-1.488	0.294	-5.062	4.15E-07	9.09E-06	AYR1
YNR009W	2132.82	-1.511	0.299	-5.060	4.19E-07	9.13E-06	NRM1
YGR127W	2286.77	-1.474	0.292	-5.051	4.40E-07	9.51E-06	YGR127W
YLR109W	17309.53	-2.307	0.457	-5.046	4.52E-07	9.69E-06	AHP1
YOL040C	35187.79	-1.986	0.394	-5.046	4.51E-07	9.69E-06	RPS15
YPR172W	3598.24	-1.497	0.297	-5.038	4.71E-07	9.97E-06	YPR172W
YBR280C	3433.40	-1.559	0.310	-5.034	4.80E-07	1.01E-05	SAF1
YBR230C	33647.04	-1.444	0.287	-5.030	4.90E-07	1.03E-05	OM14
YLR258W	26567.79	-1.846	0.367	-5.030	4.92E-07	1.03E-05	GSY2
YLR110C	51598.47	-1.802	0.358	-5.026	5.01E-07	1.04E-05	CCW12
YGR052W	1795.27	-1.735	0.347	-5.005	5.59E-07	1.14E-05	FMP48
YGR209C	21640.43	-1.352	0.270	-5.003	5.63E-07	1.14E-05	TRX2
YOL048C	3297.21	-1.438	0.287	-5.003	5.63E-07	1.14E-05	RRT8
YPL230W	2808.62	-1.741	0.348	-5.002	5.67E-07	1.14E-05	USV1
YJR008W	17882.85	-1.371	0.274	-5.001	5.71E-07	1.15E-05	MHO1
YBL039W-B	2848.70	-1.380	0.276	-5.000	5.75E-07	1.15E-05	YBL039W-B
YIR039C	980.13	-1.589	0.320	-4.971	6.66E-07	1.32E-05	YPS6
YJL052W	55250.17	-1.654	0.333	-4.960	7.06E-07	1.39E-05	TDH1
YAL030W	5451.24	-1.440	0.290	-4.959	7.09E-07	1.40E-05	SNC1
YDR358W	6295.74	-1.509	0.305	-4.955	7.24E-07	1.42E-05	GGA1
YHR007C-A	4344.23	-1.369	0.276	-4.952	7.33E-07	1.43E-05	YHR007C-A
YHR140W	2199.48	-1.468	0.297	-4.952	7.36E-07	1.44E-05	YHR140W
YMR181C	3796.06	-2.191	0.443	-4.947	7.53E-07	1.46E-05	YMR181C
YDL130W-A	21337.66	-1.333	0.270	-4.945	7.63E-07	1.47E-05	STF1
YMR110C	18712.90	-1.360	0.275	-4.943	7.71E-07	1.48E-05	HFD1
YDR510W	3836.39	-2.089	0.423	-4.941	7.78E-07	1.49E-05	SMT3
YMR322C	1016.84	-1.428	0.290	-4.928	8.30E-07	1.58E-05	SNO4
YIL099W	860.57	-1.464	0.299	-4.903	9.43E-07	1.75E-05	SGA1
YGR146C	2949.24	-1.584	0.326	-4.865	1.14E-06	2.08E-05	ECL1
YDR223W	10462.18	-1.461	0.301	-4.850	1.23E-06	2.22E-05	CRF1
YMR175W	157240.18	-1.557	0.321	-4.848	1.25E-06	2.23E-05	SIP18
YBL086C	3612.32	-1.414	0.292	-4.846	1.26E-06	2.26E-05	YBL086C

YML129C	2563.27	-1.331	0.275	-4.844	1.27E-06	2.26E-05	COX14
YNL043C	192.01	-1.690	0.350	-4.831	1.36E-06	2.39E-05	YNL043C
YKL193C	7440.65	-1.472	0.305	-4.820	1.43E-06	2.51E-05	SDS22
YDL021W	6140.11	-1.501	0.313	-4.800	1.59E-06	2.73E-05	GPM2
YKL151C	12454.28	-1.378	0.288	-4.792	1.65E-06	2.82E-05	NNR2
YMR261C	6565.70	-1.512	0.316	-4.784	1.72E-06	2.93E-05	TPS3
YBR183W	3667.00	-1.340	0.280	-4.780	1.75E-06	2.97E-05	YPC1
YMR053C	3537.24	-1.596	0.334	-4.773	1.81E-06	3.07E-05	STB2
YIL033C	13414.74	-1.310	0.275	-4.770	1.84E-06	3.11E-05	BCY1
YGR194C	4281.74	-1.597	0.335	-4.764	1.90E-06	3.19E-05	XKS1
YPR193C	1411.03	-1.419	0.298	-4.760	1.93E-06	3.24E-05	HPA2
YBR120C	3176.07	-1.299	0.274	-4.743	2.10E-06	3.50E-05	CBP6
YMR196W	25958.06	-1.751	0.370	-4.735	2.20E-06	3.61E-05	YMR196W
YGL104C	1858.31	-1.375	0.291	-4.731	2.23E-06	3.64E-05	VPS73
YLR439W	1599.58	-1.650	0.350	-4.716	2.40E-06	3.91E-05	MRPL4
YDR247W	2331.46	-1.526	0.324	-4.708	2.51E-06	4.04E-05	VHS1
YOR134W	644.87	-1.573	0.334	-4.708	2.51E-06	4.04E-05	BAG7
YGR049W	6179.71	-1.905	0.405	-4.705	2.53E-06	4.08E-05	SCM4
YDR100W	3723.46	-1.313	0.279	-4.699	2.61E-06	4.19E-05	TVP15
YNL055C	48843.35	-1.768	0.376	-4.698	2.63E-06	4.21E-05	POR1
YIL051C	4390.11	-1.581	0.338	-4.678	2.90E-06	4.61E-05	MMF1
YBL078C	5700.39	-1.343	0.288	-4.664	3.10E-06	4.88E-05	ATG8
YBR053C	6685.14	-1.291	0.277	-4.662	3.13E-06	4.92E-05	YBR053C
YJL066C	4333.23	-1.571	0.338	-4.655	3.24E-06	5.06E-05	MPM1
YPL201C	8389.41	-1.935	0.417	-4.645	3.41E-06	5.31E-05	YIG1
YDL046W	8433.42	-1.677	0.361	-4.642	3.45E-06	5.36E-05	NPC2
YKR049C	9159.51	-1.251	0.270	-4.637	3.54E-06	5.48E-05	FMP46
YLL025W	257.24	-1.606	0.347	-4.634	3.58E-06	5.53E-05	PAU17
YDR085C	7716.92	-1.594	0.344	-4.631	3.63E-06	5.59E-05	AFR1
YER035W	12924.00	-1.332	0.289	-4.617	3.90E-06	5.97E-05	EDC2
YOL083W	7487.35	-1.468	0.319	-4.607	4.08E-06	6.20E-05	ATG34
YBR269C	10852.02	-1.653	0.359	-4.605	4.12E-06	6.25E-05	SDH8
YPR182W	5314.12	-1.434	0.312	-4.593	4.38E-06	6.56E-05	SMX3
YLR252W	790.64	-1.578	0.344	-4.585	4.54E-06	6.80E-05	YLR252W
YBL003C	18393.10	-1.391	0.303	-4.583	4.57E-06	6.81E-05	HTA2
YPR096C	525.63	-1.630	0.356	-4.580	4.66E-06	6.91E-05	YPR096C
YKR076W	5002.40	-2.283	0.502	-4.552	5.31E-06	7.84E-05	ECM4
YEL008C-A	76.95	-2.103	0.462	-4.548	5.41E-06	7.92E-05	YEL008C-A
YOR186W	374.45	-1.493	0.329	-4.538	5.69E-06	8.30E-05	YOR186W
YML054C	33641.36	-1.825	0.403	-4.530	5.89E-06	8.55E-05	CYB2
YEL057C	1405.37	-1.332	0.294	-4.530	5.91E-06	8.56E-05	SDD1
YBR241C	4751.77	-1.616	0.357	-4.526	6.01E-06	8.68E-05	VVS1
YHL028W	390.34	-1.971	0.436	-4.523	6.08E-06	8.73E-05	WSC4
YCL035C	24918.36	-1.218	0.269	-4.521	6.16E-06	8.77E-05	GRX1
YIL045W	2177.43	-1.645	0.365	-4.512	6.41E-06	9.08E-05	PIG2

YOL082W	5439.54	-1.396	0.310	-4.507	6.58E-06	9.25E-05	ATG19
YHR016C	5020.79	-1.348	0.300	-4.492	7.05E-06	9.87E-05	YSC84
YIL123W	5604.58	-1.586	0.354	-4.488	7.20E-06	1.01E-04	SIM1
YIR016W	12876.30	-1.408	0.314	-4.484	7.34E-06	1.02E-04	YIR016W
YJR120W	9977.62	-1.454	0.325	-4.478	7.53E-06	1.04E-04	DMO1
YOR152C	1122.07	-1.414	0.317	-4.467	7.95E-06	1.09E-04	ATG40
YMR173W-A	559.20	-1.363	0.305	-4.465	8.02E-06	1.10E-04	YMR173W-A
YNL098C	30573.82	-1.279	0.287	-4.460	8.18E-06	1.11E-04	RAS2
YNL092W	1170.49	-1.401	0.315	-4.449	8.64E-06	1.17E-04	YNL092W
YBR016W	8764.24	-1.702	0.383	-4.443	8.86E-06	1.19E-04	CPP1
YBR139W	6329.58	-1.327	0.299	-4.441	8.96E-06	1.20E-04	ATG42
YML047C	88.50	-1.905	0.429	-4.438	9.09E-06	1.22E-04	PRM6
YPR015C	1284.04	-1.281	0.289	-4.433	9.28E-06	1.24E-04	YPR015C
YLR108C	1558.16	-1.282	0.289	-4.432	9.35E-06	1.25E-04	YLR108C
YPL036W	20853.59	-1.377	0.311	-4.426	9.59E-06	1.27E-04	PMA2
YBR056W	9262.27	-1.260	0.285	-4.416	1.01E-05	1.32E-04	MRX18
YDR155C	29981.94	-1.253	0.285	-4.402	1.07E-05	1.40E-04	CPR1
YDL012C	2672.86	-1.367	0.311	-4.401	1.08E-05	1.41E-04	YDL012C
YDL085W	13786.50	-2.236	0.509	-4.394	1.11E-05	1.45E-04	NDE2
YEL030W	2249.75	-1.251	0.285	-4.388	1.14E-05	1.48E-04	ECM10
YHL030W-A	253.76	-1.482	0.339	-4.370	1.24E-05	1.59E-04	YHL030W-A
YLR446W	1188.15	-1.420	0.325	-4.366	1.27E-05	1.62E-04	NGK1
YPL147W	16617.69	-1.648	0.378	-4.366	1.27E-05	1.62E-04	PXA1
YER179W	595.98	-1.489	0.341	-4.362	1.29E-05	1.64E-04	DMC1
YMR133W	143.05	-1.628	0.374	-4.359	1.31E-05	1.66E-04	REC114
YER079W	5324.74	-1.418	0.325	-4.356	1.32E-05	1.68E-04	YER079W
YGR225W	626.34	-1.469	0.338	-4.346	1.38E-05	1.75E-04	AMA1
YAL008W	7392.58	-1.295	0.298	-4.340	1.43E-05	1.79E-04	FUN14
YGR201C	3866.20	-1.204	0.278	-4.334	1.47E-05	1.84E-04	YGR201C
YLR270W	11837.06	-1.204	0.278	-4.329	1.50E-05	1.88E-04	DCS1
YPR133W-A	9843.16	-1.367	0.316	-4.326	1.52E-05	1.90E-04	TOM5
YHL034C	31078.86	-1.679	0.389	-4.322	1.55E-05	1.92E-04	SBP1
YKL103C	7497.99	-1.487	0.344	-4.322	1.55E-05	1.92E-04	APE1
YBR230W-A	8770.35	-1.195	0.277	-4.316	1.59E-05	1.97E-04	COQ21
YOL101C	9554.23	-1.336	0.310	-4.314	1.60E-05	1.98E-04	IZH4
YEL017W	2802.63	-1.568	0.365	-4.300	1.71E-05	2.08E-04	GTT3
YPL017C	1826.66	-1.208	0.281	-4.300	1.71E-05	2.08E-04	IRC15
YFL058W	585.79	-1.644	0.383	-4.295	1.74E-05	2.11E-04	THI5
YDR018C	2225.93	-1.397	0.325	-4.293	1.76E-05	2.12E-04	MLG1
YPR026W	10812.42	-1.371	0.319	-4.293	1.77E-05	2.13E-04	ATH1
YLR219W	7097.49	-1.585	0.370	-4.282	1.85E-05	2.22E-04	MSC3
YOR084W	6580.05	-1.299	0.304	-4.277	1.90E-05	2.26E-04	LPX1
YGR053C	728.70	-1.444	0.338	-4.267	1.98E-05	2.35E-04	MCO32
YMR114C	6557.25	-1.240	0.291	-4.266	1.99E-05	2.35E-04	YMR114C
YHR008C	34810.85	-1.195	0.280	-4.263	2.01E-05	2.38E-04	SOD2

YBR214W	2410.37	-2.069	0.486	-4.257	2.07E-05	2.43E-04	SDS24
YAL005C	112860.81	-1.404	0.330	-4.252	2.12E-05	2.49E-04	SSA1
YDR133C	24514.56	-1.232	0.290	-4.245	2.18E-05	2.56E-04	YDR133C
YOR083W	3245.79	-1.155	0.273	-4.232	2.32E-05	2.70E-04	WHI5
YOR285W	48310.34	-1.145	0.271	-4.228	2.36E-05	2.74E-04	RDL1
YIL160C	9167.07	-1.660	0.393	-4.223	2.41E-05	2.79E-04	POT1
YOL126C	18354.77	-1.597	0.379	-4.217	2.48E-05	2.86E-04	MDH2
YNL009W	5727.17	-1.592	0.378	-4.215	2.49E-05	2.88E-04	IDP3
YJL016W	5481.68	-1.174	0.279	-4.201	2.66E-05	3.06E-04	TPH3
YML099W-A	29.77	-2.783	0.664	-4.190	2.79E-05	3.18E-04	YML099W-A
YDR258C	18308.36	-1.309	0.314	-4.175	2.99E-05	3.38E-04	HSP78
YBR026C	16641.57	-1.623	0.389	-4.169	3.06E-05	3.46E-04	ETR1
YKL093W	5358.22	-1.217	0.292	-4.168	3.07E-05	3.46E-04	MBR1
YLR151C	2741.40	-1.388	0.333	-4.168	3.07E-05	3.46E-04	PCD1
YDR256C	21175.37	-1.903	0.457	-4.165	3.11E-05	3.49E-04	CTA1
YGR205W	5573.28	-1.203	0.289	-4.162	3.16E-05	3.52E-04	TDA10
YDR276C	29592.29	-1.319	0.318	-4.150	3.33E-05	3.70E-04	PMP3
YKL188C	5040.01	-1.782	0.430	-4.140	3.47E-05	3.84E-04	PXA2
YKL050C	8512.81	-1.160	0.280	-4.139	3.49E-05	3.86E-04	LPX2
YCR075W-A	2455.00	-1.137	0.275	-4.137	3.52E-05	3.88E-04	EGO2
YJR104C	110991.42	-1.144	0.277	-4.137	3.52E-05	3.88E-04	SOD1
YJL185C	1542.64	-1.386	0.336	-4.129	3.65E-05	4.00E-04	ATG36
YGR249W	313.25	-1.330	0.323	-4.119	3.81E-05	4.15E-04	MGA1
YGR142W	6896.95	-1.293	0.314	-4.114	3.88E-05	4.22E-04	BTN2
YLR267W	740.28	-1.476	0.359	-4.109	3.97E-05	4.30E-04	BOP2
YGR174C	14087.82	-1.233	0.301	-4.097	4.18E-05	4.51E-04	CBP4
YDR154C	2927.47	-1.214	0.297	-4.091	4.30E-05	4.60E-04	YDR154C
YDL197C	6037.95	-1.317	0.323	-4.084	4.42E-05	4.70E-04	ASF2
YDR156W	1603.77	-1.520	0.372	-4.082	4.46E-05	4.72E-04	RPA14
YIR038C	15940.19	-1.185	0.290	-4.083	4.45E-05	4.72E-04	GTT1
YLR044C	151222.39	-1.566	0.384	-4.079	4.53E-05	4.78E-04	PDC1
YHR209W	1470.22	-1.324	0.325	-4.078	4.55E-05	4.79E-04	CRG1
YJR094C	970.47	-1.943	0.477	-4.069	4.71E-05	4.94E-04	IME1
YBR149W	33385.03	-1.144	0.281	-4.064	4.81E-05	5.04E-04	ARA1
YBR126W-A	540.30	-1.457	0.359	-4.063	4.85E-05	5.07E-04	MEO1
YIR014W	2280.38	-1.284	0.317	-4.056	5.00E-05	5.19E-04	VLD1
YOR065W	13504.77	-1.292	0.320	-4.034	5.49E-05	5.63E-04	CYT1
YKL150W	48693.61	-1.089	0.271	-4.025	5.69E-05	5.80E-04	MCR1
YBL075C	46571.19	-1.513	0.376	-4.020	5.83E-05	5.90E-04	SSA3
YDR516C	7439.39	-1.178	0.293	-4.016	5.91E-05	5.97E-04	EMI2
YER103W	17738.24	-1.077	0.268	-4.016	5.93E-05	5.98E-04	SSA4
YBR010W	50297.24	-1.240	0.309	-4.015	5.95E-05	5.99E-04	HHT1
YMR271C	2230.54	-1.110	0.276	-4.014	5.96E-05	5.99E-04	URA10
YIL107C	10952.62	-1.179	0.294	-4.012	6.03E-05	6.06E-04	PFK26
YDL024C	443.51	-1.459	0.364	-4.011	6.05E-05	6.06E-04	DIA3

YNR034W	2283.59	-1.143	0.285	-4.010	6.08E-05	6.08E-04	SOL1
YGL096W	3974.58	-1.132	0.283	-4.006	6.18E-05	6.17E-04	TOS8
YDR032C	14950.06	-1.075	0.269	-4.003	6.24E-05	6.22E-04	PST2
YLR356W	5183.75	-1.836	0.460	-3.988	6.65E-05	6.60E-04	ATG33
YAL017W	11866.70	-1.203	0.302	-3.984	6.78E-05	6.69E-04	PSK1
YNL196C	190.31	-1.427	0.359	-3.979	6.91E-05	6.81E-04	SLZ1
YPL223C	60458.95	-1.362	0.342	-3.977	6.98E-05	6.86E-04	GRE1
YPR127W	3172.13	-1.241	0.312	-3.974	7.08E-05	6.95E-04	YPR127W
YOR062C	4690.56	-1.210	0.305	-3.971	7.17E-05	7.01E-04	YOR062C
YJR017C	5182.95	-1.528	0.387	-3.945	7.98E-05	7.73E-04	ESS1
YKL096W-A	158488.49	-1.652	0.419	-3.944	8.01E-05	7.75E-04	CWP2
YNL093W	1592.76	-1.248	0.318	-3.929	8.53E-05	8.23E-04	YPT53
YDL244W	435.20	-1.456	0.373	-3.905	9.42E-05	8.98E-04	THI13
YER072W	6501.01	-1.573	0.404	-3.895	9.80E-05	9.29E-04	VTC1
YIL155C	48724.44	-1.388	0.357	-3.889	1.01E-04	9.52E-04	GUT2
YIL006W	1037.47	-1.304	0.336	-3.883	1.03E-04	9.76E-04	YIA6
YPL056C	91.62	-2.072	0.534	-3.881	1.04E-04	9.80E-04	LCL1
YEL070W	366.51	-1.219	0.314	-3.880	1.04E-04	9.83E-04	DSF1
YKL218C	2245.31	-1.109	0.286	-3.879	1.05E-04	9.85E-04	SRY1
YML085C	11518.15	-1.129	0.291	-3.877	1.06E-04	9.91E-04	TUB1
YHR171W	2684.75	-1.104	0.285	-3.872	1.08E-04	1.01E-03	ATG7
YJL065C	1883.21	-1.358	0.351	-3.872	1.08E-04	1.01E-03	DLS1
YDR119W-A	37693.42	-1.227	0.317	-3.872	1.08E-04	1.01E-03	COX26
YMR244C-A	5771.97	-1.069	0.277	-3.857	1.15E-04	1.06E-03	COA6
YNL015W	52263.15	-1.039	0.269	-3.856	1.15E-04	1.06E-03	PBI2
YOR273C	8504.09	-1.400	0.363	-3.856	1.15E-04	1.06E-03	TPO4
YPR028W	8162.69	-1.043	0.270	-3.856	1.15E-04	1.06E-03	YOP1
YHR160C	588.49	-1.583	0.411	-3.855	1.16E-04	1.07E-03	PEX18
YGR130C	30927.21	-1.177	0.306	-3.849	1.19E-04	1.09E-03	YGR130C
YMR173W	14535.77	-1.085	0.282	-3.846	1.20E-04	1.10E-03	DDR48
YPL113C	3990.08	-1.144	0.297	-3.845	1.21E-04	1.11E-03	YPL113C
YLR080W	2613.57	-1.136	0.295	-3.843	1.21E-04	1.11E-03	EMP46
YKR058W	3403.81	-1.288	0.335	-3.839	1.23E-04	1.13E-03	GLG1
YJL028W	139.51	-1.447	0.377	-3.839	1.24E-04	1.13E-03	YJL028W
YDR436W	2984.07	-1.386	0.361	-3.838	1.24E-04	1.13E-03	PPZ2
YDL169C	4319.58	-1.106	0.288	-3.835	1.25E-04	1.14E-03	UGX2
YMR251W-A	101281.32	-1.218	0.318	-3.826	1.30E-04	1.18E-03	HOR7
YGL010W	1001.33	-1.305	0.342	-3.821	1.33E-04	1.20E-03	MPO1
YOL032W	3844.14	-1.145	0.301	-3.804	1.42E-04	1.27E-03	OPI10
YOR382W	874.47	-1.120	0.294	-3.804	1.42E-04	1.27E-03	FIT2
YMR194C-B	3236.14	-1.038	0.274	-3.791	1.50E-04	1.34E-03	CMC4
YML028W	46409.80	-1.074	0.284	-3.784	1.54E-04	1.37E-03	TSA1
YER119C	2994.80	-1.155	0.305	-3.783	1.55E-04	1.38E-03	AVT6
YKR075C	8944.98	-1.786	0.472	-3.782	1.56E-04	1.38E-03	YKR075C
YHR138C	11950.63	-1.070	0.283	-3.777	1.59E-04	1.40E-03	YHR138C

YOL060C	5752.20	-1.153	0.305	-3.775	1.60E-04	1.41E-03	MAM3
YER101C	2035.76	-1.234	0.327	-3.773	1.61E-04	1.42E-03	AST2
YIL101C	1076.64	-1.916	0.508	-3.773	1.61E-04	1.42E-03	XBP1
YNL241C	11792.78	-1.344	0.357	-3.771	1.63E-04	1.42E-03	ZWF1
YPL016W	5840.41	-1.294	0.344	-3.766	1.66E-04	1.45E-03	SWI1
YFR045W	1526.19	-1.084	0.289	-3.759	1.71E-04	1.49E-03	MRX20
YNR076W	66.47	-2.022	0.539	-3.753	1.75E-04	1.52E-03	PAU6
YPL119C	5834.80	-1.089	0.291	-3.747	1.79E-04	1.55E-03	DBP1
YGR070W	7348.85	-1.163	0.311	-3.740	1.84E-04	1.59E-03	ROM1
YIL111W	2627.62	-1.144	0.306	-3.737	1.86E-04	1.61E-03	COX5B
YNL190W	7569.83	-1.649	0.441	-3.735	1.88E-04	1.62E-03	YNL190W
YOR027W	37762.38	-1.344	0.360	-3.732	1.90E-04	1.63E-03	STI1
YBR132C	3225.96	-1.176	0.316	-3.726	1.95E-04	1.67E-03	AGP2
YGL189C	31744.63	-1.101	0.296	-3.726	1.95E-04	1.67E-03	RPS26A
YOL038C-A	875.34	-1.123	0.302	-3.714	2.04E-04	1.74E-03	YOL038C-A
YLR304C	59810.50	-1.200	0.324	-3.708	2.09E-04	1.78E-03	ACO1
YNL179C	2798.67	-1.269	0.343	-3.706	2.11E-04	1.79E-03	YNL179C
YOR230W	18045.93	-1.268	0.343	-3.699	2.17E-04	1.83E-03	WTM1
YOR289W	9008.08	-1.045	0.283	-3.697	2.18E-04	1.85E-03	YOR289W
YDR510C-A	60.06	-1.886	0.511	-3.694	2.21E-04	1.86E-03	YDR510C-A
YIL113W	1271.63	-1.271	0.345	-3.684	2.30E-04	1.93E-03	SDP1
YPL054W	2621.74	-1.165	0.316	-3.683	2.31E-04	1.94E-03	LEE1
YNL115C	8003.25	-1.105	0.301	-3.675	2.38E-04	1.99E-03	YNL115C
YER058W	631.26	-1.284	0.350	-3.674	2.39E-04	2.00E-03	PET117
YDR214W	15563.81	-1.007	0.274	-3.672	2.41E-04	2.01E-03	AHA1
YDR435C	1834.53	-1.021	0.278	-3.670	2.42E-04	2.02E-03	PPM1
YNL173C	21169.77	-1.151	0.314	-3.668	2.44E-04	2.03E-03	MDG1
YBR076W	152.83	-1.442	0.393	-3.666	2.47E-04	2.05E-03	ECM8
YMR251W	1385.92	-1.029	0.281	-3.664	2.48E-04	2.05E-03	GTO3
YHL048W	3210.87	-1.122	0.306	-3.661	2.51E-04	2.08E-03	COS8
YCR004C	19631.96	-1.038	0.284	-3.649	2.63E-04	2.16E-03	YCP4
YIL106W	2760.16	-1.267	0.348	-3.646	2.66E-04	2.18E-03	MOB1
YML025C	3534.88	-1.252	0.344	-3.644	2.69E-04	2.20E-03	YML6
YGL229C	437.35	-1.368	0.376	-3.634	2.79E-04	2.27E-03	SAP4
YKR003W	4621.89	-1.093	0.301	-3.629	2.85E-04	2.31E-03	OSH6
YOR018W	2966.41	-1.153	0.318	-3.626	2.87E-04	2.32E-03	ROD1
YLR251W	2007.17	-1.308	0.362	-3.616	2.99E-04	2.41E-03	SYM1
YFR007W	1169.11	-1.131	0.313	-3.615	3.01E-04	2.42E-03	YFH7
YER120W	8901.80	-1.478	0.409	-3.614	3.02E-04	2.42E-03	SCS2
YMR136W	2049.06	-1.657	0.459	-3.611	3.05E-04	2.44E-03	GAT2
YLR006C	2803.79	-1.161	0.322	-3.607	3.09E-04	2.46E-03	SSK1
YJR116W	659.70	-1.230	0.341	-3.606	3.11E-04	2.48E-03	TDA4
YLR284C	7024.75	-1.367	0.381	-3.590	3.31E-04	2.62E-03	ECI1
YBR085C-A	21093.19	-1.153	0.322	-3.587	3.35E-04	2.64E-03	YBR085C-A
YJL137C	3300.64	-1.027	0.286	-3.586	3.36E-04	2.64E-03	GLG2

YCL049C	3891.65	-1.064	0.297	-3.581	3.43E-04	2.69E-03	YCL049C
YKL217W	185203.99	-1.020	0.286	-3.574	3.51E-04	2.74E-03	JEN1
YJR036C	1944.53	-1.156	0.323	-3.573	3.53E-04	2.75E-03	HUL4
YAL028W	487.96	-1.500	0.421	-3.562	3.67E-04	2.85E-03	FRT2
YKR033C	58.24	-1.786	0.502	-3.561	3.69E-04	2.86E-03	YKR033C
YLR053C	907.24	-1.197	0.337	-3.558	3.74E-04	2.90E-03	NRS1
YDR330W	6282.78	-1.190	0.334	-3.557	3.75E-04	2.90E-03	UBX5
YIR036C	8375.29	-1.024	0.288	-3.555	3.79E-04	2.93E-03	IRC24
YEL049W	180.99	-1.389	0.391	-3.549	3.86E-04	2.97E-03	PAU2
YFR003C	7964.69	-1.010	0.285	-3.549	3.86E-04	2.97E-03	YPI1
YGR082W	4259.01	-1.158	0.326	-3.550	3.85E-04	2.97E-03	TOM20
YDR016C	2927.80	-1.017	0.287	-3.545	3.93E-04	3.01E-03	DAD1
YML057C-A	37.95	-2.173	0.613	-3.543	3.96E-04	3.03E-03	YML057C-A
YPL119C-A	396.74	-1.181	0.334	-3.542	3.97E-04	3.03E-03	YPL119C-A
YDR178W	17569.39	-1.095	0.309	-3.541	3.98E-04	3.04E-03	SDH4
YGR019W	9739.15	-1.100	0.311	-3.542	3.98E-04	3.04E-03	UGA1
YLR315W	599.50	-1.094	0.310	-3.531	4.14E-04	3.14E-03	NKP2
YJL070C	2817.09	-1.145	0.324	-3.530	4.15E-04	3.15E-03	YJL070C
YAL004W	17.65	-3.879	1.101	-3.523	4.27E-04	3.21E-03	YAL004W
YNL025C	1093.63	-1.053	0.299	-3.516	4.38E-04	3.29E-03	SSN8
YIL055C	1512.47	-1.421	0.404	-3.516	4.39E-04	3.29E-03	YIL055C
YOR292C	834.47	-1.198	0.341	-3.511	4.46E-04	3.34E-03	YOR292C
YPR158W	3144.90	-1.168	0.333	-3.510	4.48E-04	3.34E-03	CUR1
YML110C	7140.99	-1.056	0.302	-3.500	4.66E-04	3.46E-03	COQ5
YBL048W	994.19	-1.035	0.296	-3.494	4.77E-04	3.52E-03	RRT1
YMR152W	5328.96	-1.015	0.291	-3.492	4.79E-04	3.54E-03	YIM1
YCR086W	816.94	-1.146	0.329	-3.484	4.93E-04	3.62E-03	CSM1
YMR052C-A	92.19	-1.649	0.474	-3.480	5.02E-04	3.67E-03	YMR052C-A
YER044C	5589.28	-1.040	0.300	-3.471	5.18E-04	3.78E-03	ERG28
YNL202W	15602.90	-1.306	0.376	-3.471	5.18E-04	3.78E-03	SPS19
YHL032C	36425.94	-1.139	0.328	-3.468	5.24E-04	3.82E-03	GUT1
YJL062W-A	4910.64	-1.012	0.292	-3.465	5.30E-04	3.84E-03	COA3
YKR067W	8557.68	-1.349	0.390	-3.465	5.31E-04	3.85E-03	GPT2
YFL042C	5435.08	-1.246	0.360	-3.462	5.37E-04	3.89E-03	LAM5
YGR192C	300408.68	-1.499	0.433	-3.460	5.40E-04	3.90E-03	TDH3
YOR042W	13530.00	-1.250	0.363	-3.448	5.64E-04	4.06E-03	CUE5
YML057W	8877.33	-1.164	0.338	-3.447	5.66E-04	4.07E-03	CMP2
YGL209W	1521.48	-1.715	0.498	-3.444	5.73E-04	4.11E-03	MIG2
YBR156C	1783.56	-1.162	0.338	-3.442	5.77E-04	4.13E-03	SLI15
YMR007W	93.60	-1.444	0.420	-3.442	5.78E-04	4.13E-03	YMR007W
YMR170C	3747.12	-1.016	0.295	-3.442	5.77E-04	4.13E-03	ALD2
YDR105C	2953.51	-1.021	0.297	-3.440	5.82E-04	4.15E-03	TMS1
YML101C	4397.39	-1.009	0.294	-3.436	5.90E-04	4.20E-03	CUE4
YER134C	1547.35	-1.051	0.306	-3.435	5.93E-04	4.21E-03	YER134C
YDL019C	10638.84	-1.125	0.328	-3.426	6.11E-04	4.32E-03	OSH2

YJL118W	1039.00	-1.086	0.317	-3.423	6.19E-04	4.37E-03	YJL118W
YOR178C	10515.87	-1.361	0.398	-3.421	6.23E-04	4.39E-03	GAC1
YPL185W	75.39	-1.612	0.471	-3.421	6.24E-04	4.39E-03	YPL185W
YJL205C	4959.59	-1.015	0.297	-3.420	6.27E-04	4.41E-03	NCE101
YDR246W	4125.07	-1.033	0.302	-3.415	6.39E-04	4.48E-03	TRS23
YLR102C	2300.96	-1.088	0.319	-3.411	6.47E-04	4.53E-03	APC9
YGL121C	22511.47	-1.018	0.298	-3.409	6.52E-04	4.55E-03	GPG1
YLR218C	3230.78	-1.347	0.396	-3.405	6.62E-04	4.60E-03	COA4
YJL132W	1511.76	-1.002	0.294	-3.404	6.63E-04	4.60E-03	YJL132W
YJR117W	4065.82	-1.394	0.411	-3.394	6.88E-04	4.74E-03	STE24
YOL114C	1067.25	-1.051	0.311	-3.381	7.21E-04	4.95E-03	PTH4
YLR370C	5226.98	-1.020	0.303	-3.372	7.46E-04	5.12E-03	ARC18
YDR470C	1607.43	-1.253	0.372	-3.371	7.49E-04	5.13E-03	UGO1
YBR284W	736.54	-1.005	0.299	-3.368	7.57E-04	5.17E-03	YBR284W
YJL059W	584.73	-1.093	0.325	-3.368	7.58E-04	5.17E-03	YHC3
YMR103C	2893.65	-1.182	0.353	-3.348	8.13E-04	5.49E-03	YMR103C
YNL332W	1264.93	-1.077	0.321	-3.349	8.12E-04	5.49E-03	THI12
YDL072C	2695.54	-1.193	0.357	-3.341	8.35E-04	5.62E-03	YET3
YBL032W	3396.13	-1.172	0.351	-3.339	8.41E-04	5.65E-03	HEK2
YPL116W	5925.35	-1.094	0.329	-3.325	8.84E-04	5.92E-03	HOS3
YCR085W	58.13	-1.744	0.526	-3.313	9.23E-04	6.14E-03	YCR085W
YLL018C-A	1338.46	-1.236	0.374	-3.306	9.48E-04	6.29E-03	COX19
YPL004C	15494.40	-1.283	0.388	-3.304	9.52E-04	6.31E-03	LSP1
YPR013C	1743.57	-1.246	0.377	-3.302	9.61E-04	6.36E-03	CMR3
YCL012C	2150.71	-1.088	0.330	-3.301	9.64E-04	6.38E-03	YCL012C
YLR203C	2802.01	-1.238	0.375	-3.299	9.71E-04	6.41E-03	MSS51
YBR011C	50529.75	-1.009	0.306	-3.295	9.84E-04	6.48E-03	IPP1
YKL214C	2834.82	-1.162	0.353	-3.290	1.00E-03	6.59E-03	YRA2
YLR450W	4335.50	-1.333	0.407	-3.273	1.06E-03	6.90E-03	HMG2
YNR001C	54409.23	-1.017	0.312	-3.257	1.12E-03	7.23E-03	CIT1
YGL052W	688.47	-1.051	0.323	-3.253	1.14E-03	7.31E-03	YGL052W
YLR390W-A	10450.33	-1.254	0.385	-3.253	1.14E-03	7.31E-03	CCW14
YAR020C	106.28	-1.600	0.493	-3.245	1.17E-03	7.45E-03	PAU7
YER063W	2872.55	-1.088	0.337	-3.227	1.25E-03	7.89E-03	THO1
YMR030W	2557.77	-1.050	0.326	-3.219	1.29E-03	8.09E-03	RSF1
YBR018C	71.15	-1.455	0.453	-3.214	1.31E-03	8.22E-03	GAL7
YGR102C	1009.37	-1.282	0.400	-3.203	1.36E-03	8.53E-03	GTF1
YFL020C	47.06	-2.023	0.632	-3.200	1.38E-03	8.60E-03	PAU5
YJL057C	5229.60	-1.003	0.315	-3.186	1.44E-03	8.98E-03	IKS1
YOR180C	2935.15	-1.250	0.393	-3.181	1.47E-03	9.08E-03	DCI1
YIL132C	692.80	-1.179	0.371	-3.175	1.50E-03	9.25E-03	CSM2
YMR006C	10204.97	-1.095	0.345	-3.170	1.52E-03	9.37E-03	PLB2
YKL127W	1081.64	-1.033	0.327	-3.162	1.57E-03	9.60E-03	PGM1
YDR096W	2732.90	-1.189	0.377	-3.153	1.61E-03	9.82E-03	GIS1
YLR438W	2973.91	-1.283	0.407	-3.148	1.64E-03	9.95E-03	CAR2

YFL039C	36362.00	-1.184	0.376	-3.144	1.67E-03	1.01E-02	ACT1
YGR086C	37453.25	-1.217	0.387	-3.141	1.68E-03	1.01E-02	PIL1
YKL066W	6504.53	-1.004	0.321	-3.131	1.74E-03	1.05E-02	YKL066W
YER143W	4419.43	-1.004	0.321	-3.125	1.78E-03	1.06E-02	DDI1
YDL199C	2449.46	-1.119	0.359	-3.113	1.85E-03	1.10E-02	YDL199C
YLR259C	21623.23	-1.038	0.334	-3.113	1.85E-03	1.10E-02	HSP60
YHR139C	5787.01	-1.117	0.360	-3.103	1.91E-03	1.13E-02	SPS100
YMR119W-A	224.21	-1.062	0.343	-3.099	1.94E-03	1.14E-02	YMR119W-A
YDR384C	25515.60	-1.556	0.503	-3.091	1.99E-03	1.17E-02	ATO3
YBR086C	6264.35	-1.260	0.409	-3.082	2.06E-03	1.21E-02	IST2
YDR113C	1948.46	-1.132	0.368	-3.081	2.06E-03	1.21E-02	PDS1
YML042W	11098.95	-1.493	0.486	-3.071	2.13E-03	1.24E-02	CAT2
YCR088W	10155.31	-1.403	0.457	-3.068	2.16E-03	1.25E-02	ABP1
YDL123W	176.74	-1.390	0.453	-3.065	2.17E-03	1.26E-02	SNA4
YGR015C	1107.06	-1.098	0.358	-3.066	2.17E-03	1.26E-02	EAT1
YPR183W	6491.36	-1.199	0.392	-3.061	2.20E-03	1.27E-02	DPM1
YKL123W	128.14	-1.166	0.382	-3.057	2.23E-03	1.29E-02	YKL123W
YCR099C	134.40	-1.146	0.375	-3.056	2.24E-03	1.29E-02	YCR099C
YGL259W	75.83	-1.394	0.457	-3.053	2.27E-03	1.30E-02	YPS5
YOL111C	1245.06	-1.030	0.338	-3.046	2.32E-03	1.32E-02	MDY2
YLR317W	543.96	-1.125	0.370	-3.041	2.36E-03	1.34E-02	YLR317W
YJL045W	4461.30	-1.166	0.384	-3.033	2.42E-03	1.37E-02	SDH9
YPL048W	17498.91	-1.018	0.336	-3.027	2.47E-03	1.39E-02	CAM1
YBR128C	1966.79	-1.066	0.353	-3.020	2.53E-03	1.42E-02	ATG14
YLR177W	3096.78	-1.125	0.373	-3.017	2.55E-03	1.43E-02	YLR177W
YLR037C	309.37	-1.181	0.392	-3.013	2.58E-03	1.44E-02	PAU23
YOR363C	4820.30	-1.244	0.414	-3.007	2.64E-03	1.46E-02	PIP2
YCL048W	114.04	-1.163	0.388	-3.000	2.70E-03	1.49E-02	SPS22
YDR122W	7244.63	-1.170	0.391	-2.992	2.77E-03	1.52E-02	KIN1
YPL200W	1155.74	-1.038	0.347	-2.992	2.77E-03	1.52E-02	CSM4
YOL100W	8420.83	-1.099	0.367	-2.990	2.79E-03	1.53E-02	PKH2
YCL004W	1291.51	-1.082	0.362	-2.984	2.84E-03	1.55E-02	YCL004W
YJR019C	7509.56	-1.363	0.457	-2.984	2.84E-03	1.55E-02	TES1
YCR069W	2664.89	-1.238	0.417	-2.966	3.02E-03	1.64E-02	CPR4
YHL031C	4737.74	-1.041	0.352	-2.959	3.09E-03	1.67E-02	GOS1
YPR099C	94.09	-1.285	0.435	-2.956	3.12E-03	1.68E-02	YPR099C
YOR227W	3145.66	-1.107	0.375	-2.950	3.18E-03	1.71E-02	HER1
YDR534C	265.05	-1.263	0.429	-2.943	3.25E-03	1.75E-02	FIT1
YEL068C	945.47	-1.069	0.364	-2.937	3.32E-03	1.78E-02	YEL068C
YGL180W	4700.87	-1.143	0.389	-2.936	3.33E-03	1.78E-02	ATG1
YOR142W	19061.95	-1.000	0.342	-2.924	3.46E-03	1.84E-02	LSC1
YDR501W	1078.76	-1.034	0.354	-2.922	3.47E-03	1.84E-02	PLM2
YER175W-A	297.20	-1.005	0.344	-2.920	3.49E-03	1.85E-02	YER175W-A
YNL058C	1350.08	-1.126	0.388	-2.903	3.69E-03	1.94E-02	YNL058C
YOL155C	15187.04	-1.463	0.509	-2.874	4.06E-03	2.10E-02	HPF1

YMR303C	218992.97	-1.299	0.453	-2.866	4.16E-03	2.15E-02	ADH2
YPR159C-A	145.58	-1.253	0.438	-2.859	4.25E-03	2.18E-02	YPR159C-A
YJL211C	47.33	-1.639	0.575	-2.853	4.33E-03	2.21E-02	YJL211C
YCL042W	93.01	-1.329	0.467	-2.847	4.41E-03	2.25E-02	YCL042W
YML083C	46.69	-1.849	0.652	-2.834	4.59E-03	2.32E-02	YML083C
YKL042W	1765.60	-1.052	0.373	-2.817	4.85E-03	2.42E-02	SPC42
YJR005C-A	66.40	-1.699	0.607	-2.801	5.09E-03	2.53E-02	LSO1
YLL019C	16126.51	-1.063	0.382	-2.782	5.40E-03	2.66E-02	KNS1
YKL037W	65.85	-1.347	0.489	-2.756	5.85E-03	2.84E-02	AIM26
YML008C	32205.64	-1.008	0.367	-2.746	6.03E-03	2.91E-02	ERG6
YGR146C-A	99.08	-1.461	0.532	-2.745	6.06E-03	2.92E-02	YGR146C-A
YER088C-A	387.83	-1.048	0.383	-2.739	6.16E-03	2.96E-02	YER088C-A
YDR380W	159.52	-1.037	0.379	-2.736	6.22E-03	2.98E-02	ARO10
YOR113W	7828.78	-1.018	0.374	-2.717	6.58E-03	3.12E-02	AZF1
YFL019C	101.95	-1.163	0.429	-2.710	6.73E-03	3.18E-02	YFL019C
YOR161C	4740.09	-1.000	0.369	-2.709	6.75E-03	3.18E-02	PNS1
YKL032C	5327.47	-1.243	0.462	-2.693	7.08E-03	3.31E-02	IXR1
YKL187C	7658.42	-2.912	1.085	-2.684	7.27E-03	3.38E-02	FAT3
YGR064W	52.23	-1.347	0.502	-2.683	7.29E-03	3.39E-02	YGR064W
YKL153W	164.59	-1.063	0.397	-2.679	7.39E-03	3.42E-02	YKL153W
YIL072W	170.07	-1.082	0.404	-2.678	7.41E-03	3.43E-02	HOP1
YJR151C	1157.44	-1.231	0.460	-2.674	7.50E-03	3.47E-02	DAN4
YIR041W	15.47	-2.267	0.853	-2.656	7.91E-03	3.63E-02	PAU15
YLR400W	71.41	-1.243	0.469	-2.650	8.05E-03	3.68E-02	YLR400W
YER119C-A	150.85	-1.033	0.394	-2.625	8.66E-03	3.91E-02	YER119C-A
YLR402W	212.68	-1.031	0.395	-2.613	8.98E-03	4.03E-02	YLR402W
YPL281C	13.74	-2.422	0.928	-2.611	9.02E-03	4.04E-02	ERR2
YJR004C	1042.57	-1.105	0.423	-2.610	9.07E-03	4.06E-02	SAG1
YDL022C-A	44.55	-1.370	0.526	-2.607	9.12E-03	4.07E-02	YDL022C-A
YNR002C	7913.87	-1.081	0.415	-2.603	9.25E-03	4.11E-02	ATO2
YIL054W	20.89	-2.059	0.799	-2.578	9.94E-03	4.35E-02	YIL054W
YER015W	10133.49	-1.222	0.476	-2.568	1.02E-02	4.46E-02	FAA2
YGL083W	1325.02	-1.030	0.401	-2.567	1.03E-02	4.47E-02	SCY1
YOR108C-A	55.49	-1.338	0.524	-2.552	1.07E-02	4.62E-02	YOR108C-A
YER032W	1595.57	-1.144	0.450	-2.542	1.10E-02	4.74E-02	FIR1
YDR406W-A	88.26	-1.058	0.417	-2.534	1.13E-02	4.84E-02	YDR406W-A
YOR374W	329152.59	-1.003	0.397	-2.527	1.15E-02	4.92E-02	ALD4

Supplementary Table S4A.

Pruned GO Biological Process results for enriched genes with setSize < 188 for clarity.

ID	Description	DO phase	set Size <188	enrich. Score	NES	p.adjust	rank	core_enrichment
GO:0000463	maturation of LSU-rRNA from tricistronic rRNA transcript (SSU-rRNA, 5.8S rRNA, LSU-rRNA)	Low	55	0.85	2.37	6.54E-09	635	SPB1/RRP5/URB1/DBP10/NOP7/YTM1/NUG1/NOP4/MAK16/MAK5/DBP3/TSR2/DBP7/NIP7/PRP43/HAS1/DBP9/RPF2/NOP8/BRX1/SSF1/RPF1/ERB1/IPI1/MAK11/RRP15/NOP12/CIC1/NSA2/BCD1/RLP7/NOP53/HIT1/SSF2/NOP15/NOP2/LAS1/RAI1/RAT1/RRP17/MRH4
GO:0000447	endonucleolytic cleavage in ITS1 to separate SSU-rRNA from 5.8S rRNA and LSU-rRNA from tricistronic rRNA transcript (SSU-rRNA, 5.8S rRNA, LSU-rRNA)	Low	44	0.88	2.35	6.54E-09	502	UTP10/RRP5/UTP14/UTP20/NOP14/SAS10/KRR1/ENP1/MRD1/ROK1/PWP2/UTP23/KRI1/DIP2/UTP13/ESF2/BUD23/NOP9/MPP10/UTP7/PNO1/LOC1/UTP11/RRS1/DBP8/BUD21/UTP18/UTP6/NOP58/RCL1/LCP5/EMG1/FCF1/FCF2
GO:0000472	endonucleolytic cleavage to generate mature 5'-end of SSU-rRNA from (SSU-rRNA, 5.8S rRNA, LSU-rRNA)	Low	34	0.90	2.34	6.54E-09	502	UTP10/RRP5/BMS1/UTP14/UTP20/NOP14/SAS10/MRD1/ROK1/PWP2/UTP23/DIP2/UTP13/ESF2/NOP9/MPP10/UTP7/PNO1/LOC1/UTP11/DBP8/BUD21/UTP18/UTP6/NOP58/RCL1/EMG1/FCF1/FCF2
GO:0000480	endonucleolytic cleavage in 5'-ETS of tricistronic rRNA transcript (SSU-rRNA, 5.8S rRNA, LSU-rRNA)	Low	33	0.90	2.33	6.54E-09	502	UTP10/RRP5/BMS1/UTP14/UTP20/NOP14/SAS10/MRD1/ROK1/PWP2/UTP23/DIP2/UTP13/ESF2/NOP9/MPP10/UTP7/LOC1/UTP11/DBP8/UTP18/UTP6/NOP58/RCL1/LCP5/EMG1/FCF1/FCF2
GO:0005978	glycogen biosynthetic process	High	24	-0.87	-2.02	2.81E-05	527	PGM1/GAC1/GLG2/GLG1/PSK1/PIG2/GSY2/GLC3/GDB1/UGP1/GIP2/GSY1/IGD1/PGM2
GO:0000055	ribosomal large subunit export from nucleus	Low	33	0.80	2.07	4.40E-05	1032	SDA1/ECM1/YVH1/NUG1/RIX7/ARX1/NMD3/BCP1/RPF1/MRT4/LOC1/RRS1/NOP53/NOG2/JJJ1/BUD20/NUP85/NUP82/RPL40B/RPL1A/NIC96/RPL1B
GO:0031167	rRNA methylation	Low	18	0.88	1.95	1.42E-04	535	SPB1/DIM1/NOP56/BMT5/BUD23/RRP8/BMT2/NOP58/RCM1/NOP2/NOP1/EMG1/SNU13
GO:0002181	cytoplasmic translation	Low	187	0.53	1.76	1.61E-04	1445	BUD27/NIP1/YEF3/TRM7/RBG1/TIF3/TPK3/SSB1/GCD6/RPL9A/RBG2/GIR2/SUP45/TIF4631/TIF35/SSZ1/RPS4B/TMA19/GIS2/GCN3/TIF5/DED1/TAE1/RPL13A/RPS1A/RPS12/RPS26B/RPS3/RPS2/RPP2B/RPS22A/RPL23A/FUN12/RPS0A/RPL35B/RPL10/RPL21A/RPS18B/RPL31B/RPS8B/ANB1/RPL15A/RPL27B/RPL20B/SUI3/GCN1/RPL24B/RPL5/RPS31/TMA46/ASC1/RPL8B/TIF2/RPL6A/RPL16A/CDC33/RPL40B/RPL1A/TIF11/RPS25B/RPL11A/RPS24B/RPL1B/RPL28/RPL21B/RPL2B/RPP1A/RPL17B/RPL4A/SES1/RPS17B/RPS7A/RPL26A/RPL11B/RPL12A/RPL39/RPL19B/RPL24A/RPS10A/RPS4A/RPL14A/RPL26B/RPS1B/RPS13/RPS20/RPL42A/RPS21B/RPL18B/RPL27A/PRT1/GCD1/RPL33B/TMA22/GCD11/RPS18A/RQC2/RPL37B/RPL23B/RPS21A/RPL12B/RPL20A
GO:0071035	nuclear polyadenylation-dependent rRNA catabolic process	Low	19	0.86	1.94	4.13E-04	803	MTR4/LRP1/TRF5/RRP6/MTR3/DIS3/RRP4/MPP6/RAI1/RAT1/AIR1/RRP45/RRP42/CSL4/RRP43/RRP46
GO:0006006	glucose metabolic process	High	63	-0.69	-1.95	6.33E-04	1232	FBP26/TPI1/GPM1/RMD5/PKP2/FYV10/CAT8/VID28/RGT1/GID10/PIB1/GID7/PKP1/SDH7/VID30/PGM1/ADH1/TDH3/ZWF1/TSA1/EMI2/PDC1/MDH2/NGK1/NDE2/GPM2/TDH1/GLK1/HXK1/PGM2

GO:0009304	tRNA transcription	Low	19	0.85	1.92	6.36E-04	652	BUD27/RET1/RPC40/RPC19/RPC53/RPC17/RPC25/RPC31/RPC34/RPO26/RPC37/RPC82/RPB5/RPC11/RPO31/RPC10
GO:0070813	hydrogen sulfide metabolic process	Low	8	0.96	1.78	7.17E-04	103	MET14/MET16/MET3/MET5/MET10/CYS4
GO:0033108	mitochondrial respiratory chain complex assembly	High	65	-0.68	-1.92	7.17E-04	785	COI1/COA2/COX16/SCO2/TAZ1/SOM1/COA1/CMC1/COX23/CBP3/MDM35/SHY1/RCF1/SDH7/SCO1/COX17/MSS51/COX19/PET191/COA4/COA3/PET117/COA6/CBP4/SDH8/CBP6/COX14/SSC1/SDH6/SDH5
GO:0006386	termination of RNA polymerase III transcription	Low	18	0.84	1.88	1.05E-03	989	RET1/RPC40/RPC19/RPC53/RPC17/RPC25/RPC31/RPC34/RPO26/RPC37/RPC82/RPB5/RPC11/RPO31/RPC10/RPB8/RPB10
GO:0098869	cellular oxidant detoxification	High	34	-0.76	-1.91	1.20E-03	423	HYR1/TSA1/GTT1/SOD1/CTA1/SOD2/GRX1/ECM4/AHP1/PRX1/GRX2/CTT1/TSA2/SRX1/GPX1
GO:0002098	tRNA wobble uridine modification	Low	30	0.76	1.92	1.32E-03	1089	IKI3/ELP2/SAP185/URM1/ELP3/TRM9/MTO1/NCS2/KTI12/CFD1/IKI1/NCS6/ELP4/UBA4/ISU2/CIA1
GO:0034605	cellular response to heat	High	65	-0.66	-1.88	1.32E-03	499	SIS1/SSN8/XBP1/TSA1/HSP78/SSA1/HSP2
GO:0035672	oligopeptide transmembrane	Low	11	0.89	1.79	1.75E-03	12	OPT1/SEO1
GO:0003333	amino acid transmembrane transport	Low	51	0.68	1.89	2.15E-03	968	MUP1/MMP1/YCT1/SAM3/MUP3/GAP1/YMC2/AQR1/VSB1/ODC2/AGP3/AVT4/TAT1/TAT2/VBA2/VBA4/CAN1/AGP1/ORT1/LYP1/YMC1/HIP1/RTC2/QDR2
GO:0071214	cellular response to abiotic stimulus	High	73	-0.63	-1.80	2.67E-03	409	FRT2/SSK1/YNL190W/XBP1/SIP18/USV1/GPD1/TPS1/GRE3/HSP12/ROQ1/STF2
GO:0000290	deadenylation-dependent	High	19	-0.84	-1.86	2.85E-03	253	DCS1/EDC2/EDC1/DCS2
GO:0034599	cellular response to oxidative stress	High	112	-0.57	-1.73	2.98E-03	423	HYR1/TSA1/MCR1/SOD1/SOD2/GRX1/SDH8/AHP1/HSP31/NCE103/PRX1/NQM1/GRX2/YDL124W/GCY1/DDR2/YJR096W/TSA2/GAD1/GRE3/HSP12/SRX1/GPX1
GO:0046185	aldehyde catabolic process	High	11	-0.91	-1.81	4.19E-03	488	YJL068C/SNO4/HFD1/HSP31/GOR1/HSP32/GLO1/HSP33
GO:0071038	TRAMP-dependent tRNA surveillance pathway	Low	16	0.84	1.82	5.09E-03	803	MTR4/TRF5/RRP6/MTR3/DIS3/RRP4/AIR1/RRP45/RRP42/CSL4/RRP43/RRP46
GO:0000056	ribosomal small subunit export from nucleus	Low	15	0.83	1.77	5.65E-03	785	LTV1/BUD23/NOP9/YAR1/PNO1/ARB1/RPS3/NUP82/SLX9
GO:0000103	sulfate assimilation	Low	12	0.88	1.80	5.72E-03	84	MET14/MET1/MET16/MET3/MET5/MET10/MET22/MET8
GO:0071028	nuclear mRNA surveillance	Low	28	0.75	1.87	5.72E-03	854	MTR4/LRP1/MRD1/RRP6/MTR3/DIS3/RRP4/MPP6/RAT1/AIR1/RRP45/RRP42/RRP43/RRP46/HRB1
GO:0051595	response to methylglyoxal	High	8	-0.94	-1.74	6.08E-03	173	SNO4/HSP31/HSP32/GLO1/HSP33
GO:0034475	U4 snRNA 3'-end processing	Low	15	0.81	1.74	8.91E-03	803	MTR4/LRP1/RNT1/TRF5/RRP6/MTR3/RRP4/RRP45/RRP42/RRP43/RRP46
GO:0009438	methylglyoxal metabolic process	High	7	-0.95	-1.71	8.97E-03	173	SNO4/HSP31/HSP32/GLO1/HSP33
GO:0030866	cortical actin cytoskeleton organization	High	61	-0.63	-1.76	9.41E-03	1350	CDH1/MSS4/AIM44/CDC11/SLA1/PRK1/ARC19/LDB17/ZIP1/PAN1/TWF1/ARC40/RVS167/EDE1/AKL1/SUR7/ARP3/ARC15/ENT2/ABP1/ACT1/SPR28/ARC18/YNL194C/FMP45
GO:0000467	exonucleolytic trimming to generate mature 3'-end of 5.8S rRNA from tricistronic rRNA transcript (SSU-	Low	18	0.80	1.79	9.85E-03	803	MTR4/LRP1/RRP6/MTR3/DIS3/RRP4/MPP6/RRP45/RRP42/RNH70/CSL4/RRP43/RRP46
GO:0072348	sulfur compound transport	Low	28	0.74	1.84	1.05E-02	617	MUP1/MMP1/SUL1/YCT1/SUL2/SAM3/MUP3/OAC1/SOA1/THI7/VHT1
GO:0006144	purine nucleobase metabolic process	Low	21	0.79	1.86	1.05E-02	881	AAH1/DAL3/SER1/HPT1/DAL1/ADE4/DAL7/MTD1/APT1/ADE57/DAL2/ADE8/ADO1/ADE2
GO:0019344	cysteine biosynthetic process	Low	10	0.88	1.71	1.09E-02	656	MET14/MET16/CYS4/MET28/MET30/MET17/HSU1/CYS3

GO:0072593	reactive oxygen species metabolic process	High	19	-0.80	-1.79	1.09E-02	886	CCP1/TRR2/LPD1/TDH3/TSA1/SOD1/CTA1/SOD2/AHP1/CTT1/TSA2
GO:0006384	transcription initiation at RNA polymerase III promoter	Low	29	0.74	1.85	1.10E-02	989	RET1/RPC40/RPC19/RPC53/RPC17/RPC25/RPC31/RPC34/RPO26/RPC37/RPC82/RPB5/RPC11/RPO31/RPC10/SUB1/RPB8/RPB10
GO:0009079	pyruvate family amino acid	Low	11	0.86	1.71	1.18E-02	430	LEU9/BAT1/ILV2/LEU1/ILV3
GO:0031126	sno(s)RNA 3'-end processing	Low	28	0.74	1.83	1.26E-02	803	MTR4/LRP1/NOP56/CBF5/TRF5/NOP58/RRP6/NOP1/MTR3/RRP4/SNU13/CTR9/RRP46
GO:0071039	nuclear polyadenylation-dependent CUT catabolic process	Low	10	0.88	1.71	1.27E-02	557	LRP1/TRF5/RRP6/MTR3/DIS3/MPP6/AIR1
GO:0009085	lysine biosynthetic process	Low	13	0.84	1.76	1.48E-02	813	ACO2/LYS2/LYS4/LYS9/LYS12/LYS1/LYS20/LYS21/PLS1/ARO8/LYS14
GO:0046496	nicotinamide nucleotide metabolic process	High	77	-0.59	-1.71	1.59E-02	816	ENO1/RPE1/ALD4/YMR315W/MDH3/ERR2/SOL2/PNP1/TDH3/YLR345W/ZWF1/GUT2/SOL1/EMI2/IDP3/NGK1/NNR2/GPM2/TDH1/NQM1/ERR3/TKL2/GND2/GLK1/NNR1/HXK1/SOL4
GO:0043255	regulation of carbohydrate biosynthetic process	High	36	-0.70	-1.77	1.61E-02	1151	PCL8/RMD5/FYV10/PCL6/CAT8/VID28/GID10/PIB1/GID7/SDH7/VID30/GAC1/TSA1/PSK1/PIG2/GIP2
GO:0110095	cellular detoxification of aldehyde	High	10	-0.89	-1.76	1.62E-02	488	YJL068C/SNO4/HSP31/HSP32/GLO1/HSP33
GO:0008535	respiratory chain complex IV assembly	High	34	-0.70	-1.75	1.68E-02	1312	COI1/COA2/COX16/SCO2/COA1/CMC1/COX23/SHY1/RCF1/SCO1/COX17/MSS51/COX19/PET191/COA4/COA3/PET117/COA6/COX14/SSC1
GO:0000398	mRNA splicing, via spliceosome	Low	120	0.49	1.56	1.68E-02	1792	FAL1/PRP43/DBP2/RAT1/SNU13/MUD2/DED1/CWC24/DRN1/CWC21/BRR2/CUS1/HSH155/SNU56/PRP31/PRP16/SLU7/PRP3/SYF1/LIN1/URN1/SPP381/CWC2/LEA1/PRP39/PRP28/PRP9/ECM2/SME1/PRP8/SNU66/SPT5/CDC40/PRP11/SNU71/SNU114/PRP2/PRP24/LOT5/MAK31/PRP6/YJU2/PRP38/PRP46/PRP21/SYF2/HSH49/LUC7/PRP19/PRP42/BRR1/SAD1/PRP22/PML1/SQS1/RPL30
GO:0045454	cell redox homeostasis	High	22	-0.76	-1.73	1.72E-02	846	TRR2/DOT5/FMP40/SCO1/TSA1/POR1/TRX2/AHP1/PRX1/TSA2/ECM19
GO:0010256	endomembrane system organization	High	156	-0.49	-1.55	1.82E-02	897	LSB6/VMA13/DOA4/RTN1/VPS13/SEC17/CNL1/NEM1/TCB1/RET2/TCB3/RVS167/BLS1/SHE3/SCS22/VAB2/ERP5/HER1/GOS1/SED5/MDY2/IST2/SCS2/HOR7/YOP1/POR1/SNC1/YGR127W/UIP4/POM33/NCE102/HSP12/RTN2
GO:0019319	hexose biosynthetic process	High	35	-0.68	-1.71	1.83E-02	1219	TPI1/GPM1/RMD5/FYV10/CAT8/VID28/GID10/PIB1/GID7/SDH7/VID30/TDH3/TSA1/MDH2/TDH1/XYL2
GO:0005992	trehalose biosynthetic process	High	7	-0.93	-1.68	1.85E-02	184	TPS3/TPS2/TPS1/UGP1/TSL1/PGM2
GO:0009086	methionine biosynthetic process	Low	19	0.78	1.75	1.99E-02	956	STR3/MET2/BAT1/SAM4/MEU1/MET17/HSU1/MRI1/ARO8/ADI1/MDE1/MET13
GO:0001522	pseudouridine synthesis	Low	17	0.79	1.75	2.01E-02	817	GAR1/CBF5/PUS7/NAF1/NHP2/TSR3/PUS1/PUS4/DEG1/NOP10
GO:0090329	regulation of DNA-templated DNA replication	Low	48	0.61	1.68	2.11E-02	1522	RIX1/IPI3/IPI1/FOB1/MSH3/MCM5/RAD9/RFM1/MSH6/CHD1/SET2/TAH11/SLD3/RCO1/DPB11/BRE1/TOP2/CTI6
GO:0016556	mRNA modification	Low	9	0.88	1.68	2.15E-02	604	KRE33/CBF5/PUS7/PUS1/PUS4/DEG1
GO:0006450	regulation of translational fidelity	Low	31	0.70	1.77	2.18E-02	816	TPA1/SUA5/ALA1/CDC60/ILS1/YHR020W/SSB1/HPM1/SSZ1/VAS1/RPS9B/RPS2/RPL31B
GO:0030437	ascospore formation	High	119	-0.51	-1.56	2.29E-02	916	VID28/BMH1/SEC14/LDS1/AQY1/SUR7/SPO74/ADY3/RIM11/BMH2/GIP1/CDC15/SPS22/SPS100/ACT1/SPR28/SPS19/EMI1/IRC18/EMI2/AMA1/CPR1/RAS2/SNC1/RRT8/SDH5/YNL194C/CWP1/FMP45/SPO24

GO:0140013	meiotic nuclear division	High	140	-0.48	-1.50	2.32E-02	1044	RAD51/MPS1/SWI6/ZIP1/SGO1/HFM1/SRS2/RFA3/NUF2/CSM3/MEK1/HOP1/RMR1/CSM4/MHF1/MLH3/PDS1/NBL1/CSM2/SLI15/CSM1/TUB1/IME1/MSC3/IRC15/AMA1/REC114/DMC1/CPR1/REC107/MSC1
GO:0009414	response to water deprivation	High	6	-0.93	-1.64	2.54E-02	369	YNL190W/SIP18/TPS1/ROQ1/STF2
GO:0006270	DNA replication initiation	Low	55	0.59	1.66	2.54E-02	1522	RIX1/IPI3/NOC3/IPI1/MCM2/MCM5/MCM3/RFM1/MCM7/MCM10/SET2/TAH11/SLD3/POL1/MCM6/RCO1/DPB11/BRE1/PRI2/ORC6/CTI6
GO:0016042	lipid catabolic process	High	69	-0.58	-1.63	2.85E-02	1225	FOX2/TGL1/CIT3/SPO1/NTE1/TGL5/POX1/PAH1/ICL2/FAA2/MDH3/LDH1/TES1/PIP2/PLB2/DCI1/SPS19/ECI1/MPO1/PXA2/IDP3/POT1/LPX1/PXA1/YPC1/YHR140W/AYR1
GO:0051156	glucose 6-phosphate metabolic process	High	21	-0.75	-1.70	2.87E-02	581	SOL2/PGM1/ZWF1/SOL1/EMI2/NGK1/NQM1/TKL2/GND2/GLK1/HXK1/PGM2/SOL4
GO:0033365	protein localization to organelle	High	447	-0.38	-1.33	2.91E-02	1049	COS10/DAD4/MGR2/KAR3/MPS1/ECM21/ZIP1/UBC13/PTP2/MTW1/PEX22/ROG3/MIM2/YET1/HOT13/TOM40/OXR1/MIM1/YET2/PEX5/ROY1/GID10/COS4/TOM6/SUR7/ATG18/PAM17/MCP1/HFL1/ATG3/MIA40/ATG1/CDC15/SNX4/ATG14/PEP4/MDY2/ABP1/ATG4/HSP60/DDI1/PIL1/COS6/DAM1/HSP10/TOM22/YET3/RCR2/VPS68/CSM1/CUR1/DAD1/YPI1/TOM20/SCS2/ROD1/COS8/AHA1/WTM1/OPI10/YGR130C/PEX18/ATG7/YPT53/BTN2/EGO2/MDH2/SSA1/APE1/TOM5/RAS2/ATG34/ATG8/POR1/VPS73/SDS22/GGA1/OM14/SSC1/GPD1/TPK1/GSP2/MSC1
GO:0006521	regulation of amino acid metabolic process	Low	20	0.77	1.78	3.06E-02	1040	MET32/MET28/ARG81/SKP2/CAR1/PLS1/LYS14/ASC1/LEU3
GO:0015711	organic anion transport	Low	121	0.49	1.55	3.23E-02	1226	MUP1/SEO1/YCT1/LTV1/CTP1/MUP3/GAP1/YHM2/YMC2/AQR1/MCH5/ODC2/AVT4/TAT1/TAT2/OAC1/CAN1/VHT1/FEN2/AGP1/HNM1/MCH4/ORT1/YMC1/TPO5/MPC2/HIP1/RTC2/QDR2/YBR219C/YBR220C/PRY2/RCH1/YJL193W/AVT2/YPQ2/FSF1/UGA4/FLC1/ANT1
GO:0040031	snRNA modification	Low	9	0.86	1.65	3.23E-02	851	GAR1/CBF5/PUS7/NHP2/PUS1/NOP10/PRP16
GO:0009073	aromatic amino acid family biosynthetic process	Low	24	0.72	1.74	3.28E-02	1364	HIS6/ARO7/ARO2/HIS1/HIS7/TRP3/ARO4/ARO8/TYR1/HIS4/TRP5/HIS5/ARO1/ADE3/ARO3/HIS2
GO:0045229	external encapsulating structure organization	High	253	-0.43	-1.43	3.30E-02	839	GAS5/LDS1/FYV5/SUR7/PBN1/PAU15/SSD1/DAN4/ADY3/FAT3/AZF1/YPK2/GIP1/HPF1/SPS22/PAU23/SPS100/ACT1/PAU5/CCW14/CMP2/IRC18/PAU2/ECM8/PAU6/CWP2/SOD1/AMA1/SIM1/WSC4/ECM4/PAU17/BAG7/YPS6/RRT8/CCW12/NCE102/YGP1/PAU19/ECM19/YNL194C/CWP1/FMP45/SPI1
GO:0042732	D-xylose metabolic process	High	6	-0.92	-1.63	3.34E-02	188	XKS1/GCY1/XYL2/YJR096W/GRE3

GO:0032543	mitochondrial translation	High	142	-0.49	-1.51	3.35E-02	1894	MRPS5/NAM2/MSF1/CBS2/MRPL49/RSM7/TIS11/GRS2/IMG2/MBA1/MRPL50/MRP2/TUF1/MRPL13/MRPL33/HER2/MRPL6/MDM38/MRP10/MHR1/MRP35/RSM19/MRPL39/MRPL17/RSO55/MRPL23/MRPL28/MRPL1/MRX14/MRPL37/MRPL8/RRF1/AIM23/MST1/DIA4/MSR1/PTH1/SLM5/MRP4/MRP49/MRPS12/QRI5/PET112/MRP20/MEF2/MRPL9/CBP3/FYV4/MRPL31/IMG1/GTF1/MSS51/PTH4/COA3/YML6/MMF1/MRPL4/CBP6/COX14/SSC1/RTC6
GO:0006528	asparagine metabolic process	Low	9	0.86	1.65	3.46E-02	609	ASN1/ASP1/ASN2/YML096W
GO:0019323	pentose catabolic process	High	7	-0.92	-1.66	3.53E-02	188	YPR1/XKS1/GCY1/XYL2/YJR096W/GRE3
GO:0006457	protein folding	High	139	-0.49	-1.51	3.53E-02	671	SBA1/MIA40/CPR4/HSP60/HSP10/SIS1/COX19/CUR1/CPR6/AHA1/STI1/TSA1/SSA4/SSA3/BTN2/HSP78/SSA1/ECM10/CPR1/SDH8/SSC1/HSP26/HSP31/SSE2/HSP104/HSP8
GO:0042256	cytosolic ribosome assembly	Low	12	0.82	1.69	3.65E-02	829	RRP5/MRD1/ESF2/RIA1/SDO1/TIF5/FUN12/RPL15A
GO:0043094	metabolic compound salvage	Low	35	0.65	1.69	3.67E-02	905	AAH1/BAT1/URK1/FUR1/SAM4/MEU1/HPT1/DAS2/BUD17/APT1/MRI1/URH1/BUD16/ARO8/ADI1/ADO1/FCY1/MDE1/NRK1
GO:0002128	tRNA nucleoside ribose methylation	Low	6	0.92	1.59	3.67E-02	537	TRM3/TRM13/RTT10/TRM7/TRM732/TRM44
GO:0006361	transcription initiation at RNA polymerase I promoter	Low	22	0.72	1.72	3.85E-02	652	RPA190/RPA135/RPA49/RPC40/RPA34/RPC19/RPA12/RPO26/RRN6/RPA43/RPB5/RPC10
GO:0042182	ketone catabolic process	High	10	-0.86	-1.69	3.87E-02	173	ARO9/SNO4/HSP31/HSP32/GLO1/HSP33
GO:0032881	regulation of polysaccharide metabolic process	High	22	-0.73	-1.66	4.06E-02	1151	PCL8/PCL6/BMH1/BMH2/GAC1/PSK1/PIG2/GIP2/IGD1
GO:0006362	transcription elongation by RNA polymerase I	Low	25	0.72	1.78	4.17E-02	1188	RPA190/RPA135/RPA49/BUD27/RPC40/RPA34/RPC19/RPA12/RPO26/RPA43/RPB5/RPC10/CTR9/RPB8/RPB10/SPT5/RTF1
GO:0006121	mitochondrial electron transport,	High	9	-0.87	-1.66	4.32E-02	578	SDH9/SDH4/SDH5/SHH3/SHH4
GO:0005980	glycogen catabolic process	High	15	-0.79	-1.67	4.38E-02	746	ATG18/ATG3/SNX4/SGA1/GDB1/GPH1/IGD1
GO:0009084	glutamine family amino acid biosynthetic process	Low	27	0.69	1.72	4.48E-02	995	GDH1/ARG1/CPA2/MEU1/ARG8/ARG3/PRO1/ARG56/CPA1/ORT1/GLN1/ARG7/GLT1/ARG4
GO:1902626	assembly of large subunit precursor of preribosome	Low	8	0.88	1.64	4.60E-02	561	DBP10/NOG1/NIP7/RPF2/NOP2/SPB4/RLP24
GO:0019346	transsulfuration	Low	9	0.85	1.62	4.63E-02	877	STR3/CYS4/MET17/IRC7/HSU1/YML082W/CYS3/YHR112C
GO:0006189	'de novo' IMP biosynthetic process	Low	9	0.85	1.62	4.74E-02	881	ADE17/ADE6/ADE13/ADE4/ADE57/ADE1/ADE8/ADE2
GO:0051258	protein polymerization	High	56	-0.59	-1.62	4.76E-02	1165	SLA1/CAP1/MMS2/ARC19/DAD4/SPC97/SPC29/UBC13/VIK1/TWF1/AIM21/STU2/SPC42/ARC15/ABP1/DAM1/ARC18/DAD1/TSA1/IRC15/CAP2/SRX1
GO:0046112	nucleobase biosynthetic process	Low	20	0.74	1.72	4.77E-02	883	AAH1/URA7/URA1/SER1/ADE4/MTD1/URA4/APT1/CPA1/ADE57/ADE8/URA5/URA2
GO:0006631	fatty acid metabolic process	High	77	-0.55	-1.59	4.81E-02	1225	FOX2/CIT3/HST3/PKP2/ACB1/MCT1/TML25/POX1/CYC8/PAH1/ICL2/FAA2/MDH3/FAA1/PKP1/TES1/PIP2/CAT2/DCI1/SPS19/ECI1/MPO1/PXA2/ETR1/IDP3/POT1/PXA1/YHR140W/ACP1/TMA17
GO:0017183	protein histidyl modification to	Low	9	0.85	1.62	4.85E-02	603	JJJ3/DPH2/DPH1/DPH6
GO:0072329	monocarboxylic acid catabolic process	High	35	-0.65	-1.62	4.93E-02	1037	FOX2/CIT3/FDH1/POX1/PAH1/ICL2/FAA2/MDH3/TES1/PIP2/DCI1/SPS19/ECI1/MPO1/PXA2/IDP3/POT1/PXA1/YHR140W/GOR1

Supplementary Table S5.

Representative, statistically significant genes from the ranked differential expression analysis.

Gene	Phase	log2FC	padj	n_terms	Ontology	Interpratation
SOL4	High DO	-3.838	7.769E-23	1	BP	nicotinamide nucleotide metabolic process
IGD1	High DO	-3.267	6.491E-14	1	BP	glycogen biosynthetic process
DCS2	High DO	-2.651	4.926E-14	1	BP	deadenylation-dependent decapping of nuclear-transcribed mRNA
CTT1	High DO	-2.605	5.938E-08	2	BP	cellular oxidant detoxification; reactive oxygen species metabolic process
FMP45	High DO	-2.344	1.333E-13	1	BP	cortical actin cytoskeleton organization
HSP12	High DO	-2.119	3.253E-10	3	BP	cellular response to abiotic stimulus; cellular response to heat; cellular response to oxidative stress
HSP33	High DO	-1.877	2.711E-08	3	BP	aldehyde catabolic process; methylglyoxal metabolic process; response to methylglyoxal
SDH7	High DO	-0.883	1.881E-02	3	BP	glucose metabolic process; mitochondrial respiratory chain complex assembly; regulation of carbohydrate biosynthetic process
MUP1	Low DO	2.911	1.215E-20	1	BP	amino acid transmembrane transport
OPT1	Low DO	2.792	1.281E-13	1	BP	oligopeptide transmembrane transport
MET14	Low DO	2.727	3.601E-17	1	BP	hydrogen sulfide metabolic process
SPB1	Low DO	2.069	7.963E-11	2	BP	maturation of LSU-rRNA from tricistronic rRNA transcript (SSU-rRNA, 5.8S rRNA, LSU-rRNA); rRNA methylation
MTR4	Low DO	1.895	3.712E-08	2	BP	TRAMP-dependent tRNA surveillance pathway; nuclear polyadenylation-dependent rRNA catabolic process
ELP2	Low DO	1.569	9.390E-06	1	BP	tRNA wobble uridine modification
YEF3	Low DO	1.515	7.639E-05	1	BP	cytoplasmic translation
RPC40	Low DO	1.433	9.154E-05	2	BP	tRNA transcription; termination of RNA polymerase III transcription
LOC1	Low DO	1.160	3.297E-04	4	BP	endonucleolytic cleavage in 5'-ETS of tricistronic rRNA transcript (SSU-rRNA, 5.8S rRNA, LSU-rRNA); endonucleolytic cleavage in ITS1 to separate SSU-rRNA from 5.8S rRNA and LSU-rRNA from tricistronic rRNA transcript (SSU-rRNA, 5.8S rRNA, LSU-rRNA); endonucleolytic cleavage to generate mature 5'-end of SSU-rRNA from (SSU-rRNA, 5.8S rRNA, LSU-rRNA); [illegible]
PAU19	High DO	-3.178	1.847E-08	1	MF	structural constituent of cell wall
HTB1	High DO	-3.153	6.579E-22	2	MF	protein heterodimerization activity; structural constituent of chromatin
GPX1	High DO	-2.605	3.601E-17	2	MF	glutathione peroxidase activity; thioredoxin-dependent peroxiredoxin activity
TFS1	High DO	-2.555	1.503E-09	2	MF	endopeptidase inhibitor activity; lipid binding
MUP1	Low DO	2.911	1.215E-20	3	MF	neutral L-amino acid transmembrane transporter activity; organic cation transmembrane transporter activity; sulfur compound transmembrane transporter activity
DHR2	Low DO	2.587	4.780E-09	2	MF	ATP hydrolysis activity; RNA helicase activity
NOP7	Low DO	2.399	5.227E-08	3	MF	large ribosomal subunit rRNA binding; mRNA binding;
RRP5	Low DO	2.099	1.839E-09	3	MF	U3 snoRNA binding; mRNA binding; rRNA primary transcript binding
SPB1	Low DO	2.069	7.963E-11	1	MF	rRNA methyltransferase activity
TRM11	Low DO	1.945	2.491E-05	3	MF	N-methyltransferase activity; tRNA binding; tRNA methyltransferase activity
RPA19[illegible]	Low DO	1.840	2.602E-08	1	MF	DNA-directed RNA polymerase activity

Supplementary Table S4B.

Complete lists of genes contributing to the GO enrichment results.

Ontology	Phase	gene	n_terms	log2FC	DE_padj	representative_GO_terms
BP	High DO	TSA1	6	-1.074	1.373E-03	glucose metabolic process; cellular oxidant detoxification; cellular response to heat; cellular response to oxidative stress; reactive oxygen species metabolic process; regulation of carbohydrate biosynthetic process
BP	High DO	TSA2	4	-1.770	1.322E-08	cellular oxidant detoxification; cellular response to heat; cellular response to oxidative stress; reactive oxygen species metabolic process
BP	High DO	HSP31	4	-1.482	5.699E-06	cellular response to oxidative stress; aldehyde catabolic process; response to methylglyoxal; methylglyoxal metabolic process
BP	High DO	AHP1	3	-2.307	9.691E-06	cellular oxidant detoxification; cellular response to oxidative stress; reactive oxygen species metabolic process
BP	High DO	HSP12	3	-2.119	3.253E-10	cellular response to heat; cellular response to abiotic stimulus; cellular response to oxidative stress
BP	High DO	HSP33	3	-1.877	2.711E-08	aldehyde catabolic process; response to methylglyoxal; methylglyoxal metabolic process
BP	High DO	HSP32	3	-1.814	3.813E-07	aldehyde catabolic process; response to methylglyoxal; methylglyoxal metabolic process
BP	High DO	GLO1	3	-1.642	4.786E-08	aldehyde catabolic process; response to methylglyoxal; methylglyoxal metabolic process
BP	High DO	TDH3	3	-1.499	3.905E-03	glucose metabolic process; reactive oxygen species metabolic process; nicotinamide nucleotide metabolic process
BP	High DO	SNO4	3	-1.428	1.577E-05	aldehyde catabolic process; response to methylglyoxal; methylglyoxal metabolic process
BP	High DO	SOD2	3	-1.195	2.376E-04	cellular oxidant detoxification; cellular response to oxidative stress; reactive oxygen species metabolic process
BP	High DO	SOD1	3	-1.144	3.880E-04	cellular oxidant detoxification; cellular response to oxidative stress; reactive oxygen species metabolic process
BP	High DO	SDH7	3	-0.883	1.881E-02	glucose metabolic process; mitochondrial respiratory chain complex
BP	High DO	GIP2	2	-3.014	7.036E-10	glycogen biosynthetic process; regulation of carbohydrate
BP	High DO	PGM2	2	-2.762	1.893E-16	glycogen biosynthetic process; glucose metabolic process
BP	High DO	HXK1	2	-2.689	2.180E-08	glucose metabolic process; nicotinamide nucleotide metabolic process
BP	High DO	GPX1	2	-2.605	3.601E-17	cellular oxidant detoxification; cellular response to oxidative stress
BP	High DO	CTT1	2	-2.605	5.938E-08	cellular oxidant detoxification; reactive oxygen species metabolic
BP	High DO	DDR2	2	-2.400	5.695E-07	cellular response to heat; cellular response to oxidative stress
BP	High DO	SRX1	2	-2.387	3.049E-14	cellular oxidant detoxification; cellular response to oxidative stress
BP	High DO	GRE3	2	-2.028	4.267E-10	cellular response to abiotic stimulus; cellular response to oxidative stress
BP	High DO	XBP1	2	-1.916	1.415E-03	cellular response to heat; cellular response to abiotic stimulus
BP	High DO	CTA1	2	-1.903	3.485E-04	cellular oxidant detoxification; reactive oxygen species metabolic process
BP	High DO	TPS1	2	-1.892	1.962E-08	cellular response to heat; cellular response to abiotic stimulus
BP	High DO	GLK1	2	-1.779	3.752E-08	glucose metabolic process; nicotinamide nucleotide metabolic process
BP	High DO	NQM1	2	-1.688	2.017E-06	cellular response to oxidative stress; nicotinamide nucleotide metabolic process
BP	High DO	TDH1	2	-1.654	1.394E-05	glucose metabolic process; nicotinamide nucleotide metabolic process
BP	High DO	SDH8	2	-1.653	6.255E-05	mitochondrial respiratory chain complex assembly; cellular response to oxidative stress
BP	High DO	PIG2	2	-1.645	9.076E-05	glycogen biosynthetic process; regulation of carbohydrate biosynthetic process
BP	High DO	GRX2	2	-1.552	1.425E-06	cellular oxidant detoxification; cellular response to oxidative stress

BP	High DO	GPM2	2	-1.501	2.729E-05	glucose metabolic process; nicotinamide nucleotide metabolic process
BP	High DO	PRX1	2	-1.425	4.581E-06	cellular oxidant detoxification; cellular response to oxidative stress
BP	High DO	NGK1	2	-1.420	1.617E-04	glucose metabolic process; nicotinamide nucleotide metabolic process
BP	High DO	GAC1	2	-1.361	4.393E-03	glycogen biosynthetic process; regulation of carbohydrate biosynthetic process
BP	High DO	ZWF1	2	-1.344	1.425E-03	glucose metabolic process; nicotinamide nucleotide metabolic process
BP	High DO	GRX1	2	-1.218	8.773E-05	cellular oxidant detoxification; cellular response to oxidative stress
BP	High DO	PSK1	2	-1.203	6.690E-04	glycogen biosynthetic process; regulation of carbohydrate biosynthetic process
BP	High DO	EMI2	2	-1.178	5.973E-04	glucose metabolic process; nicotinamide nucleotide metabolic process
BP	High DO	PGM1	2	-1.033	9.597E-03	glycogen biosynthetic process; glucose metabolic process
BP	High DO	GID7	2	-0.992	2.258E-02	glucose metabolic process; regulation of carbohydrate biosynthetic process
BP	High DO	HYR1	2	-0.989	3.142E-03	cellular oxidant detoxification; cellular response to oxidative stress
BP	High DO	VID30	2	-0.971	1.101E-02	glucose metabolic process; regulation of carbohydrate biosynthetic process
BP	High DO	GID10	2	-0.804	6.480E-02	glucose metabolic process; regulation of carbohydrate biosynthetic process
BP	High DO	VID28	2	-0.776	8.841E-02	glucose metabolic process; regulation of carbohydrate biosynthetic process
BP	High DO	PIB1	2	-0.741	4.073E-02	glucose metabolic process; regulation of carbohydrate biosynthetic process
BP	High DO	FYV10	2	-0.643	1.314E-01	glucose metabolic process; regulation of carbohydrate biosynthetic process
BP	High DO	CAT8	2	-0.588	1.270E-01	glucose metabolic process; regulation of carbohydrate biosynthetic process
BP	High DO	RMD5	2	-0.568	1.772E-01	glucose metabolic process; regulation of carbohydrate biosynthetic process
BP	High DO	SOL4	1	-3.838	7.769E-23	nicotinamide nucleotide metabolic process
BP	High DO	IGD1	1	-3.267	6.491E-14	glycogen biosynthetic process
BP	High DO	DCS2	1	-2.651	4.926E-14	deadenylation-dependent decapping of nuclear-transcribed mRNA
BP	High DO	STF2	1	-2.533	4.874E-14	cellular response to abiotic stimulus
BP	High DO	TKL2	1	-2.475	3.253E-07	nicotinamide nucleotide metabolic process
BP	High DO	GND2	1	-2.432	7.967E-08	nicotinamide nucleotide metabolic process
BP	High DO	GSY1	1	-2.423	6.353E-10	glycogen biosynthetic process
BP	High DO	ERR2	1	-2.422	4.039E-02	nicotinamide nucleotide metabolic process
BP	High DO	FMP45	1	-2.344	1.333E-13	cortical actin cytoskeleton organization
BP	High DO	ECM4	1	-2.283	7.838E-05	cellular oxidant detoxification
BP	High DO	NDE2	1	-2.236	1.445E-04	glucose metabolic process
BP	High DO	YNL194C	1	-2.179	7.600E-09	cortical actin cytoskeleton organization
BP	High DO	ROQ1	1	-2.153	8.062E-12	cellular response to abiotic stimulus
BP	High DO	GAD1	1	-2.137	7.854E-09	cellular response to oxidative stress
BP	High DO	GDB1	1	-2.102	2.274E-07	glycogen biosynthetic process
BP	High DO	GLC3	1	-1.999	8.872E-06	glycogen biosynthetic process
BP	High DO	UGP1	1	-1.997	1.693E-09	glycogen biosynthetic process
BP	High DO	HSP104	1	-1.934	2.028E-06	cellular response to heat
BP	High DO	HSP26	1	-1.917	6.019E-06	cellular response to heat
BP	High DO	SDH5	1	-1.902	1.784E-08	mitochondrial respiratory chain complex assembly
BP	High DO	ERR3	1	-1.848	6.491E-07	nicotinamide nucleotide metabolic process
BP	High DO	GSY2	1	-1.846	1.027E-05	glycogen biosynthetic process
BP	High DO	NNR1	1	-1.837	3.241E-08	nicotinamide nucleotide metabolic process
BP	High DO	YJR096W	1	-1.829	1.432E-08	cellular response to oxidative stress
BP	High DO	YDL124W	1	-1.779	1.073E-06	cellular response to oxidative stress
BP	High DO	SSC1	1	-1.760	8.579E-06	mitochondrial respiratory chain complex assembly

BP	High DO	USV1	1	-1.741	1.145E-05	cellular response to abiotic stimulus
BP	High DO	SDH6	1	-1.735	2.063E-08	mitochondrial respiratory chain complex assembly
BP	High DO	HSP82	1	-1.733	1.957E-06	cellular response to heat
BP	High DO	GPD1	1	-1.727	4.281E-06	cellular response to abiotic stimulus
BP	High DO	EDC1	1	-1.689	5.080E-06	deadenylation-dependent decapping of nuclear-transcribed mRNA
BP	High DO	GOR1	1	-1.666	1.171E-06	aldehyde catabolic process
BP	High DO	YNL190W	1	-1.649	1.620E-03	cellular response to abiotic stimulus
BP	High DO	GCY1	1	-1.629	8.033E-07	cellular response to oxidative stress
BP	High DO	MDH2	1	-1.597	2.865E-04	glucose metabolic process
BP	High DO	IDP3	1	-1.592	2.876E-04	nicotinamide nucleotide metabolic process
BP	High DO	PDC1	1	-1.566	4.782E-04	glucose metabolic process
BP	High DO	SIP18	1	-1.557	2.235E-05	cellular response to abiotic stimulus
BP	High DO	FRT2	1	-1.500	2.854E-03	cellular response to abiotic stimulus
BP	High DO	NCE103	1	-1.450	5.455E-06	cellular response to oxidative stress
BP	High DO	SSA1	1	-1.404	2.486E-04	cellular response to heat
BP	High DO	ABP1	1	-1.403	1.254E-02	cortical actin cytoskeleton organization
BP	High DO	GUT2	1	-1.388	9.516E-04	nicotinamide nucleotide metabolic process
BP	High DO	NNR2	1	-1.378	2.824E-05	nicotinamide nucleotide metabolic process
BP	High DO	HFD1	1	-1.360	1.476E-05	aldehyde catabolic process
BP	High DO	COA4	1	-1.347	4.601E-03	mitochondrial respiratory chain complex assembly
BP	High DO	EDC2	1	-1.332	5.968E-05	deadenylation-dependent decapping of nuclear-transcribed mRNA
BP	High DO	COX14	1	-1.331	2.263E-05	mitochondrial respiratory chain complex assembly
BP	High DO	HSP78	1	-1.309	3.379E-04	cellular response to heat
BP	High DO	CBP6	1	-1.299	3.496E-05	mitochondrial respiratory chain complex assembly
BP	High DO	GLG1	1	-1.288	1.127E-03	glycogen biosynthetic process
BP	High DO	PET117	1	-1.284	2.000E-03	mitochondrial respiratory chain complex assembly
BP	High DO	MSS51	1	-1.238	6.414E-03	mitochondrial respiratory chain complex assembly
BP	High DO	COX19	1	-1.236	6.290E-03	mitochondrial respiratory chain complex assembly
BP	High DO	CBP4	1	-1.233	4.512E-04	mitochondrial respiratory chain complex assembly
BP	High DO	DCS1	1	-1.204	1.876E-04	deadenylation-dependent decapping of nuclear-transcribed mRNA
BP	High DO	GTT1	1	-1.185	4.721E-04	cellular oxidant detoxification
BP	High DO	ACT1	1	-1.184	1.007E-02	cortical actin cytoskeleton organization
BP	High DO	SSK1	1	-1.161	2.463E-03	cellular response to abiotic stimulus
BP	High DO	SOL1	1	-1.143	6.082E-04	nicotinamide nucleotide metabolic process
BP	High DO	MCR1	1	-1.089	5.799E-04	cellular response to oxidative stress
BP	High DO	COA6	1	-1.069	1.064E-03	mitochondrial respiratory chain complex assembly
BP	High DO	SSN8	1	-1.053	3.292E-03	cellular response to heat
BP	High DO	GLG2	1	-1.027	2.644E-03	glycogen biosynthetic process
BP	High DO	ARC18	1	-1.020	5.120E-03	cortical actin cytoskeleton organization
BP	High DO	COA3	1	-1.012	3.844E-03	mitochondrial respiratory chain complex assembly
BP	High DO	ALD4	1	-1.003	4.923E-02	nicotinamide nucleotide metabolic process
BP	High DO	PAN1	1	-0.994	1.094E-01	cortical actin cytoskeleton organization
BP	High DO	ADH1	1	-0.988	7.928E-03	glucose metabolic process
BP	High DO	YLR345W	1	-0.985	2.775E-03	nicotinamide nucleotide metabolic process
BP	High DO	ENT2	1	-0.984	1.987E-02	cortical actin cytoskeleton organization
BP	High DO	PET191	1	-0.976	5.494E-03	mitochondrial respiratory chain complex assembly
BP	High DO	COX17	1	-0.962	8.649E-03	mitochondrial respiratory chain complex assembly
BP	High DO	SPR28	1	-0.931	5.857E-03	cortical actin cytoskeleton organization
BP	High DO	SIS1	1	-0.922	6.773E-03	cellular response to heat
BP	High DO	EDE1	1	-0.920	5.249E-02	cortical actin cytoskeleton organization
BP	High DO	YMR315V	1	-0.910	4.451E-02	nicotinamide nucleotide metabolic process
BP	High DO	PNP1	1	-0.910	7.280E-03	nicotinamide nucleotide metabolic process
BP	High DO	SOL2	1	-0.905	1.390E-02	nicotinamide nucleotide metabolic process
BP	High DO	YJL068C	1	-0.902	6.182E-03	aldehyde catabolic process
BP	High DO	ARP3	1	-0.896	4.253E-02	cortical actin cytoskeleton organization
BP	High DO	SCO1	1	-0.874	1.298E-02	mitochondrial respiratory chain complex assembly
BP	High DO	PKP1	1	-0.871	2.101E-02	glucose metabolic process
BP	High DO	SUR7	1	-0.833	4.336E-02	cortical actin cytoskeleton organization
BP	High DO	CBP3	1	-0.831	4.648E-02	mitochondrial respiratory chain complex assembly
BP	High DO	ARC15	1	-0.824	2.208E-02	cortical actin cytoskeleton organization
BP	High DO	MDM35	1	-0.809	4.554E-02	mitochondrial respiratory chain complex assembly

BP	High DO	RCF1	1	-0.802	2.346E-02	mitochondrial respiratory chain complex assembly
BP	High DO	ENO1	1	-0.799	6.187E-02	nicotinamide nucleotide metabolic process
BP	High DO	RGT1	1	-0.778	7.908E-02	glucose metabolic process
BP	High DO	CMC1	1	-0.776	5.208E-02	mitochondrial respiratory chain complex assembly
BP	High DO	RVS167	1	-0.767	5.726E-02	cortical actin cytoskeleton organization
BP	High DO	LPD1	1	-0.767	6.308E-02	reactive oxygen species metabolic process
BP	High DO	RPE1	1	-0.763	6.053E-02	nicotinamide nucleotide metabolic process
BP	High DO	SHY1	1	-0.761	4.350E-02	mitochondrial respiratory chain complex assembly
BP	High DO	AKL1	1	-0.745	5.035E-02	cortical actin cytoskeleton organization
BP	High DO	MDH3	1	-0.717	4.360E-02	nicotinamide nucleotide metabolic process
BP	High DO	TRR2	1	-0.712	6.859E-02	reactive oxygen species metabolic process
BP	High DO	COX23	1	-0.698	4.892E-02	mitochondrial respiratory chain complex assembly
BP	High DO	ARC40	1	-0.698	6.819E-02	cortical actin cytoskeleton organization
BP	High DO	LDB17	1	-0.680	1.332E-01	cortical actin cytoskeleton organization
BP	High DO	PCL6	1	-0.674	1.275E-01	regulation of carbohydrate biosynthetic process
BP	High DO	CCP1	1	-0.661	7.799E-02	reactive oxygen species metabolic process
BP	High DO	TWF1	1	-0.660	8.050E-02	cortical actin cytoskeleton organization
BP	High DO	PRK1	1	-0.659	1.731E-01	cortical actin cytoskeleton organization
BP	High DO	AIM44	1	-0.631	2.653E-01	cortical actin cytoskeleton organization
BP	High DO	ZIP1	1	-0.605	1.108E-01	cortical actin cytoskeleton organization
BP	High DO	SOM1	1	-0.599	1.431E-01	mitochondrial respiratory chain complex assembly
BP	High DO	COA1	1	-0.588	1.270E-01	mitochondrial respiratory chain complex assembly
BP	High DO	COA2	1	-0.560	2.462E-01	mitochondrial respiratory chain complex assembly
BP	High DO	ARC19	1	-0.543	1.388E-01	cortical actin cytoskeleton organization
BP	High DO	PKP2	1	-0.529	1.738E-01	glucose metabolic process
BP	High DO	TAZ1	1	-0.526	1.661E-01	mitochondrial respiratory chain complex assembly
BP	High DO	PCL8	1	-0.524	1.829E-01	regulation of carbohydrate biosynthetic process
BP	High DO	FBP26	1	-0.518	2.233E-01	glucose metabolic process
BP	High DO	SCO2	1	-0.515	1.805E-01	mitochondrial respiratory chain complex assembly
BP	High DO	MSS4	1	-0.510	2.678E-01	cortical actin cytoskeleton organization
BP	High DO	COX16	1	-0.510	2.253E-01	mitochondrial respiratory chain complex assembly
BP	High DO	GPM1	1	-0.508	2.074E-01	glucose metabolic process
BP	High DO	SLA1	1	-0.507	1.909E-01	cortical actin cytoskeleton organization
BP	High DO	CDC11	1	-0.499	2.338E-01	cortical actin cytoskeleton organization
BP	High DO	COI1	1	-0.497	2.544E-01	mitochondrial respiratory chain complex assembly
BP	High DO	TPI1	1	-0.467	2.190E-01	glucose metabolic process
BP	High DO	CDH1	1	-0.462	2.707E-01	cortical actin cytoskeleton organization
BP	Low DO	RRP5	4	2.099	1.839E-09	maturation of LSU-rRNA from tricistronic rRNA transcript (SSU-rRNA, 5.8S rRNA, LSU-rRNA); endonucleolytic cleavage in ITS1 to separate SSU-rRNA from 5.8S rRNA and LSU-rRNA from tricistronic rRNA transcript (SSU-rRNA, 5.8S rRNA, LSU-rRNA); endonucleolytic cleavage to generate mature 5'-end of SSU-rRNA from (SSU-rRNA, 5.8S rRNA, LSU-rRNA); endonucleolytic cleavage in 5'-ETS of tricistronic rRNA transcript (SSU-rRNA, 5.8S rRNA, LSU-rRNA)
BP	Low DO	LOC1	4	1.160	3.297E-04	endonucleolytic cleavage in ITS1 to separate SSU-rRNA from 5.8S rRNA and LSU-rRNA from tricistronic rRNA transcript (SSU-rRNA, 5.8S rRNA, LSU-rRNA); endonucleolytic cleavage to generate mature 5'-end of SSU-rRNA from (SSU-rRNA, 5.8S rRNA, LSU-rRNA); endonucleolytic cleavage in 5'-ETS of tricistronic rRNA transcript (SSU-rRNA, 5.8S rRNA, LSU-rRNA); ribosomal large subunit export from nucleus
BP	Low DO	EMG1	4	1.098	3.585E-03	endonucleolytic cleavage in ITS1 to separate SSU-rRNA from 5.8S rRNA and LSU-rRNA from tricistronic rRNA transcript (SSU-rRNA, 5.8S rRNA, LSU-rRNA); endonucleolytic cleavage to generate mature 5'-end of SSU-rRNA from (SSU-rRNA, 5.8S rRNA, LSU-rRNA); endonucleolytic cleavage in 5'-ETS of tricistronic rRNA transcript (SSU-rRNA, 5.8S rRNA, LSU-rRNA); rRNA methylation

BP	Low DO	NOP58	4	1.080	1.167E-03	endonucleolytic cleavage in ITS1 to separate SSU-rRNA from 5.8S rRNA and LSU-rRNA from tricistronic rRNA transcript (SSU-rRNA, 5.8S rRNA, LSU-rRNA); endonucleolytic cleavage to generate mature 5'-end of SSU-rRNA from (SSU-rRNA, 5.8S rRNA, LSU-rRNA); endonucleolytic cleavage in 5'-ETS of tricistronic rRNA transcript (SSU-rRNA, 5.8S rRNA, LSU-rRNA); rRNA methylation
BP	Low DO	NOP14	3	2.108	3.172E-08	endonucleolytic cleavage in ITS1 to separate SSU-rRNA from 5.8S rRNA and LSU-rRNA from tricistronic rRNA transcript (SSU-rRNA, 5.8S rRNA, LSU-rRNA); endonucleolytic cleavage to generate mature 5'-end of SSU-rRNA from (SSU-rRNA, 5.8S rRNA, LSU-rRNA); endonucleolytic cleavage in 5'-ETS of tricistronic rRNA transcript (SSU-rRNA, 5.8S rRNA, LSU-rRNA)
BP	Low DO	UTP10	3	2.092	1.431E-12	endonucleolytic cleavage in ITS1 to separate SSU-rRNA from 5.8S rRNA and LSU-rRNA from tricistronic rRNA transcript (SSU-rRNA, 5.8S rRNA, LSU-rRNA); endonucleolytic cleavage to generate mature 5'-end of SSU-rRNA from (SSU-rRNA, 5.8S rRNA, LSU-rRNA); endonucleolytic cleavage in 5'-ETS of tricistronic rRNA transcript (SSU-rRNA, 5.8S rRNA, LSU-rRNA)
BP	Low DO	MRD1	3	2.024	7.505E-07	endonucleolytic cleavage in ITS1 to separate SSU-rRNA from 5.8S rRNA and LSU-rRNA from tricistronic rRNA transcript (SSU-rRNA, 5.8S rRNA, LSU-rRNA); endonucleolytic cleavage to generate mature 5'-end of SSU-rRNA from (SSU-rRNA, 5.8S rRNA, LSU-rRNA); endonucleolytic cleavage in 5'-ETS of tricistronic rRNA transcript (SSU-rRNA, 5.8S rRNA, LSU-rRNA)
BP	Low DO	UTP14	3	2.002	1.133E-08	endonucleolytic cleavage in ITS1 to separate SSU-rRNA from 5.8S rRNA and LSU-rRNA from tricistronic rRNA transcript (SSU-rRNA, 5.8S rRNA, LSU-rRNA); endonucleolytic cleavage to generate mature 5'-end of SSU-rRNA from (SSU-rRNA, 5.8S rRNA, LSU-rRNA); endonucleolytic cleavage in 5'-ETS of tricistronic rRNA transcript (SSU-rRNA, 5.8S rRNA, LSU-rRNA)
BP	Low DO	SAS10	3	1.934	4.577E-08	endonucleolytic cleavage in ITS1 to separate SSU-rRNA from 5.8S rRNA and LSU-rRNA from tricistronic rRNA transcript (SSU-rRNA, 5.8S rRNA, LSU-rRNA); endonucleolytic cleavage to generate mature 5'-end of SSU-rRNA from (SSU-rRNA, 5.8S rRNA, LSU-rRNA); endonucleolytic cleavage in 5'-ETS of tricistronic rRNA transcript (SSU-rRNA, 5.8S rRNA, LSU-rRNA)
BP	Low DO	UTP20	3	1.894	1.149E-08	endonucleolytic cleavage in ITS1 to separate SSU-rRNA from 5.8S rRNA and LSU-rRNA from tricistronic rRNA transcript (SSU-rRNA, 5.8S rRNA, LSU-rRNA); endonucleolytic cleavage to generate mature 5'-end of SSU-rRNA from (SSU-rRNA, 5.8S rRNA, LSU-rRNA); endonucleolytic cleavage in 5'-ETS of tricistronic rRNA transcript (SSU-rRNA, 5.8S rRNA, LSU-rRNA)
BP	Low DO	UTP23	3	1.859	5.080E-06	endonucleolytic cleavage in ITS1 to separate SSU-rRNA from 5.8S rRNA and LSU-rRNA from tricistronic rRNA transcript (SSU-rRNA, 5.8S rRNA, LSU-rRNA); endonucleolytic cleavage to generate mature 5'-end of SSU-rRNA from (SSU-rRNA, 5.8S rRNA, LSU-rRNA); endonucleolytic cleavage in 5'-ETS of tricistronic rRNA transcript (SSU-rRNA, 5.8S rRNA, LSU-rRNA)
BP	Low DO	ROK1	3	1.654	9.307E-07	endonucleolytic cleavage in ITS1 to separate SSU-rRNA from 5.8S rRNA and LSU-rRNA from tricistronic rRNA transcript (SSU-rRNA, 5.8S rRNA, LSU-rRNA); endonucleolytic cleavage to generate mature 5'-end of SSU-rRNA from (SSU-rRNA, 5.8S rRNA, LSU-rRNA); endonucleolytic cleavage in 5'-ETS of tricistronic rRNA transcript (SSU-rRNA, 5.8S rRNA, LSU-rRNA)
BP	Low DO	PWP2	3	1.632	4.779E-06	endonucleolytic cleavage in ITS1 to separate SSU-rRNA from 5.8S rRNA and LSU-rRNA from tricistronic rRNA transcript (SSU-rRNA, 5.8S rRNA, LSU-rRNA); endonucleolytic cleavage to generate mature 5'-end of SSU-rRNA from (SSU-rRNA, 5.8S rRNA, LSU-rRNA); endonucleolytic cleavage in 5'-ETS of tricistronic rRNA transcript (SSU-rRNA, 5.8S rRNA, LSU-rRNA)

BP	Low DO	DIP2	3	1.593	7.082E-06	endonucleolytic cleavage in ITS1 to separate SSU-rRNA from 5.8S rRNA and LSU-rRNA from tricistronic rRNA transcript (SSU-rRNA, 5.8S rRNA, LSU-rRNA); endonucleolytic cleavage to generate mature 5'-end of SSU-rRNA from (SSU-rRNA, 5.8S rRNA, LSU-rRNA); endonucleolytic cleavage in 5'-ETS of tricistronic rRNA transcript (SSU-rRNA, 5.8S rRNA, LSU-rRNA)
BP	Low DO	ESF2	3	1.578	1.596E-05	endonucleolytic cleavage in ITS1 to separate SSU-rRNA from 5.8S rRNA and LSU-rRNA from tricistronic rRNA transcript (SSU-rRNA, 5.8S rRNA, LSU-rRNA); endonucleolytic cleavage to generate mature 5'-end of SSU-rRNA from (SSU-rRNA, 5.8S rRNA, LSU-rRNA); endonucleolytic cleavage in 5'-ETS of tricistronic rRNA transcript (SSU-rRNA, 5.8S rRNA, LSU-rRNA)
BP	Low DO	UTP11	3	1.520	3.432E-04	endonucleolytic cleavage in ITS1 to separate SSU-rRNA from 5.8S rRNA and LSU-rRNA from tricistronic rRNA transcript (SSU-rRNA, 5.8S rRNA, LSU-rRNA); endonucleolytic cleavage to generate mature 5'-end of SSU-rRNA from (SSU-rRNA, 5.8S rRNA, LSU-rRNA); endonucleolytic cleavage in 5'-ETS of tricistronic rRNA transcript (SSU-rRNA, 5.8S rRNA, LSU-rRNA)
BP	Low DO	MPP10	3	1.453	4.861E-05	endonucleolytic cleavage in ITS1 to separate SSU-rRNA from 5.8S rRNA and LSU-rRNA from tricistronic rRNA transcript (SSU-rRNA, 5.8S rRNA, LSU-rRNA); endonucleolytic cleavage to generate mature 5'-end of SSU-rRNA from (SSU-rRNA, 5.8S rRNA, LSU-rRNA); endonucleolytic cleavage in 5'-ETS of tricistronic rRNA transcript (SSU-rRNA, 5.8S rRNA, LSU-rRNA)
BP	Low DO	UTP7	3	1.425	5.419E-05	endonucleolytic cleavage in ITS1 to separate SSU-rRNA from 5.8S rRNA and LSU-rRNA from tricistronic rRNA transcript (SSU-rRNA, 5.8S rRNA, LSU-rRNA); endonucleolytic cleavage to generate mature 5'-end of SSU-rRNA from (SSU-rRNA, 5.8S rRNA, LSU-rRNA); endonucleolytic cleavage in 5'-ETS of tricistronic rRNA transcript (SSU-rRNA, 5.8S rRNA, LSU-rRNA)
BP	Low DO	UTP13	3	1.413	8.555E-06	endonucleolytic cleavage in ITS1 to separate SSU-rRNA from 5.8S rRNA and LSU-rRNA from tricistronic rRNA transcript (SSU-rRNA, 5.8S rRNA, LSU-rRNA); endonucleolytic cleavage to generate mature 5'-end of SSU-rRNA from (SSU-rRNA, 5.8S rRNA, LSU-rRNA); endonucleolytic cleavage in 5'-ETS of tricistronic rRNA transcript (SSU-rRNA, 5.8S rRNA, LSU-rRNA)
BP	Low DO	NOP9	3	1.411	2.572E-05	endonucleolytic cleavage in ITS1 to separate SSU-rRNA from 5.8S rRNA and LSU-rRNA from tricistronic rRNA transcript (SSU-rRNA, 5.8S rRNA, LSU-rRNA); endonucleolytic cleavage to generate mature 5'-end of SSU-rRNA from (SSU-rRNA, 5.8S rRNA, LSU-rRNA); endonucleolytic cleavage in 5'-ETS of tricistronic rRNA transcript (SSU-rRNA, 5.8S rRNA, LSU-rRNA)
BP	Low DO	UTP6	3	1.336	1.065E-03	endonucleolytic cleavage in ITS1 to separate SSU-rRNA from 5.8S rRNA and LSU-rRNA from tricistronic rRNA transcript (SSU-rRNA, 5.8S rRNA, LSU-rRNA); endonucleolytic cleavage to generate mature 5'-end of SSU-rRNA from (SSU-rRNA, 5.8S rRNA, LSU-rRNA); endonucleolytic cleavage in 5'-ETS of tricistronic rRNA transcript (SSU-rRNA, 5.8S rRNA, LSU-rRNA)
BP	Low DO	RCL1	3	1.224	1.435E-03	endonucleolytic cleavage in ITS1 to separate SSU-rRNA from 5.8S rRNA and LSU-rRNA from tricistronic rRNA transcript (SSU-rRNA, 5.8S rRNA, LSU-rRNA); endonucleolytic cleavage to generate mature 5'-end of SSU-rRNA from (SSU-rRNA, 5.8S rRNA, LSU-rRNA); endonucleolytic cleavage in 5'-ETS of tricistronic rRNA transcript (SSU-rRNA, 5.8S rRNA, LSU-rRNA)
BP	Low DO	DBP8	3	1.197	4.751E-04	endonucleolytic cleavage in ITS1 to separate SSU-rRNA from 5.8S rRNA and LSU-rRNA from tricistronic rRNA transcript (SSU-rRNA, 5.8S rRNA, LSU-rRNA); endonucleolytic cleavage to generate mature 5'-end of SSU-rRNA from (SSU-rRNA, 5.8S rRNA, LSU-rRNA); endonucleolytic cleavage in 5'-ETS of tricistronic rRNA transcript (SSU-rRNA, 5.8S rRNA, LSU-rRNA)

BP	Low DO	UTP18	3	1.190	5.671E-04	endonucleolytic cleavage in ITS1 to separate SSU-rRNA from 5.8S rRNA and LSU-rRNA from tricistronic rRNA transcript (SSU-rRNA, 5.8S rRNA, LSU-rRNA); endonucleolytic cleavage to generate mature 5'-end of SSU-rRNA from (SSU-rRNA, 5.8S rRNA, LSU-rRNA); endonucleolytic cleavage in 5'-ETS of tricistronic rRNA transcript (SSU-rRNA, 5.8S rRNA, LSU-rRNA)
BP	Low DO	FCF1	3	1.071	7.405E-03	endonucleolytic cleavage in ITS1 to separate SSU-rRNA from 5.8S rRNA and LSU-rRNA from tricistronic rRNA transcript (SSU-rRNA, 5.8S rRNA, LSU-rRNA); endonucleolytic cleavage to generate mature 5'-end of SSU-rRNA from (SSU-rRNA, 5.8S rRNA, LSU-rRNA); endonucleolytic cleavage in 5'-ETS of tricistronic rRNA transcript (SSU-rRNA, 5.8S rRNA, LSU-rRNA)
BP	Low DO	FCF2	3	1.019	7.575E-03	endonucleolytic cleavage in ITS1 to separate SSU-rRNA from 5.8S rRNA and LSU-rRNA from tricistronic rRNA transcript (SSU-rRNA, 5.8S rRNA, LSU-rRNA); endonucleolytic cleavage to generate mature 5'-end of SSU-rRNA from (SSU-rRNA, 5.8S rRNA, LSU-rRNA); endonucleolytic cleavage in 5'-ETS of tricistronic rRNA transcript (SSU-rRNA, 5.8S rRNA, LSU-rRNA)
BP	Low DO	SPB1	2	2.069	7.963E-11	maturation of LSU-rRNA from tricistronic rRNA transcript (SSU-rRNA, 5.8S rRNA, LSU-rRNA); rRNA methylation
BP	Low DO	BMS1	2	1.999	3.991E-09	endonucleolytic cleavage to generate mature 5'-end of SSU-rRNA from (SSU-rRNA, 5.8S rRNA, LSU-rRNA); endonucleolytic cleavage in 5'-ETS of tricistronic rRNA transcript (SSU-rRNA, 5.8S rRNA, LSU-rRNA)
BP	Low DO	NUG1	2	1.904	1.790E-07	maturation of LSU-rRNA from tricistronic rRNA transcript (SSU-rRNA, 5.8S rRNA, LSU-rRNA); ribosomal large subunit export from nucleus
BP	Low DO	MTR4	2	1.895	3.712E-08	nuclear polyadenylation-dependent rRNA catabolic process; TRAMP-dependent tRNA surveillance pathway
BP	Low DO	RPF1	2	1.876	2.548E-05	maturation of LSU-rRNA from tricistronic rRNA transcript (SSU-rRNA, 5.8S rRNA, LSU-rRNA); ribosomal large subunit export from nucleus
BP	Low DO	BUD23	2	1.483	1.730E-05	endonucleolytic cleavage in ITS1 to separate SSU-rRNA from 5.8S rRNA and LSU-rRNA from tricistronic rRNA transcript (SSU-rRNA, 5.8S rRNA, LSU-rRNA); rRNA methylation
BP	Low DO	PNO1	2	1.474	7.848E-05	endonucleolytic cleavage in ITS1 to separate SSU-rRNA from 5.8S rRNA and LSU-rRNA from tricistronic rRNA transcript (SSU-rRNA, 5.8S rRNA, LSU-rRNA); endonucleolytic cleavage to generate mature 5'-end of SSU-rRNA from (SSU-rRNA, 5.8S rRNA, LSU-rRNA)
BP	Low DO	RPC40	2	1.433	9.154E-05	tRNA transcription; termination of RNA polymerase III transcription
BP	Low DO	BUD27	2	1.333	1.577E-05	cytoplasmic translation; tRNA transcription
BP	Low DO	RRS1	2	1.320	3.880E-04	endonucleolytic cleavage in ITS1 to separate SSU-rRNA from 5.8S rRNA and LSU-rRNA from tricistronic rRNA transcript (SSU-rRNA, 5.8S rRNA, LSU-rRNA); ribosomal large subunit export from nucleus
BP	Low DO	BUD21	2	1.310	5.550E-04	endonucleolytic cleavage in ITS1 to separate SSU-rRNA from 5.8S rRNA and LSU-rRNA from tricistronic rRNA transcript (SSU-rRNA, 5.8S rRNA, LSU-rRNA); endonucleolytic cleavage to generate mature 5'-end of SSU-rRNA from (SSU-rRNA, 5.8S rRNA, LSU-rRNA)
BP	Low DO	RET1	2	1.308	2.390E-05	tRNA transcription; termination of RNA polymerase III transcription
BP	Low DO	RPC19	2	1.262	8.796E-04	tRNA transcription; termination of RNA polymerase III transcription
BP	Low DO	RPC25	2	1.212	2.503E-03	tRNA transcription; termination of RNA polymerase III transcription
BP	Low DO	RPC17	2	1.203	2.295E-03	tRNA transcription; termination of RNA polymerase III transcription

BP	Low DO	NOP53	2	1.198	6.688E-04	maturation of LSU-rRNA from tricistronic rRNA transcript (SSU-rRNA, 5.8S rRNA, LSU-rRNA); ribosomal large subunit export from nucleus
BP	Low DO	LCP5	2	1.178	1.437E-03	endonucleolytic cleavage in ITS1 to separate SSU-rRNA from 5.8S rRNA and LSU-rRNA from tricistronic rRNA transcript (SSU-rRNA, 5.8S rRNA, LSU-rRNA); endonucleolytic cleavage in 5'-ETS of tricistronic rRNA transcript (SSU-rRNA, 5.8S rRNA, LSU-rRNA)
BP	Low DO	RRP6	2	1.149	3.084E-03	nuclear polyadenylation-dependent rRNA catabolic process; TRAMP-dependent tRNA surveillance pathway
BP	Low DO	TRF5	2	1.125	5.734E-04	nuclear polyadenylation-dependent rRNA catabolic process; TRAMP-dependent tRNA surveillance pathway
BP	Low DO	RRP4	2	1.104	8.986E-03	nuclear polyadenylation-dependent rRNA catabolic process; TRAMP-dependent tRNA surveillance pathway
BP	Low DO	NOP2	2	1.092	2.737E-03	maturation of LSU-rRNA from tricistronic rRNA transcript (SSU-rRNA, 5.8S rRNA, LSU-rRNA); rRNA methylation
BP	Low DO	RPC53	2	1.078	2.016E-03	tRNA transcription; termination of RNA polymerase III transcription
BP	Low DO	RPC31	2	1.066	3.561E-03	tRNA transcription; termination of RNA polymerase III transcription
BP	Low DO	RAI1	2	1.034	1.062E-02	maturation of LSU-rRNA from tricistronic rRNA transcript (SSU-rRNA, 5.8S rRNA, LSU-rRNA); nuclear polyadenylation-dependent rRNA catabolic process
BP	Low DO	RPO26	2	1.031	6.680E-03	tRNA transcription; termination of RNA polymerase III transcription
BP	Low DO	RPC34	2	0.998	5.678E-03	tRNA transcription; termination of RNA polymerase III transcription
BP	Low DO	RPC37	2	0.972	6.836E-03	tRNA transcription; termination of RNA polymerase III transcription
BP	Low DO	MTR3	2	0.952	5.328E-03	nuclear polyadenylation-dependent rRNA catabolic process; TRAMP-dependent tRNA surveillance pathway
BP	Low DO	RPC82	2	0.933	9.403E-03	tRNA transcription; termination of RNA polymerase III transcription
BP	Low DO	RPB5	2	0.927	1.001E-02	tRNA transcription; termination of RNA polymerase III transcription
BP	Low DO	RPC11	2	0.924	1.472E-02	tRNA transcription; termination of RNA polymerase III transcription
BP	Low DO	DIS3	2	0.910	5.619E-03	nuclear polyadenylation-dependent rRNA catabolic process; TRAMP-dependent tRNA surveillance pathway
BP	Low DO	AIR1	2	0.878	1.350E-02	nuclear polyadenylation-dependent rRNA catabolic process; TRAMP-dependent tRNA surveillance pathway
BP	Low DO	RAT1	2	0.844	1.062E-02	maturation of LSU-rRNA from tricistronic rRNA transcript (SSU-rRNA, 5.8S rRNA, LSU-rRNA); nuclear polyadenylation-dependent rRNA catabolic process
BP	Low DO	RPC10	2	0.839	2.849E-02	tRNA transcription; termination of RNA polymerase III transcription
BP	Low DO	RRP45	2	0.834	3.735E-02	nuclear polyadenylation-dependent rRNA catabolic process; TRAMP-dependent tRNA surveillance pathway
BP	Low DO	RPO31	2	0.807	2.300E-02	tRNA transcription; termination of RNA polymerase III transcription
BP	Low DO	CSL4	2	0.775	5.629E-02	nuclear polyadenylation-dependent rRNA catabolic process; TRAMP-dependent tRNA surveillance pathway
BP	Low DO	RRP42	2	0.758	4.820E-02	nuclear polyadenylation-dependent rRNA catabolic process; TRAMP-dependent tRNA surveillance pathway
BP	Low DO	RRP46	2	0.733	7.096E-02	nuclear polyadenylation-dependent rRNA catabolic process; TRAMP-dependent tRNA surveillance pathway
BP	Low DO	RRP43	2	0.727	6.221E-02	nuclear polyadenylation-dependent rRNA catabolic process; TRAMP-dependent tRNA surveillance pathway
BP	Low DO	RPL1A	2	0.647	1.332E-01	ribosomal large subunit export from nucleus; cytoplasmic translation
BP	Low DO	RPL40B	2	0.637	1.295E-01	ribosomal large subunit export from nucleus; cytoplasmic translation

BP	Low DO	RPL1B	2	0.610	1.553E-01	ribosomal large subunit export from nucleus; cytoplasmic translation
BP	Low DO	MUP1	1	2.911	1.215E-20	amino acid transmembrane transport
BP	Low DO	OPT1	1	2.792	1.281E-13	oligopeptide transmembrane transport
BP	Low DO	MET14	1	2.727	3.601E-17	hydrogen sulfide metabolic process
BP	Low DO	YCT1	1	2.700	4.942E-10	amino acid transmembrane transport
BP	Low DO	SEO1	1	2.487	2.040E-13	oligopeptide transmembrane transport
BP	Low DO	MMP1	1	2.406	1.403E-14	amino acid transmembrane transport
BP	Low DO	NOP7	1	2.399	5.227E-08	maturation of LSU-rRNA from tricistronic rRNA transcript (SSU-rRNA, 5.8S rRNA, LSU-rRNA)
BP	Low DO	MET3	1	2.382	7.626E-13	hydrogen sulfide metabolic process
BP	Low DO	MET16	1	2.299	6.147E-15	hydrogen sulfide metabolic process
BP	Low DO	ECM1	1	2.230	1.057E-07	ribosomal large subunit export from nucleus
BP	Low DO	MET10	1	2.154	7.258E-11	hydrogen sulfide metabolic process
BP	Low DO	SDA1	1	2.108	9.484E-09	ribosomal large subunit export from nucleus
BP	Low DO	MET5	1	2.104	5.915E-12	hydrogen sulfide metabolic process
BP	Low DO	KRR1	1	2.077	6.654E-08	endonucleolytic cleavage in ITS1 to separate SSU-rRNA from 5.8S rRNA and LSU-rRNA from tricistronic rRNA transcript (SSU-rRNA, 5.8S rRNA, LSU-rRNA)
BP	Low DO	DIM1	1	2.050	9.776E-07	rRNA methylation
BP	Low DO	YTM1	1	2.017	5.938E-08	maturation of LSU-rRNA from tricistronic rRNA transcript (SSU-rRNA, 5.8S rRNA, LSU-rRNA)
BP	Low DO	BMT5	1	1.977	5.882E-06	rRNA methylation
BP	Low DO	YVH1	1	1.957	1.159E-07	ribosomal large subunit export from nucleus
BP	Low DO	URB1	1	1.947	1.149E-08	maturation of LSU-rRNA from tricistronic rRNA transcript (SSU-rRNA, 5.8S rRNA, LSU-rRNA)
BP	Low DO	LRP1	1	1.908	2.256E-07	nuclear polyadenylation-dependent rRNA catabolic process
BP	Low DO	NIP7	1	1.842	1.018E-05	maturation of LSU-rRNA from tricistronic rRNA transcript (SSU-rRNA, 5.8S rRNA, LSU-rRNA)
BP	Low DO	NOP4	1	1.812	1.125E-06	maturation of LSU-rRNA from tricistronic rRNA transcript (SSU-rRNA, 5.8S rRNA, LSU-rRNA)
BP	Low DO	DBP7	1	1.801	9.698E-06	maturation of LSU-rRNA from tricistronic rRNA transcript (SSU-rRNA, 5.8S rRNA, LSU-rRNA)
BP	Low DO	RIX7	1	1.789	7.866E-07	ribosomal large subunit export from nucleus
BP	Low DO	CYS4	1	1.787	1.180E-06	hydrogen sulfide metabolic process
BP	Low DO	DBP10	1	1.776	1.398E-08	maturation of LSU-rRNA from tricistronic rRNA transcript (SSU-rRNA, 5.8S rRNA, LSU-rRNA)
BP	Low DO	ARX1	1	1.758	5.139E-06	ribosomal large subunit export from nucleus
BP	Low DO	BRX1	1	1.757	1.745E-05	maturation of LSU-rRNA from tricistronic rRNA transcript (SSU-rRNA, 5.8S rRNA, LSU-rRNA)
BP	Low DO	NMD3	1	1.754	1.098E-05	ribosomal large subunit export from nucleus
BP	Low DO	MAK5	1	1.739	3.589E-06	maturation of LSU-rRNA from tricistronic rRNA transcript (SSU-rRNA, 5.8S rRNA, LSU-rRNA)
BP	Low DO	DBP3	1	1.739	3.988E-06	maturation of LSU-rRNA from tricistronic rRNA transcript (SSU-rRNA, 5.8S rRNA, LSU-rRNA)
BP	Low DO	SAM3	1	1.733	5.515E-06	amino acid transmembrane transport
BP	Low DO	MRT4	1	1.726	3.613E-05	ribosomal large subunit export from nucleus
BP	Low DO	AQR1	1	1.690	5.866E-04	amino acid transmembrane transport
BP	Low DO	ENP1	1	1.683	4.569E-07	endonucleolytic cleavage in ITS1 to separate SSU-rRNA from 5.8S rRNA and LSU-rRNA from tricistronic rRNA transcript (SSU-rRNA, 5.8S rRNA, LSU-rRNA)
BP	Low DO	NOP56	1	1.674	3.932E-06	rRNA methylation
BP	Low DO	BCP1	1	1.671	1.664E-05	ribosomal large subunit export from nucleus
BP	Low DO	TSR2	1	1.666	8.579E-06	maturation of LSU-rRNA from tricistronic rRNA transcript (SSU-rRNA, 5.8S rRNA, LSU-rRNA)
BP	Low DO	MAK16	1	1.639	1.753E-06	maturation of LSU-rRNA from tricistronic rRNA transcript (SSU-rRNA, 5.8S rRNA, LSU-rRNA)
BP	Low DO	CIC1	1	1.635	1.063E-04	maturation of LSU-rRNA from tricistronic rRNA transcript (SSU-rRNA, 5.8S rRNA, LSU-rRNA)
BP	Low DO	DBP9	1	1.588	1.460E-05	maturation of LSU-rRNA from tricistronic rRNA transcript (SSU-rRNA, 5.8S rRNA, LSU-rRNA)

BP	Low DO	SSF1	1	1.571	1.778E-05	maturation of LSU-rRNA from tricistronic rRNA transcript (SSU-rRNA, 5.8S rRNA, LSU-rRNA)
BP	Low DO	ELP2	1	1.569	9.390E-06	tRNA wobble uridine modification
BP	Low DO	PRP43	1	1.548	1.027E-05	maturation of LSU-rRNA from tricistronic rRNA transcript (SSU-rRNA, 5.8S rRNA, LSU-rRNA)
BP	Low DO	IPI1	1	1.544	6.154E-05	maturation of LSU-rRNA from tricistronic rRNA transcript (SSU-rRNA, 5.8S rRNA, LSU-rRNA)
BP	Low DO	YEF3	1	1.515	7.639E-05	cytoplasmic translation
BP	Low DO	RRP15	1	1.515	8.773E-05	maturation of LSU-rRNA from tricistronic rRNA transcript (SSU-rRNA, 5.8S rRNA, LSU-rRNA)
BP	Low DO	MUP3	1	1.513	1.054E-05	amino acid transmembrane transport
BP	Low DO	NOP12	1	1.511	1.017E-04	maturation of LSU-rRNA from tricistronic rRNA transcript (SSU-rRNA, 5.8S rRNA, LSU-rRNA)
BP	Low DO	KRI1	1	1.508	6.949E-06	endonucleolytic cleavage in ITS1 to separate SSU-rRNA from 5.8S rRNA and LSU-rRNA from tricistronic rRNA transcript (SSU-rRNA, 5.8S rRNA, LSU-rRNA)
BP	Low DO	NSA2	1	1.499	1.506E-04	maturation of LSU-rRNA from tricistronic rRNA transcript (SSU-rRNA, 5.8S rRNA, LSU-rRNA)
BP	Low DO	MAK11	1	1.488	6.458E-05	maturation of LSU-rRNA from tricistronic rRNA transcript (SSU-rRNA, 5.8S rRNA, LSU-rRNA)
BP	Low DO	IKI3	1	1.468	7.765E-06	tRNA wobble uridine modification
BP	Low DO	RLP7	1	1.430	2.309E-04	maturation of LSU-rRNA from tricistronic rRNA transcript (SSU-rRNA, 5.8S rRNA, LSU-rRNA)
BP	Low DO	NIP1	1	1.409	2.683E-05	cytoplasmic translation
BP	Low DO	RPF2	1	1.388	1.466E-05	maturation of LSU-rRNA from tricistronic rRNA transcript (SSU-rRNA, 5.8S rRNA, LSU-rRNA)
BP	Low DO	NOP8	1	1.382	1.702E-05	maturation of LSU-rRNA from tricistronic rRNA transcript (SSU-rRNA, 5.8S rRNA, LSU-rRNA)
BP	Low DO	HAS1	1	1.373	1.114E-05	maturation of LSU-rRNA from tricistronic rRNA transcript (SSU-rRNA, 5.8S rRNA, LSU-rRNA)
BP	Low DO	BCD1	1	1.364	2.082E-04	maturation of LSU-rRNA from tricistronic rRNA transcript (SSU-rRNA, 5.8S rRNA, LSU-rRNA)
BP	Low DO	ERB1	1	1.326	4.612E-05	maturation of LSU-rRNA from tricistronic rRNA transcript (SSU-rRNA, 5.8S rRNA, LSU-rRNA)
BP	Low DO	ANB1	1	1.326	7.840E-02	cytoplasmic translation
BP	Low DO	BMT2	1	1.300	4.835E-04	rRNA methylation
BP	Low DO	GAP1	1	1.300	2.390E-05	amino acid transmembrane transport
BP	Low DO	RRP8	1	1.298	2.269E-04	rRNA methylation
BP	Low DO	TIF3	1	1.297	3.091E-04	cytoplasmic translation
BP	Low DO	TRM7	1	1.296	2.082E-04	cytoplasmic translation
BP	Low DO	RPL9A	1	1.271	2.101E-03	cytoplasmic translation
BP	Low DO	YMC2	1	1.271	2.219E-04	amino acid transmembrane transport
BP	Low DO	NOP15	1	1.236	1.397E-03	maturation of LSU-rRNA from tricistronic rRNA transcript (SSU-rRNA, 5.8S rRNA, LSU-rRNA)
BP	Low DO	TPK3	1	1.235	3.473E-04	cytoplasmic translation
BP	Low DO	HIT1	1	1.232	1.180E-03	maturation of LSU-rRNA from tricistronic rRNA transcript (SSU-rRNA, 5.8S rRNA, LSU-rRNA)
BP	Low DO	SSB1	1	1.207	1.038E-03	cytoplasmic translation
BP	Low DO	NOP1	1	1.204	3.086E-03	rRNA methylation
BP	Low DO	URM1	1	1.203	7.337E-04	tRNA wobble uridine modification
BP	Low DO	SAP185	1	1.181	2.269E-04	tRNA wobble uridine modification
BP	Low DO	NOG2	1	1.168	2.434E-03	ribosomal large subunit export from nucleus
BP	Low DO	RBG1	1	1.167	2.793E-04	cytoplasmic translation
BP	Low DO	ELP3	1	1.155	1.241E-03	tRNA wobble uridine modification
BP	Low DO	GCD6	1	1.127	1.057E-03	cytoplasmic translation
BP	Low DO	VSB1	1	1.112	1.226E-03	amino acid transmembrane transport
BP	Low DO	ODC2	1	1.101	3.162E-03	amino acid transmembrane transport
BP	Low DO	TMA19	1	1.096	1.079E-02	cytoplasmic translation
BP	Low DO	BUD20	1	1.084	8.811E-03	ribosomal large subunit export from nucleus
BP	Low DO	SSF2	1	1.074	1.387E-03	maturation of LSU-rRNA from tricistronic rRNA transcript (SSU-rRNA, 5.8S rRNA, LSU-rRNA)

BP	Low DO	RPS4B	1	1.064	8.994E-03	cytoplasmic translation
BP	Low DO	TAT1	1	1.052	1.115E-02	amino acid transmembrane transport
BP	Low DO	RCM1	1	1.040	1.562E-03	rRNA methylation
BP	Low DO	TRM9	1	1.039	1.950E-03	tRNA wobble uridine modification
BP	Low DO	RBG2	1	1.024	3.387E-03	cytoplasmic translation
BP	Low DO	AGP3	1	1.017	4.636E-03	amino acid transmembrane transport
BP	Low DO	SSZ1	1	1.015	7.890E-03	cytoplasmic translation
BP	Low DO	NCS2	1	1.007	3.603E-03	tRNA wobble uridine modification
BP	Low DO	MPP6	1	0.998	1.054E-02	nuclear polyadenylation-dependent rRNA catabolic process
BP	Low DO	DED1	1	0.987	2.331E-02	cytoplasmic translation
BP	Low DO	TAT2	1	0.978	1.231E-02	amino acid transmembrane transport
BP	Low DO	MTO1	1	0.977	3.174E-03	tRNA wobble uridine modification
BP	Low DO	TIF35	1	0.959	7.859E-03	cytoplasmic translation
BP	Low DO	LAS1	1	0.957	7.282E-03	maturation of LSU-rRNA from tricistronic rRNA transcript (SSU-rRNA, 5.8S rRNA, LSU-rRNA)
BP	Low DO	GIR2	1	0.956	3.538E-03	cytoplasmic translation
BP	Low DO	KTI12	1	0.954	1.493E-02	tRNA wobble uridine modification
BP	Low DO	AVT4	1	0.953	7.395E-03	amino acid transmembrane transport
BP	Low DO	GIS2	1	0.946	1.210E-02	cytoplasmic translation
BP	Low DO	SUP45	1	0.936	4.239E-03	cytoplasmic translation
BP	Low DO	GCN3	1	0.931	1.334E-02	cytoplasmic translation
BP	Low DO	VBA2	1	0.916	1.268E-02	amino acid transmembrane transport
BP	Low DO	JJJ1	1	0.910	6.475E-03	ribosomal large subunit export from nucleus
BP	Low DO	TIF4631	1	0.909	5.134E-03	cytoplasmic translation
BP	Low DO	AGP1	1	0.905	2.731E-02	amino acid transmembrane transport
BP	Low DO	CAN1	1	0.900	1.450E-02	amino acid transmembrane transport
BP	Low DO	SNU13	1	0.898	1.066E-02	rRNA methylation
BP	Low DO	CFD1	1	0.893	1.684E-02	tRNA wobble uridine modification
BP	Low DO	VBA4	1	0.875	1.328E-02	amino acid transmembrane transport
BP	Low DO	RRP17	1	0.853	2.379E-02	maturation of LSU-rRNA from tricistronic rRNA transcript (SSU-rRNA, 5.8S rRNA, LSU-rRNA)
BP	Low DO	IKI1	1	0.849	2.460E-02	tRNA wobble uridine modification
BP	Low DO	TIF5	1	0.846	2.269E-02	cytoplasmic translation
BP	Low DO	ORT1	1	0.839	4.492E-02	amino acid transmembrane transport
BP	Low DO	MRH4	1	0.838	2.518E-02	maturation of LSU-rRNA from tricistronic rRNA transcript (SSU-rRNA, 5.8S rRNA, LSU-rRNA)
BP	Low DO	RPS12	1	0.827	4.336E-02	cytoplasmic translation
BP	Low DO	RPS22A	1	0.799	5.386E-02	cytoplasmic translation
BP	Low DO	RPP2B	1	0.783	5.205E-02	cytoplasmic translation
BP	Low DO	RPL13A	1	0.783	4.032E-02	cytoplasmic translation
BP	Low DO	RPS2	1	0.782	5.121E-02	cytoplasmic translation
BP	Low DO	RPS8B	1	0.772	7.597E-02	cytoplasmic translation
BP	Low DO	NUP85	1	0.770	3.587E-02	ribosomal large subunit export from nucleus
BP	Low DO	RPS1A	1	0.769	4.113E-02	cytoplasmic translation
BP	Low DO	RPL35B	1	0.760	6.513E-02	cytoplasmic translation
BP	Low DO	RTC2	1	0.759	1.114E-01	amino acid transmembrane transport
BP	Low DO	RPS3	1	0.758	4.852E-02	cytoplasmic translation
BP	Low DO	RPL23A	1	0.753	5.407E-02	cytoplasmic translation
BP	Low DO	RPL10	1	0.737	6.695E-02	cytoplasmic translation
BP	Low DO	TAE1	1	0.728	3.976E-02	cytoplasmic translation
BP	Low DO	RPS26B	1	0.719	4.492E-02	cytoplasmic translation
BP	Low DO	LYP1	1	0.710	4.776E-02	amino acid transmembrane transport
BP	Low DO	RPS18B	1	0.699	6.762E-02	cytoplasmic translation
BP	Low DO	RPS31	1	0.696	1.032E-01	cytoplasmic translation
BP	Low DO	RPL15A	1	0.690	8.222E-02	cytoplasmic translation
BP	Low DO	FUN12	1	0.690	5.433E-02	cytoplasmic translation
BP	Low DO	RPL20B	1	0.685	9.185E-02	cytoplasmic translation
BP	Low DO	NUP82	1	0.684	6.486E-02	ribosomal large subunit export from nucleus
BP	Low DO	RPS0A	1	0.682	5.821E-02	cytoplasmic translation
BP	Low DO	RPL31B	1	0.681	7.565E-02	cytoplasmic translation
BP	Low DO	YMC1	1	0.676	6.721E-02	amino acid transmembrane transport

BP	Low DO	RPL27B	1	0.674	8.279E-02	cytoplasmic translation
BP	Low DO	RPL21A	1	0.671	6.758E-02	cytoplasmic translation
BP	Low DO	RPL24B	1	0.669	9.638E-02	cytoplasmic translation
BP	Low DO	RPL6A	1	0.662	1.270E-01	cytoplasmic translation
BP	Low DO	NCS6	1	0.662	7.143E-02	tRNA wobble uridine modification
BP	Low DO	RPB10	1	0.661	1.367E-01	termination of RNA polymerase III transcription
BP	Low DO	TMA46	1	0.650	1.052E-01	cytoplasmic translation
BP	Low DO	RPS25B	1	0.643	1.454E-01	cytoplasmic translation
BP	Low DO	ELP4	1	0.642	9.682E-02	tRNA wobble uridine modification
BP	Low DO	QDR2	1	0.625	1.302E-01	amino acid transmembrane transport
BP	Low DO	RPB8	1	0.624	1.056E-01	termination of RNA polymerase III transcription
BP	Low DO	ASC1	1	0.623	1.077E-01	cytoplasmic translation
BP	Low DO	RPP1A	1	0.622	1.644E-01	cytoplasmic translation
BP	Low DO	RPL28	1	0.622	1.617E-01	cytoplasmic translation
BP	Low DO	RPS24B	1	0.620	1.545E-01	cytoplasmic translation
BP	Low DO	SUI3	1	0.615	9.310E-02	cytoplasmic translation
BP	Low DO	GCN1	1	0.614	9.382E-02	cytoplasmic translation
BP	Low DO	CDC33	1	0.614	1.286E-01	cytoplasmic translation
BP	Low DO	RPL16A	1	0.611	1.286E-01	cytoplasmic translation
BP	Low DO	RPL5	1	0.610	9.786E-02	cytoplasmic translation
BP	Low DO	HIP1	1	0.604	1.083E-01	amino acid transmembrane transport
BP	Low DO	RPL8B	1	0.592	1.213E-01	cytoplasmic translation
BP	Low DO	RPL4A	1	0.589	1.783E-01	cytoplasmic translation
BP	Low DO	RPL11A	1	0.585	1.485E-01	cytoplasmic translation
BP	Low DO	RPL2B	1	0.585	1.638E-01	cytoplasmic translation
BP	Low DO	RPL21B	1	0.581	1.637E-01	cytoplasmic translation
BP	Low DO	TIF2	1	0.576	1.248E-01	cytoplasmic translation
BP	Low DO	UBA4	1	0.574	1.426E-01	tRNA wobble uridine modification
BP	Low DO	RPS17B	1	0.564	2.009E-01	cytoplasmic translation
BP	Low DO	RPL26A	1	0.556	2.130E-01	cytoplasmic translation
BP	Low DO	RPL39	1	0.554	2.169E-01	cytoplasmic translation
BP	Low DO	TIF11	1	0.554	1.434E-01	cytoplasmic translation
BP	Low DO	NIC96	1	0.550	1.422E-01	ribosomal large subunit export from nucleus
BP	Low DO	RQC2	1	0.547	3.004E-01	cytoplasmic translation
BP	Low DO	ISU2	1	0.537	1.666E-01	tRNA wobble uridine modification
BP	Low DO	RPS7A	1	0.536	2.109E-01	cytoplasmic translation
BP	Low DO	RPL17B	1	0.535	1.681E-01	cytoplasmic translation
BP	Low DO	RPL42A	1	0.524	2.560E-01	cytoplasmic translation
BP	Low DO	RPL24A	1	0.523	2.189E-01	cytoplasmic translation
BP	Low DO	RPS10A	1	0.523	2.244E-01	cytoplasmic translation
BP	Low DO	RPL26B	1	0.521	2.414E-01	cytoplasmic translation
BP	Low DO	CIA1	1	0.518	1.758E-01	tRNA wobble uridine modification
BP	Low DO	SES1	1	0.507	1.848E-01	cytoplasmic translation
BP	Low DO	RPS20	1	0.502	2.554E-01	cytoplasmic translation
BP	Low DO	RPS21B	1	0.499	2.568E-01	cytoplasmic translation
BP	Low DO	RPL12A	1	0.497	2.145E-01	cytoplasmic translation
BP	Low DO	RPL14A	1	0.496	2.363E-01	cytoplasmic translation
BP	Low DO	RPS13	1	0.494	2.544E-01	cytoplasmic translation
BP	Low DO	RPL27A	1	0.491	2.582E-01	cytoplasmic translation
BP	Low DO	RPL33B	1	0.488	2.717E-01	cytoplasmic translation
BP	Low DO	RPL11B	1	0.485	2.139E-01	cytoplasmic translation
BP	Low DO	RPL18B	1	0.482	2.571E-01	cytoplasmic translation
BP	Low DO	RPL19B	1	0.476	2.184E-01	cytoplasmic translation
BP	Low DO	RPS4A	1	0.475	2.347E-01	cytoplasmic translation
BP	Low DO	RPS1B	1	0.475	2.454E-01	cytoplasmic translation
BP	Low DO	GCD11	1	0.465	2.855E-01	cytoplasmic translation
BP	Low DO	RPS21A	1	0.461	3.084E-01	cytoplasmic translation
BP	Low DO	RPL23B	1	0.461	3.011E-01	cytoplasmic translation
BP	Low DO	RPS18A	1	0.457	2.928E-01	cytoplasmic translation
BP	Low DO	GCD1	1	0.457	2.668E-01	cytoplasmic translation

BP	Low DO	RPL37B	1	0.440	3.006E-01	cytoplasmic translation
BP	Low DO	PRT1	1	0.434	2.664E-01	cytoplasmic translation
BP	Low DO	RPL12B	1	0.433	3.125E-01	cytoplasmic translation
BP	Low DO	TMA22	1	0.420	2.806E-01	cytoplasmic translation
BP	Low DO	RPL20A	1	0.402	3.171E-01	cytoplasmic translation
MF	High DO	HTB1	2	-3.153	6.579E-22	protein heterodimerization activity; structural constituent of chromatin
MF	High DO	GPX1	2	-2.605	3.601E-17	glutathione peroxidase activity; thioredoxin-dependent peroxiredoxin activity
MF	High DO	TFS1	2	-2.555	1.503E-09	lipid binding; endopeptidase inhibitor activity
MF	High DO	HHF2	2	-1.580	1.486E-06	protein heterodimerization activity; structural constituent of chromatin
MF	High DO	HHT2	2	-1.540	3.810E-07	protein heterodimerization activity; structural constituent of chromatin
MF	High DO	HHF1	2	-1.507	6.995E-06	protein heterodimerization activity; structural constituent of chromatin
MF	High DO	HTB2	2	-1.419	7.471E-06	protein heterodimerization activity; structural constituent of chromatin
MF	High DO	HTA2	2	-1.391	6.813E-05	protein heterodimerization activity; structural constituent of chromatin
MF	High DO	HHT1	2	-1.240	5.992E-04	protein heterodimerization activity; structural constituent of chromatin
MF	High DO	HYR1	2	-0.989	3.142E-03	glutathione peroxidase activity; thioredoxin-dependent peroxiredoxin activity
MF	High DO	HTA1	2	-0.941	7.378E-03	protein heterodimerization activity; structural constituent of chromatin
MF	High DO	PAU19	1	-3.178	1.847E-08	structural constituent of cell wall
MF	High DO	CWP1	1	-3.040	1.359E-12	structural constituent of cell wall
MF	High DO	PAI3	1	-2.718	7.162E-13	endopeptidase inhibitor activity
MF	High DO	DCS2	1	-2.651	4.926E-14	protein heterodimerization activity
MF	High DO	SPI1	1	-2.449	8.408E-14	structural constituent of cell wall
MF	High DO	AHP1	1	-2.307	9.691E-06	thioredoxin-dependent peroxiredoxin activity
MF	High DO	PAU15	1	-2.267	3.633E-02	structural constituent of cell wall
MF	High DO	POX1	1	-2.246	1.251E-01	lipid binding
MF	High DO	HSP12	1	-2.119	3.253E-10	lipid binding
MF	High DO	PAU5	1	-2.023	8.596E-03	structural constituent of cell wall
MF	High DO	PAU6	1	-2.022	1.524E-03	structural constituent of cell wall
MF	High DO	CCW12	1	-1.802	1.036E-05	structural constituent of cell wall
MF	High DO	YKL091C	1	-1.789	4.537E-06	lipid binding
MF	High DO	TSA2	1	-1.770	1.322E-08	thioredoxin-dependent peroxiredoxin activity
MF	High DO	NPC2	1	-1.677	5.356E-05	lipid binding
MF	High DO	CWP2	1	-1.652	7.747E-04	structural constituent of cell wall
MF	High DO	HHO1	1	-1.646	8.834E-07	structural constituent of chromatin
MF	High DO	PAU17	1	-1.606	5.529E-05	structural constituent of cell wall
MF	High DO	SIP18	1	-1.557	2.235E-05	lipid binding
MF	High DO	GRX2	1	-1.552	1.425E-06	glutathione peroxidase activity
MF	High DO	GGA1	1	-1.509	1.420E-05	lipid binding
MF	High DO	SCS2	1	-1.478	2.423E-03	lipid binding
MF	High DO	PRX1	1	-1.425	4.581E-06	thioredoxin-dependent peroxiredoxin activity
MF	High DO	PAU2	1	-1.389	2.968E-03	structural constituent of cell wall
MF	High DO	DLS1	1	-1.358	1.007E-03	protein heterodimerization activity
MF	High DO	YSC84	1	-1.348	9.874E-05	lipid binding
MF	High DO	ATG8	1	-1.343	4.880E-05	lipid binding
MF	High DO	PMP3	1	-1.319	3.701E-04	lipid binding
MF	High DO	LSP1	1	-1.283	6.310E-03	lipid binding
MF	High DO	IST2	1	-1.260	1.206E-02	lipid binding
MF	High DO	CCW14	1	-1.254	7.307E-03	structural constituent of cell wall
MF	High DO	LAM5	1	-1.246	3.885E-03	lipid binding
MF	High DO	DAN4	1	-1.231	3.473E-02	structural constituent of cell wall
MF	High DO	GRX1	1	-1.218	8.773E-05	glutathione peroxidase activity
MF	High DO	PIL1	1	-1.217	1.014E-02	lipid binding

MF	High DO	DCS1	1	-1.204	1.876E-04	protein heterodimerization activity
MF	High DO	GTT1	1	-1.185	4.721E-04	glutathione peroxidase activity
MF	High DO	PAU23	1	-1.181	1.441E-02	structural constituent of cell wall
MF	High DO	OSH2	1	-1.125	4.321E-03	lipid binding
MF	High DO	OSH6	1	-1.093	2.311E-03	lipid binding
MF	High DO	TSA1	1	-1.074	1.373E-03	thioredoxin-dependent peroxiredoxin activity
MF	High DO	YHR138C	1	-1.070	1.401E-03	endopeptidase inhibitor activity
MF	High DO	PBI2	1	-1.039	1.064E-03	endopeptidase inhibitor activity
MF	High DO	CAM1	1	-1.018	1.388E-02	lipid binding
MF	High DO	ENT2	1	-0.984	1.987E-02	lipid binding
MF	High DO	LAM4	1	-0.962	1.367E-02	lipid binding
MF	High DO	ATG18	1	-0.931	4.113E-02	lipid binding
MF	High DO	SWH1	1	-0.922	9.638E-02	lipid binding
MF	High DO	TCB3	1	-0.904	7.446E-02	lipid binding
MF	High DO	DOT5	1	-0.891	4.136E-02	thioredoxin-dependent peroxiredoxin activity
MF	High DO	MHF1	1	-0.835	1.454E-02	protein heterodimerization activity
MF	High DO	COQ9	1	-0.829	1.472E-02	lipid binding
MF	High DO	SNX4	1	-0.815	1.531E-02	lipid binding
MF	High DO	SNX41	1	-0.769	1.050E-01	lipid binding
MF	High DO	RVS167	1	-0.767	5.726E-02	lipid binding
MF	High DO	ATG44	1	-0.764	2.829E-02	lipid binding
MF	High DO	NVJ2	1	-0.756	8.263E-02	lipid binding
MF	High DO	HTZ1	1	-0.744	4.336E-02	structural constituent of chromatin
MF	High DO	PIB1	1	-0.741	4.073E-02	lipid binding
MF	High DO	TCB1	1	-0.724	8.215E-02	lipid binding
MF	High DO	SLM1	1	-0.648	7.541E-02	lipid binding
MF	High DO	ENT3	1	-0.602	1.615E-01	lipid binding
MF	High DO	VPS13	1	-0.579	1.956E-01	lipid binding
MF	High DO	YPT35	1	-0.569	2.490E-01	lipid binding
MF	High DO	ARC1	1	-0.564	2.963E-01	lipid binding
MF	High DO	SPO20	1	-0.547	2.348E-01	lipid binding
MF	High DO	PDR17	1	-0.533	2.305E-01	lipid binding
MF	High DO	NCR1	1	-0.526	2.189E-01	lipid binding
MF	High DO	ATG2	1	-0.521	2.774E-01	lipid binding
MF	High DO	ACB1	1	-0.511	1.685E-01	lipid binding
MF	High DO	MSS4	1	-0.510	2.678E-01	lipid binding
MF	High DO	SLA1	1	-0.507	1.909E-01	lipid binding
MF	High DO	CDC11	1	-0.499	2.338E-01	lipid binding
MF	High DO	HSV2	1	-0.476	2.230E-01	lipid binding
MF	High DO	MGM1	1	-0.459	2.886E-01	lipid binding
MF	High DO	ATG20	1	-0.450	2.719E-01	lipid binding
MF	Low DO	MUP1	3	2.911	1.215E-20	sulfur compound transmembrane transporter activity; organic cation transmembrane transporter activity; neutral L-amino acid transmembrane transporter activity
MF	Low DO	YCT1	3	2.700	4.942E-10	sulfur compound transmembrane transporter activity; organic cation transmembrane transporter activity; neutral L-amino acid transmembrane transporter activity
MF	Low DO	FAL1	3	2.573	7.754E-12	mRNA binding; RNA helicase activity; ATP hydrolysis activity
MF	Low DO	NOP7	3	2.399	5.227E-08	mRNA binding; large ribosomal subunit rRNA binding; ribonucleoprotein complex binding
MF	Low DO	RRP5	3	2.099	1.839E-09	mRNA binding; U3 snoRNA binding; rRNA primary transcript binding
MF	Low DO	BMS1	3	1.999	3.991E-09	mRNA binding; U3 snoRNA binding; ATP hydrolysis activity
MF	Low DO	TRM11	3	1.945	2.491E-05	tRNA binding; tRNA methyltransferase activity; N-methyltransferase activity
MF	Low DO	MTR4	3	1.895	3.712E-08	mRNA binding; RNA helicase activity; ATP hydrolysis activity
MF	Low DO	DBP3	3	1.739	3.988E-06	mRNA binding; RNA helicase activity; ATP hydrolysis activity
MF	Low DO	DBP2	3	1.680	1.832E-03	mRNA binding; RNA helicase activity; ATP hydrolysis activity
MF	Low DO	TRM1	3	1.640	1.027E-05	tRNA binding; tRNA methyltransferase activity; N-methyltransferase activity
MF	Low DO	DBP9	3	1.588	1.460E-05	mRNA binding; RNA helicase activity; ATP hydrolysis activity

MF	Low DO	PRP43	3	1.548	1.027E-05	mRNA binding; RNA helicase activity; ATP hydrolysis activity
MF	Low DO	DED1	3	0.987	2.331E-02	mRNA binding; RNA helicase activity; ATP hydrolysis activity
MF	Low DO	YLR419W	3	0.915	5.403E-03	mRNA binding; RNA helicase activity; ATP hydrolysis activity
MF	Low DO	NEW1	3	0.863	1.312E-02	mRNA binding; ATP hydrolysis activity; ribonucleoprotein complex binding
MF	Low DO	MRH4	3	0.838	2.518E-02	RNA helicase activity; ATP hydrolysis activity; large ribosomal subunit rRNA binding
MF	Low DO	DHR2	2	2.587	4.780E-09	RNA helicase activity; ATP hydrolysis activity
MF	Low DO	DRS1	2	2.107	2.340E-07	RNA helicase activity; ATP hydrolysis activity
MF	Low DO	DIM1	2	2.050	9.776E-07	rRNA methyltransferase activity; N-methyltransferase activity
MF	Low DO	MRD1	2	2.024	7.505E-07	mRNA binding; rRNA primary transcript binding
MF	Low DO	BMT5	2	1.977	5.882E-06	rRNA methyltransferase activity; N-methyltransferase activity
MF	Low DO	HCA4	2	1.879	8.361E-08	RNA helicase activity; ATP hydrolysis activity
MF	Low DO	NOP4	2	1.812	1.125E-06	mRNA binding; large ribosomal subunit rRNA binding
MF	Low DO	DBP7	2	1.801	9.698E-06	RNA helicase activity; ATP hydrolysis activity
MF	Low DO	RIX7	2	1.789	7.866E-07	ATP hydrolysis activity; ribonucleoprotein complex binding
MF	Low DO	ECM16	2	1.783	6.654E-08	RNA helicase activity; ATP hydrolysis activity
MF	Low DO	DBP10	2	1.776	1.398E-08	RNA helicase activity; ATP hydrolysis activity
MF	Low DO	NMD3	2	1.754	1.098E-05	large ribosomal subunit rRNA binding; ribonucleoprotein complex binding
MF	Low DO	MAK5	2	1.739	3.589E-06	RNA helicase activity; ATP hydrolysis activity
MF	Low DO	SAM3	2	1.733	5.515E-06	sulfur compound transmembrane transporter activity; organic cation transmembrane transporter activity
MF	Low DO	AQR1	2	1.690	5.866E-04	organic cation transmembrane transporter activity; neutral L-amino acid transmembrane transporter activity
MF	Low DO	ROK1	2	1.654	9.307E-07	RNA helicase activity; ATP hydrolysis activity
MF	Low DO	UTP8	2	1.652	2.377E-05	U3 snoRNA binding; tRNA binding
MF	Low DO	CIC1	2	1.635	1.063E-04	large ribosomal subunit rRNA binding; ribonucleoprotein complex binding
MF	Low DO	YEF3	2	1.515	7.639E-05	ATP hydrolysis activity; ribonucleoprotein complex binding
MF	Low DO	MUP3	2	1.513	1.054E-05	sulfur compound transmembrane transporter activity; organic cation transmembrane transporter activity
MF	Low DO	UTP9	2	1.494	7.310E-05	U3 snoRNA binding; tRNA binding
MF	Low DO	PUF6	2	1.474	4.450E-06	mRNA binding; large ribosomal subunit rRNA binding
MF	Low DO	NAN1	2	1.462	2.113E-04	mRNA binding; U3 snoRNA binding
MF	Low DO	RLP7	2	1.430	2.309E-04	mRNA binding; rRNA primary transcript binding
MF	Low DO	HAS1	2	1.373	1.114E-05	RNA helicase activity; ATP hydrolysis activity
MF	Low DO	RRP3	2	1.356	3.899E-04	RNA helicase activity; ATP hydrolysis activity
MF	Low DO	ERB1	2	1.326	4.612E-05	large ribosomal subunit rRNA binding; ribonucleoprotein complex binding
MF	Low DO	GAP1	2	1.300	2.390E-05	organic cation transmembrane transporter activity; neutral L-amino acid transmembrane transporter activity
MF	Low DO	RLI1	2	1.299	4.560E-04	ATP hydrolysis activity; ribonucleoprotein complex binding
MF	Low DO	TIF3	2	1.297	3.091E-04	mRNA binding; ribonucleoprotein complex binding
MF	Low DO	YMC2	2	1.271	2.219E-04	organic cation transmembrane transporter activity; neutral L-amino acid transmembrane transporter activity
MF	Low DO	LHP1	2	1.259	1.922E-03	mRNA binding; tRNA binding
MF	Low DO	NOP1	2	1.204	3.086E-03	rRNA methyltransferase activity; N-methyltransferase activity
MF	Low DO	DBP8	2	1.197	4.751E-04	RNA helicase activity; ATP hydrolysis activity
MF	Low DO	LOC1	2	1.160	3.297E-04	mRNA binding; ribonucleoprotein complex binding
MF	Low DO	ALA1	2	1.151	2.657E-04	mRNA binding; tRNA binding
MF	Low DO	ILS1	2	1.113	4.125E-04	mRNA binding; tRNA binding
MF	Low DO	TRM9	2	1.039	1.950E-03	tRNA binding; tRNA methyltransferase activity
MF	Low DO	THI7	2	1.036	1.591E-02	sulfur compound transmembrane transporter activity; organic cation transmembrane transporter activity
MF	Low DO	NCL1	2	1.034	2.067E-03	tRNA binding; tRNA methyltransferase activity
MF	Low DO	SDO1	2	0.977	1.634E-02	large ribosomal subunit rRNA binding; rRNA primary transcript binding
MF	Low DO	AVT4	2	0.953	7.395E-03	organic cation transmembrane transporter activity; neutral L-amino acid transmembrane transporter activity
MF	Low DO	TRM8	2	0.947	4.928E-03	tRNA binding; tRNA methyltransferase activity

MF	Low DO	TRM10	2	0.944	4.797E-03	tRNA binding; tRNA methyltransferase activity
MF	Low DO	GCN20	2	0.929	9.949E-03	ATP hydrolysis activity; ribonucleoprotein complex binding
MF	Low DO	AGP1	2	0.905	2.731E-02	organic cation transmembrane transporter activity; neutral L-amino acid transmembrane transporter activity
MF	Low DO	IKI1	2	0.849	2.460E-02	tRNA binding; ATP hydrolysis activity
MF	Low DO	SPB4	2	0.827	1.362E-02	RNA helicase activity; ATP hydrolysis activity
MF	Low DO	BRR2	2	0.713	4.073E-02	RNA helicase activity; ATP hydrolysis activity
MF	Low DO	SUP35	2	0.681	6.018E-02	mRNA binding; ribonucleoprotein complex binding
MF	Low DO	YMC1	2	0.676	6.721E-02	organic cation transmembrane transporter activity; neutral L-amino acid transmembrane transporter activity
MF	Low DO	MSS116	2	0.664	8.681E-02	mRNA binding; ATP hydrolysis activity
MF	Low DO	ELP4	2	0.642	9.682E-02	tRNA binding; ATP hydrolysis activity
MF	Low DO	SUI3	2	0.615	9.310E-02	mRNA binding; tRNA binding
MF	Low DO	SUL1	1	2.478	3.045E-14	sulfur compound transmembrane transporter activity
MF	Low DO	MMP1	1	2.406	1.403E-14	sulfur compound transmembrane transporter activity
MF	Low DO	RMT2	1	2.371	3.615E-07	N-methyltransferase activity
MF	Low DO	IMD4	1	2.271	9.307E-07	mRNA binding
MF	Low DO	ECM1	1	2.230	1.057E-07	ribonucleoprotein complex binding
MF	Low DO	NOP14	1	2.108	3.172E-08	U3 snoRNA binding
MF	Low DO	RRP12	1	2.096	2.843E-10	mRNA binding
MF	Low DO	UTP10	1	2.092	1.431E-12	U3 snoRNA binding
MF	Low DO	SPB1	1	2.069	7.963E-11	rRNA methyltransferase activity
MF	Low DO	YTM1	1	2.017	5.938E-08	ribonucleoprotein complex binding
MF	Low DO	YVH1	1	1.957	1.159E-07	ribonucleoprotein complex binding
MF	Low DO	URB1	1	1.947	1.149E-08	mRNA binding
MF	Low DO	NUG1	1	1.904	1.790E-07	mRNA binding
MF	Low DO	SUL2	1	1.895	3.543E-09	sulfur compound transmembrane transporter activity
MF	Low DO	UTP20	1	1.894	1.149E-08	mRNA binding
MF	Low DO	RPF1	1	1.876	2.548E-05	rRNA primary transcript binding
MF	Low DO	RPA190	1	1.840	2.602E-08	DNA-directed RNA polymerase activity
MF	Low DO	ATM1	1	1.823	1.908E-07	ATP hydrolysis activity
MF	Low DO	RPA135	1	1.792	1.317E-07	DNA-directed RNA polymerase activity
MF	Low DO	CYS4	1	1.787	1.180E-06	mRNA binding
MF	Low DO	IMP4	1	1.762	1.290E-05	rRNA primary transcript binding
MF	Low DO	MAK21	1	1.761	6.954E-09	mRNA binding
MF	Low DO	BRX1	1	1.757	1.745E-05	rRNA primary transcript binding
MF	Low DO	MRT4	1	1.726	3.613E-05	large ribosomal subunit rRNA binding
MF	Low DO	SRO9	1	1.720	2.062E-05	mRNA binding
MF	Low DO	RPA49	1	1.683	5.365E-06	DNA-directed RNA polymerase activity
MF	Low DO	NOP56	1	1.674	3.932E-06	mRNA binding
MF	Low DO	TSR2	1	1.666	8.579E-06	ribonucleoprotein complex binding
MF	Low DO	TRM3	1	1.652	1.331E-07	tRNA methyltransferase activity
MF	Low DO	PWP2	1	1.632	4.779E-06	mRNA binding
MF	Low DO	NSR1	1	1.596	2.082E-04	mRNA binding
MF	Low DO	DIP2	1	1.593	7.082E-06	U3 snoRNA binding
MF	Low DO	KRE33	1	1.559	1.379E-06	tRNA binding
MF	Low DO	TSR1	1	1.556	5.503E-06	ribonucleoprotein complex binding
MF	Low DO	SOF1	1	1.535	5.674E-05	mRNA binding
MF	Low DO	SUA5	1	1.519	8.695E-05	tRNA binding
MF	Low DO	EBP2	1	1.504	3.927E-06	mRNA binding
MF	Low DO	DUS3	1	1.501	7.563E-06	mRNA binding
MF	Low DO	NOC2	1	1.496	1.964E-05	mRNA binding
MF	Low DO	LYS1	1	1.494	9.773E-06	mRNA binding
MF	Low DO	CBF5	1	1.493	1.235E-05	mRNA binding
MF	Low DO	BUD23	1	1.483	1.730E-05	rRNA methyltransferase activity
MF	Low DO	RPA34	1	1.482	1.257E-04	DNA-directed RNA polymerase activity
MF	Low DO	DUR3	1	1.479	5.877E-04	organic cation transmembrane transporter activity
MF	Low DO	PNO1	1	1.474	7.848E-05	rRNA primary transcript binding
MF	Low DO	IKI3	1	1.468	7.765E-06	tRNA binding
MF	Low DO	RPC40	1	1.433	9.154E-05	DNA-directed RNA polymerase activity
MF	Low DO	TRM13	1	1.429	4.527E-05	tRNA methyltransferase activity

MF	Low DO	EFM2	1	1.426	9.353E-06	N-methyltransferase activity
MF	Low DO	UTP13	1	1.413	8.555E-06	U3 snoRNA binding
MF	Low DO	NOP9	1	1.411	2.572E-05	mRNA binding
MF	Low DO	NIP1	1	1.409	2.683E-05	mRNA binding
MF	Low DO	AFG2	1	1.391	4.921E-05	ATP hydrolysis activity
MF	Low DO	MEU1	1	1.376	2.111E-04	mRNA binding
MF	Low DO	NRP1	1	1.376	3.993E-05	mRNA binding
MF	Low DO	EFM4	1	1.374	1.007E-04	N-methyltransferase activity
MF	Low DO	GCD10	1	1.354	1.823E-04	tRNA methyltransferase activity
MF	Low DO	REA1	1	1.350	1.719E-05	ATP hydrolysis activity
MF	Low DO	CNS1	1	1.345	2.123E-05	ribonucleoprotein complex binding
MF	Low DO	UTP6	1	1.336	1.065E-03	U3 snoRNA binding
MF	Low DO	BUD27	1	1.333	1.577E-05	mRNA binding
MF	Low DO	ANB1	1	1.326	7.840E-02	ribonucleoprotein complex binding
MF	Low DO	BUD21	1	1.310	5.550E-04	U3 snoRNA binding
MF	Low DO	RET1	1	1.308	2.390E-05	DNA-directed RNA polymerase activity
MF	Low DO	BMT2	1	1.300	4.835E-04	rRNA methyltransferase activity
MF	Low DO	ENA1	1	1.299	6.845E-04	ATP hydrolysis activity
MF	Low DO	RRP8	1	1.298	2.269E-04	rRNA methyltransferase activity
MF	Low DO	TRM7	1	1.296	2.082E-04	tRNA methyltransferase activity
MF	Low DO	RTT10	1	1.295	1.341E-04	tRNA binding
MF	Low DO	PPM2	1	1.263	5.154E-04	tRNA methyltransferase activity
MF	Low DO	RPC19	1	1.262	8.796E-04	DNA-directed RNA polymerase activity
MF	Low DO	NSA1	1	1.255	2.082E-04	ribonucleoprotein complex binding
MF	Low DO	NOP15	1	1.236	1.397E-03	ribonucleoprotein complex binding
MF	Low DO	RPC25	1	1.212	2.503E-03	DNA-directed RNA polymerase activity
MF	Low DO	HPM1	1	1.210	2.318E-03	N-methyltransferase activity
MF	Low DO	PUS1	1	1.209	1.576E-03	mRNA binding
MF	Low DO	SSB1	1	1.207	1.038E-03	ATP hydrolysis activity
MF	Low DO	RPC17	1	1.203	2.295E-03	DNA-directed RNA polymerase activity
MF	Low DO	NOG2	1	1.168	2.434E-03	large ribosomal subunit rRNA binding
MF	Low DO	RIA1	1	1.161	4.340E-04	ribonucleoprotein complex binding
MF	Low DO	UTP15	1	1.158	3.430E-03	U3 snoRNA binding
MF	Low DO	ELP3	1	1.155	1.241E-03	tRNA binding
MF	Low DO	RRP6	1	1.149	3.084E-03	rRNA primary transcript binding
MF	Low DO	UTP25	1	1.129	4.560E-04	U3 snoRNA binding
MF	Low DO	CDC60	1	1.123	3.179E-04	mRNA binding
MF	Low DO	GCD14	1	1.109	2.121E-03	tRNA methyltransferase activity
MF	Low DO	EMG1	1	1.098	3.585E-03	rRNA methyltransferase activity
MF	Low DO	NOP2	1	1.092	2.737E-03	rRNA methyltransferase activity
MF	Low DO	RPA12	1	1.086	2.754E-03	DNA-directed RNA polymerase activity
MF	Low DO	BUD20	1	1.084	8.811E-03	ribonucleoprotein complex binding
MF	Low DO	PMA1	1	1.081	6.610E-03	ATP hydrolysis activity
MF	Low DO	NOP58	1	1.080	1.167E-03	mRNA binding
MF	Low DO	RPC53	1	1.078	2.016E-03	DNA-directed RNA polymerase activity
MF	Low DO	RPC31	1	1.066	3.561E-03	DNA-directed RNA polymerase activity
MF	Low DO	NPA3	1	1.057	1.267E-03	ATP hydrolysis activity
MF	Low DO	TAT1	1	1.052	1.115E-02	organic cation transmembrane transporter activity
MF	Low DO	MES1	1	1.048	1.353E-03	mRNA binding
MF	Low DO	ARB1	1	1.045	3.992E-03	ATP hydrolysis activity
MF	Low DO	RCM1	1	1.040	1.562E-03	rRNA methyltransferase activity
MF	Low DO	EFM1	1	1.034	2.645E-03	N-methyltransferase activity
MF	Low DO	RPO26	1	1.031	6.680E-03	DNA-directed RNA polymerase activity
MF	Low DO	SSZ1	1	1.015	7.890E-03	ATP hydrolysis activity
MF	Low DO	PUF4	1	1.011	1.841E-02	mRNA binding
MF	Low DO	PAB1	1	1.007	8.236E-02	mRNA binding
MF	Low DO	NCS2	1	1.007	3.603E-03	tRNA binding
MF	Low DO	POL5	1	1.006	2.702E-03	rRNA primary transcript binding
MF	Low DO	RPC34	1	0.998	5.678E-03	DNA-directed RNA polymerase activity
MF	Low DO	WRS1	1	0.990	4.491E-03	mRNA binding
MF	Low DO	HMT1	1	0.990	1.883E-02	N-methyltransferase activity

MF	Low DO	SFM1	1	0.987	1.288E-02	N-methyltransferase activity
MF	Low DO	RPA43	1	0.980	9.251E-03	DNA-directed RNA polymerase activity
MF	Low DO	TAT2	1	0.978	1.231E-02	organic cation transmembrane transporter activity
MF	Low DO	EFM3	1	0.976	4.463E-03	N-methyltransferase activity
MF	Low DO	RPC37	1	0.972	6.836E-03	DNA-directed RNA polymerase activity
MF	Low DO	YDR341C	1	0.967	5.898E-03	mRNA binding
MF	Low DO	RKM4	1	0.952	1.763E-02	N-methyltransferase activity
MF	Low DO	OAC1	1	0.949	1.421E-02	sulfur compound transmembrane transporter activity
MF	Low DO	GIS2	1	0.946	1.210E-02	mRNA binding
MF	Low DO	VAS1	1	0.942	8.986E-03	mRNA binding
MF	Low DO	SUP45	1	0.936	4.239E-03	mRNA binding
MF	Low DO	RPC82	1	0.933	9.403E-03	DNA-directed RNA polymerase activity
MF	Low DO	RPB5	1	0.927	1.001E-02	DNA-directed RNA polymerase activity
MF	Low DO	TEF1	1	0.926	1.519E-02	ribonucleoprotein complex binding
MF	Low DO	RPC11	1	0.924	1.472E-02	DNA-directed RNA polymerase activity
MF	Low DO	THS1	1	0.923	1.451E-02	mRNA binding
MF	Low DO	MCM2	1	0.919	6.999E-03	ATP hydrolysis activity
MF	Low DO	RBS1	1	0.916	2.573E-02	mRNA binding
MF	Low DO	DOM34	1	0.912	2.622E-02	ribonucleoprotein complex binding
MF	Low DO	DIS3	1	0.910	5.619E-03	tRNA binding
MF	Low DO	TIF4631	1	0.909	5.134E-03	mRNA binding
MF	Low DO	TRM44	1	0.902	1.103E-02	tRNA methyltransferase activity
MF	Low DO	CAN1	1	0.900	1.450E-02	organic cation transmembrane transporter activity
MF	Low DO	SNU13	1	0.898	1.066E-02	U3 snoRNA binding
MF	Low DO	HTS1	1	0.896	6.453E-03	mRNA binding
MF	Low DO	MCM5	1	0.895	1.010E-02	ATP hydrolysis activity
MF	Low DO	CFD1	1	0.893	1.684E-02	ATP hydrolysis activity
MF	Low DO	FUN30	1	0.892	7.976E-03	ATP hydrolysis activity
MF	Low DO	IMD3	1	0.889	8.058E-03	mRNA binding
MF	Low DO	SOA1	1	0.885	1.461E-02	sulfur compound transmembrane transporter activity
MF	Low DO	MUD2	1	0.881	1.837E-02	mRNA binding
MF	Low DO	GLN4	1	0.877	1.253E-02	mRNA binding
MF	Low DO	RKM2	1	0.876	1.404E-02	N-methyltransferase activity
MF	Low DO	HNM1	1	0.873	2.844E-02	organic cation transmembrane transporter activity
MF	Low DO	RRP17	1	0.853	2.379E-02	ribonucleoprotein complex binding
MF	Low DO	RAT1	1	0.844	1.062E-02	mRNA binding
MF	Low DO	ENA5	1	0.841	2.539E-02	ATP hydrolysis activity
MF	Low DO	ORT1	1	0.839	4.492E-02	organic cation transmembrane transporter activity
MF	Low DO	RPC10	1	0.839	2.849E-02	DNA-directed RNA polymerase activity
MF	Low DO	RRP45	1	0.834	3.735E-02	mRNA binding
MF	Low DO	CCT5	1	0.810	3.187E-02	ATP hydrolysis activity
MF	Low DO	RPO31	1	0.807	2.300E-02	DNA-directed RNA polymerase activity
MF	Low DO	SMC3	1	0.804	4.978E-02	ATP hydrolysis activity
MF	Low DO	VHT1	1	0.790	2.269E-02	sulfur compound transmembrane transporter activity
MF	Low DO	BRE2	1	0.788	2.783E-02	N-methyltransferase activity
MF	Low DO	SNM1	1	0.788	2.676E-02	rRNA primary transcript binding
MF	Low DO	YIH1	1	0.780	4.067E-02	ribonucleoprotein complex binding
MF	Low DO	CAF16	1	0.776	5.261E-02	ATP hydrolysis activity
MF	Low DO	RPL35B	1	0.760	6.513E-02	mRNA binding
MF	Low DO	RTC2	1	0.759	1.114E-01	organic cation transmembrane transporter activity
MF	Low DO	RRP42	1	0.758	4.820E-02	mRNA binding
MF	Low DO	RIO1	1	0.754	3.175E-02	ATP hydrolysis activity
MF	Low DO	RPL23A	1	0.753	5.407E-02	large ribosomal subunit rRNA binding
MF	Low DO	PDR5	1	0.737	3.923E-02	ATP hydrolysis activity
MF	Low DO	IMD2	1	0.736	6.964E-02	mRNA binding
MF	Low DO	RRP43	1	0.727	6.221E-02	mRNA binding
MF	Low DO	CCT3	1	0.724	3.527E-02	ATP hydrolysis activity
MF	Low DO	PDR12	1	0.719	3.841E-02	ATP hydrolysis activity
MF	Low DO	RPS26B	1	0.719	4.492E-02	mRNA binding
MF	Low DO	LHS1	1	0.712	4.047E-02	ATP hydrolysis activity
MF	Low DO	MCM3	1	0.705	7.979E-02	ATP hydrolysis activity

MF	Low DO	SNU56	1	0.696	7.301E-02	mRNA binding
MF	Low DO	IRC5	1	0.692	1.174E-01	ATP hydrolysis activity
MF	Low DO	FUN12	1	0.690	5.433E-02	ribonucleoprotein complex binding
MF	Low DO	TRM12	1	0.688	6.148E-02	tRNA methyltransferase activity
MF	Low DO	CRS1	1	0.685	6.406E-02	mRNA binding
MF	Low DO	MCX1	1	0.683	6.980E-02	ATP hydrolysis activity
MF	Low DO	HSH155	1	0.682	5.726E-02	mRNA binding
MF	Low DO	RSA1	1	0.673	5.821E-02	ribonucleoprotein complex binding
MF	Low DO	RVB1	1	0.670	6.806E-02	ATP hydrolysis activity
MF	Low DO	RPL24B	1	0.669	9.638E-02	mRNA binding
MF	Low DO	NCS6	1	0.662	7.143E-02	tRNA binding
MF	Low DO	CCT2	1	0.660	7.036E-02	ATP hydrolysis activity
MF	Low DO	TRM5	1	0.651	9.185E-02	tRNA methyltransferase activity
MF	Low DO	RNA15	1	0.641	9.352E-02	mRNA binding
MF	Low DO	HRB1	1	0.629	8.990E-02	mRNA binding
MF	Low DO	QDR2	1	0.625	1.302E-01	organic cation transmembrane transporter activity
MF	Low DO	ASC1	1	0.623	1.077E-01	ribonucleoprotein complex binding
MF	Low DO	RPP1A	1	0.622	1.644E-01	ribonucleoprotein complex binding
MF	Low DO	SRP72	1	0.621	8.235E-02	ribonucleoprotein complex binding
MF	Low DO	BRE5	1	0.619	8.681E-02	mRNA binding
MF	Low DO	GCN1	1	0.614	9.382E-02	ribonucleoprotein complex binding
MF	Low DO	FAP7	1	0.609	1.108E-01	ATP hydrolysis activity
MF	Low DO	PRP16	1	0.608	8.841E-02	ATP hydrolysis activity
MF	Low DO	HIP1	1	0.604	1.083E-01	organic cation transmembrane transporter activity
MF	Low DO	KRS1	1	0.603	1.270E-01	tRNA binding
MF	Low DO	YCF1	1	0.598	1.079E-01	ATP hydrolysis activity
MF	Low DO	TCP1	1	0.592	1.143E-01	ATP hydrolysis activity
MF	Low DO	TIF2	1	0.576	1.248E-01	ATP hydrolysis activity
MF	Low DO	TIF6	1	0.573	1.685E-01	ribonucleoprotein complex binding
MF	Low DO	CCT4	1	0.561	1.262E-01	ATP hydrolysis activity
MF	Low DO	TIF11	1	0.554	1.434E-01	ribonucleoprotein complex binding
MF	Low DO	LOS1	1	0.548	1.542E-01	tRNA binding
MF	Low DO	YLH47	1	0.546	1.515E-01	ribonucleoprotein complex binding
MF	Low DO	HBS1	1	0.533	1.666E-01	ribonucleoprotein complex binding

Supplementary Table S6.

Model-estimated mismatch rates and Low-DO-associated fold changes across substitution classes.

SBS-6 class	MR_H	SE_H	MR_L	SE_L	MR FC LvsH	SE_FC apprx	asymp LCL	asymp UCL	z.ratio	p_holm	p_label
C>T	1.41E-03	1.83E-05	1.52E-03	1.94E-05	1.074	1.29E-02	1.051	1.098	6.41	1.44E-10	***
C>A	9.45E-04	1.34E-05	9.31E-04	1.32E-05	0.986	1.36E-02	0.959	1.013	-1.03	3.04E-01	
C>G	4.88E-04	8.31E-06	5.12E-04	8.60E-06	1.051	2.11E-02	1.012	1.091	2.58	9.96E-03	*
T>A	7.65E-04	1.14E-05	8.09E-04	1.19E-05	1.058	1.71E-02	1.027	1.090	3.69	2.28E-04	***
T>C	1.25E-03	1.65E-05	1.31E-03	1.72E-05	1.053	1.33E-02	1.029	1.078	4.35	1.39E-05	***
T>G	6.72E-04	1.04E-05	6.82E-04	1.05E-05	1.016	1.70E-02	0.984	1.049	0.97	3.32E-01	

Supplementary Table S7.

Model-estimated variant rates and Low-DO-associated fold changes across substitution classes.

SBS-6 class	VR_H	SE_H	VR_L	SE_L	VR FC LvsH	FC_SE	asymp. LCL	asymp. UCL	z.ratio	p_holm	p_label
C>T	1.68E-03	5.49E-05	1.83E-03	5.95E-05	1.087	3.34E-02	1.023	1.154	2.708	6.78E-03	*
C>A	3.35E-04	1.19E-05	3.56E-04	1.26E-05	1.065	3.88E-02	0.992	1.144	1.731	8.35E-02	
C>G	2.84E-04	1.02E-05	2.86E-04	1.03E-05	1.010	3.81E-02	0.938	1.088	0.271	7.86E-01	
T>A	3.39E-04	1.16E-05	3.80E-04	1.29E-05	1.120	3.77E-02	1.048	1.196	3.355	7.93E-04	**
T>C	7.79E-04	2.56E-05	8.86E-04	2.91E-05	1.138	3.55E-02	1.070	1.210	4.136	3.54E-05	***
T>G	2.62E-04	9.11E-06	2.64E-04	9.18E-06	1.008	3.54E-02	0.941	1.080	0.221	8.25E-01	

Supplementary Table S8.

Per-sample Mutect2-derived RNA-seq candidate-site rates shown before and after removal of phase-shared candidate sites.

filter_status	sample	replicate	condition	SBS6	n_variants	callable_ref	mutation_rate
Before shared-site subtraction	WRS_1_H	1	H	C>A	1344	4120134	3.26E-04
Before shared-site subtraction	WRS_1_L	1	L	C>A	1429	4000761	3.57E-04
Before shared-site subtraction	WRS_2_H	2	H	C>A	1302	4095768	3.18E-04
Before shared-site subtraction	WRS_2_L	2	L	C>A	1353	4073278	3.32E-04
Before shared-site subtraction	WRS_3_H	3	H	C>A	1465	4063975	3.60E-04
Before shared-site subtraction	WRS_3_L	3	L	C>A	1569	4121881	3.81E-04
Before shared-site subtraction	WRS_1_H	1	H	C>G	1170	4120134	2.84E-04
Before shared-site subtraction	WRS_1_L	1	L	C>G	1153	4000761	2.88E-04
Before shared-site subtraction	WRS_2_H	2	H	C>G	1112	4095768	2.71E-04
Before shared-site subtraction	WRS_2_L	2	L	C>G	1139	4073278	2.80E-04
Before shared-site subtraction	WRS_3_H	3	H	C>G	1202	4063975	2.96E-04
Before shared-site subtraction	WRS_3_L	3	L	C>G	1203	4121881	2.92E-04
Before shared-site subtraction	WRS_1_H	1	H	C>T	6829	4120134	1.66E-03
Before shared-site subtraction	WRS_1_L	1	L	C>T	7103	4000761	1.78E-03
Before shared-site subtraction	WRS_2_H	2	H	C>T	6519	4095768	1.59E-03
Before shared-site subtraction	WRS_2_L	2	L	C>T	7100	4073278	1.74E-03
Before shared-site subtraction	WRS_3_H	3	H	C>T	7305	4063975	1.80E-03
Before shared-site subtraction	WRS_3_L	3	L	C>T	8102	4121881	1.97E-03
Before shared-site subtraction	WRS_1_H	1	H	T>A	2226	6650367	3.35E-04
Before shared-site subtraction	WRS_1_L	1	L	T>A	2516	6475373	3.89E-04
Before shared-site subtraction	WRS_2_H	2	H	T>A	2101	6616913	3.18E-04
Before shared-site subtraction	WRS_2_L	2	L	T>A	2375	6584104	3.61E-04
Before shared-site subtraction	WRS_3_H	3	H	T>A	2413	6564066	3.68E-04
Before shared-site subtraction	WRS_3_L	3	L	T>A	2607	6653815	3.92E-04
Before shared-site subtraction	WRS_1_H	1	H	T>C	5971	6650367	8.98E-04
Before shared-site subtraction	WRS_1_L	1	L	T>C	5592	6475373	8.64E-04
Before shared-site subtraction	WRS_2_H	2	H	T>C	4487	6616913	6.78E-04
Before shared-site subtraction	WRS_2_L	2	L	T>C	5561	6584104	8.45E-04
Before shared-site subtraction	WRS_3_H	3	H	T>C	5016	6564066	7.64E-04
Before shared-site subtraction	WRS_3_L	3	L	T>C	6340	6653815	9.53E-04
Before shared-site subtraction	WRS_1_H	1	H	T>G	1757	6650367	2.64E-04
Before shared-site subtraction	WRS_1_L	1	L	T>G	1751	6475373	2.70E-04
Before shared-site subtraction	WRS_2_H	2	H	T>G	1623	6616913	2.45E-04
Before shared-site subtraction	WRS_2_L	2	L	T>G	1699	6584104	2.58E-04
Before shared-site subtraction	WRS_3_H	3	H	T>G	1818	6564066	2.77E-04
Before shared-site subtraction	WRS_3_L	3	L	T>G	1752	6653815	2.63E-04
After shared-site subtraction	WRS_1_H	1	H	C>A	843	4120134	2.05E-04
After shared-site subtraction	WRS_1_L	1	L	C>A	925	4000761	2.31E-04
After shared-site subtraction	WRS_2_H	2	H	C>A	809	4095768	1.98E-04
After shared-site subtraction	WRS_2_L	2	L	C>A	871	4073278	2.14E-04
After shared-site subtraction	WRS_3_H	3	H	C>A	953	4063975	2.34E-04
After shared-site subtraction	WRS_3_L	3	L	C>A	1031	4121881	2.50E-04
After shared-site subtraction	WRS_1_H	1	H	C>G	682	4120134	1.66E-04
After shared-site subtraction	WRS_1_L	1	L	C>G	685	4000761	1.71E-04
After shared-site subtraction	WRS_2_H	2	H	C>G	602	4095768	1.47E-04
After shared-site subtraction	WRS_2_L	2	L	C>G	621	4073278	1.52E-04
After shared-site subtraction	WRS_3_H	3	H	C>G	681	4063975	1.68E-04
After shared-site subtraction	WRS_3_L	3	L	C>G	699	4121881	1.70E-04
After shared-site subtraction	WRS_1_H	1	H	C>T	3702	4120134	8.99E-04
After shared-site subtraction	WRS_1_L	1	L	C>T	4032	4000761	1.01E-03
After shared-site subtraction	WRS_2_H	2	H	C>T	3371	4095768	8.23E-04
After shared-site subtraction	WRS_2_L	2	L	C>T	3981	4073278	9.77E-04
After shared-site subtraction	WRS_3_H	3	H	C>T	3988	4063975	9.81E-04
After shared-site subtraction	WRS_3_L	3	L	C>T	4703	4121881	1.14E-03

After shared-site subtraction	WRS_1_H	1	H	T>A	1513	6650367	2.28E-04
After shared-site subtraction	WRS_1_L	1	L	T>A	1805	6475373	2.79E-04
After shared-site subtraction	WRS_2_H	2	H	T>A	1383	6616913	2.09E-04
After shared-site subtraction	WRS_2_L	2	L	T>A	1650	6584104	2.51E-04
After shared-site subtraction	WRS_3_H	3	H	T>A	1643	6564066	2.50E-04
After shared-site subtraction	WRS_3_L	3	L	T>A	1832	6653815	2.75E-04
After shared-site subtraction	WRS_1_H	1	H	T>C	4587	6650367	6.90E-04
After shared-site subtraction	WRS_1_L	1	L	T>C	4445	6475373	6.86E-04
After shared-site subtraction	WRS_2_H	2	H	T>C	3417	6616913	5.16E-04
After shared-site subtraction	WRS_2_L	2	L	T>C	4349	6584104	6.61E-04
After shared-site subtraction	WRS_3_H	3	H	T>C	3869	6564066	5.89E-04
After shared-site subtraction	WRS_3_L	3	L	T>C	5015	6653815	7.54E-04
After shared-site subtraction	WRS_1_H	1	H	T>G	1188	6650367	1.79E-04
After shared-site subtraction	WRS_1_L	1	L	T>G	1182	6475373	1.83E-04
After shared-site subtraction	WRS_2_H	2	H	T>G	1030	6616913	1.56E-04
After shared-site subtraction	WRS_2_L	2	L	T>G	1123	6584104	1.71E-04
After shared-site subtraction	WRS_3_H	3	H	T>G	1200	6564066	1.83E-04
After shared-site subtraction	WRS_3_L	3	L	T>G	1156	6653815	1.74E-04

Supplementary Table S9.

Composition of the de-collapsed C>T class to C>T and G>A events analyzed separately.

sample	total_count_CT	total_count_GA	p_CT	p_GA
WRS_1_H	1558799313	1559616227	49.99%	50.01%
WRS_1_L	1191361793	1184033618	50.15%	49.85%
WRS_2_H	1308970067	1305802438	50.06%	49.94%
WRS_2_L	1166657502	1160371019	50.14%	49.86%
WRS_3_H	1160703664	1159645183	50.02%	49.98%
WRS_3_L	1566824957	1562973534	50.06%	49.94%
